# Genome-enabled discovery of anthraquinone biosynthesis in *Senna tora*

**DOI:** 10.1038/s41467-020-19681-1

**Published:** 2020-11-18

**Authors:** Sang-Ho Kang, Ramesh Prasad Pandey, Chang-Muk Lee, Joon-Soo Sim, Jin-Tae Jeong, Beom-Soon Choi, Myunghee Jung, Daniel Ginzburg, Kangmei Zhao, So Youn Won, Tae-Jin Oh, Yeisoo Yu, Nam-Hoon Kim, Ok Ran Lee, Tae-Ho Lee, Puspalata Bashyal, Tae-Su Kim, Woo-Haeng Lee, Charles Hawkins, Chang-Kug Kim, Jung Sun Kim, Byoung Ohg Ahn, Seung Yon Rhee, Jae Kyung Sohng

**Affiliations:** 1 Genomics Division, National Institute of Agricultural Sciences, RDA, Jeonju, 54874 Republic of Korea; 2 Department of Pharmaceutical Engineering and Biotechnology, Sun Moon University, Asan, 31460 Republic of Korea; 3 Metabolic Engineering Division, National Institute of Agricultural Sciences, RDA, Jeonju, 54874 Republic of Korea; 4 Department of Herbal Crop Research, National Institute of Horticultural and Herbal Science, RDA, Eumseong, 55365 Republic of Korea; 5 Phyzen Genomics Institute, Seongnam, 13488 Republic of Korea; 6 Department of Forest Science, College of Agriculture and Life Science, Seoul National University, Seoul, 08826 Republic of Korea; 7 Department of Plant Biology, Carnegie Institution for Science, Stanford, CA 94305 USA; 8 Department of Applied Plant Science, College of Agriculture and Life Science, Chonnam National University, Gwangju, 61186 Republic of Korea; 9 Present Address: Department of Biological Engineering, Massachusetts Institute of Technology, Cambridge, MA 02139 USA; 10 Present Address: DNACARE Co. Ltd, Seoul, 06730 Republic of Korea

**Keywords:** Genomics, Plant evolution, Secondary metabolism

## Abstract

*Senna tora* is a widely used medicinal plant. Its health benefits have been attributed to the large quantity of anthraquinones, but how they are made in plants remains a mystery. To identify the genes responsible for plant anthraquinone biosynthesis, we reveal the genome sequence of *S. tora* at the chromosome level with 526 Mb (96%) assembled into 13 chromosomes. Comparison among related plant species shows that a chalcone synthase-like (CHS-L) gene family has lineage-specifically and rapidly expanded in *S. tora*. Combining genomics, transcriptomics, metabolomics, and biochemistry, we identify a CHS-L gene contributing to the biosynthesis of anthraquinones. The *S. tora* reference genome will accelerate the discovery of biologically active anthraquinone biosynthesis pathways in medicinal plants.

## Abstract (web summary)

Anthraquinones are aromatic polyketides and have been used for treating various diseases, but the biosynthetic pathway is unclear. Here, the authors assemble the genome of an anthraquinone-producing medicinal plant *Senna tora* and show the evidences that CHS-like genes may be involved in anthraquinone biosynthesis.

## Introduction

*Senna tora* (L.) Roxb., also known as *Cassia tora*, is a favorite of ancient Chinese and Ayurvedic herbal medicine that is now widely used around the world and recorded as Model List of Essential Medicines by the World Health Organization^1^. Recent studies point to *S. tora*’s beneficial activities against microbial^2–5^ and parasitic^6^ infections, prevention or delay of the onset of neurodegenerative diseases^7,8^, and diabetes^9^. *S. tora*’s positive health impact is attributed to the significant amount of anthraquinones in mature seeds and other parts of the plant^10–12^. As an ancient medicine, anthraquinones from seeds of other *Senna* species are commonly used for treating various diseases^13^. Despite the extensive applications of *Senna* plants in medicine and industry^1^, molecular and genomic studies of this remarkable genus of plants have been limited^14–17^. Elucidating the genes responsible for the biosynthesis of anthraquinones in *S. tora* will aid molecular breeding and the development of tools for probing its biochemistry.

Anthraquinones are aromatic polyketides made by bacteria, fungi, insects, and plants^18,19^. Besides their medicinal benefits, natural anthraquinones are garnering attention as alternatives to synthetic dyes that damage aquatic ecosystems^20–22^. Bacteria, fungi, and insects make anthraquinones via a polyketide pathway using type I or II polyketide synthases^18,23^.

For plants, how anthraquinones are made remains unknown. Two biosynthesis pathways have been proposed for anthraquinones in plants: (1) a polyketide pathway^24^ and (2) a combination of shikimate and mevalonate/methyl-D-erythritol 4-phosphate pathways^25,26^. More than three decades ago, radiolabeled feeding experiments indicated that the A and B rings of anthraquinones were derived from shikimate and *α*-ketoglutarate via *O*-succinylbenzoate^27–29^ and C ring from mevalonate pathway via isopentenyl pyrophosphate (IPP) and dimethylallyl pyrophosphate (DMAPP)^25,26,30^ or 2-C-methyl-D-erythritol 4-phosphate (MEP) pathway^31,32^. Contrarily, recent studies speculated biosynthesis of anthraquinones in plants to occur via a polyketide pathway^33–35^. Type III polyketide synthase (PKS) enzymes could actively catalyze seven successive decarboxylative condensations of malonyl-CoA to produce an octaketide chain^34,35^. The linear polyketide chain undergoes cyclization and decarboxylation reactions to produce the core unit of polyketides such as atrochrysone carboxylic acid followed by decarboxylation to atrochrysone and dehydration to emodin anthrone^33–37^ (Supplementary Fig. 1). However, to date, no study in type III PKS enzymes has provided conclusive evidence on the biosynthesis of anthraquinones or the intermediate metabolites of the pathways. Beerhues and colleagues^33^ showed promising outcomes on the biosynthesis of an anthranoid scaffold via the polyketide pathway. The in vitro reaction using acetyl-CoA, stable carbon isotope-labeled malonyl-CoA, and cell-free extracts of *Cassia bicapsularis* cell cultures produced emodin anthrone and *O*-methylated torochrysone^33^. However, this study could not discern whether a PKS was involved in the biosynthesis of the anthranoid scaffolds.

In this work, we present a high-quality reference genome of *S. tora* cultivar Myeongyun, examine the evolution of candidate gene families involved in anthraquinone biosynthesis, and identify the enzyme known to catalyze a plant anthraquinone. By combining genomic, transcriptomic, metabolomic, and biochemical approaches, we systematically screen and identify a putative gene responsible for biosynthesis of an anthraquinone scaffold in *S. tora*.

## Results and discussion

### *S. tora* genome assembly and annotation

We generated one of the highest-quality genomes for medicinal plants. The *S. tora* cultivar Myeongyun genome was assembled with Pacific Biosciences long-read sequencing (146.2× coverage) by FALCON v0.4 (Supplementary Tables 1 and 2). To improve the quality of genome assembly, we performed error correction with Sequel data by Arrow v2.1.0 and further corrected it with 101.2× Illumina data using BWA and GATK. *S. tora* has an estimated genome size of ~547 Mb based on *k*-mer analysis (Supplementary Fig. 2). Through chromosome conformation capture (Hi-C) mapping, we generated 13 chromosome-scale scaffolds (hereafter called chromosomes, Chr1–Chr13) totaling 502.6 Mb, 95.5% of the ~526.4 Mb of the assembled genome (Table 1 and Supplementary Figs. 3 and 4). We evaluated the quality of assembly using Benchmarking Universal Single Copy Orthologs (BUSCO)^38^, sequencing of 10 bacterial artificial chromosome (BAC) clones, and comparing to a linkage map (Supplementary Fig. 5). BUSCO estimates 94.3% completeness (Supplementary Table 3), suggesting that the assembly includes most of the *S. tora* gene space. BAC sequence alignments showed high mapping rates (99.8%) with the assemblies (Supplementary Fig. 6 and Supplementary Table 4). We also built a genetic map of diploid *S. tora*, to which 401.1 Mb of the assembled scaffolds were mapped (Supplementary Fig. 7). The 13 linkage groups matched well to the 13 chromosomes, indicating the high quality of *S. tora* genome assembly (Supplementary Fig. 8).

**Table 1:** Summary of genome assembly and protein-coding genes in *S. tora*.

	Contigs	Superscaffolds
*Assembly features*		
Numbers	721	13
Total length	526.3 Mb	502.6 Mb
N50	4.03 Mb	41.7 Mb
Longest	14.9 Mb	52.7 Mb
GC content	35.45%	35.45%
*Protein-coding genes*		
No. of genes		45,268
Mean gene length		3162 bp
Mean exon length		217 bp
Mean intron length		656 bp
*Noncoding genes*		
lncRNA		3278
rRNA		752
tRNA		839

**Fig. 1:**
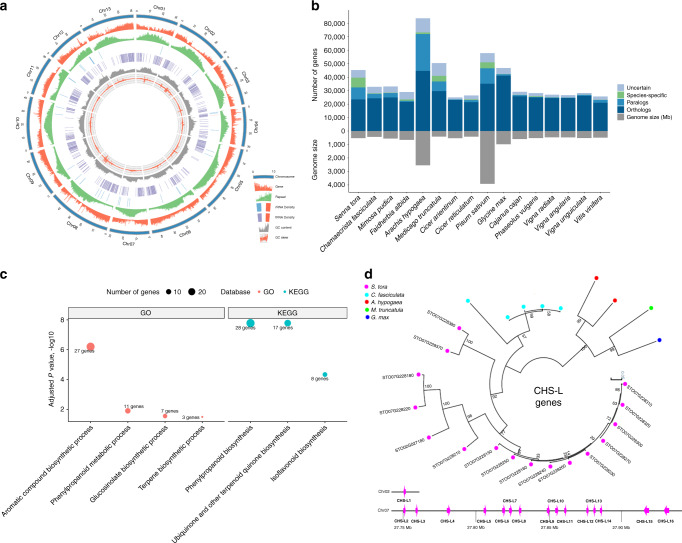
The *S*. *tora* genome and comparative genomic analysis. **a** The landscape of genome assembly (~502 Mb) and annotation of *S. tora*. Tracks (from outside) correspond to chromosomes (Chr01–Chr13 on a Mb scale), gene density, repeat density, rRNA density, tRNA density, GC content, and GC skew. Tracks are drawn in nonoverlapping 100-kb sliding windows. The red bars in the rRNA and tRNA tracks represent the maximum density of copies on the scale. **b** An overview of orthologous and paralogous genes among *S. tora*, related legumes, and *V. vinifera*. “Uncertain” indicates homologous genes obtained from BLAST but not found using OrthoMCL. “Species-specific” genes do not have any similarity to genes in the other species based on BLAST and OrthoMCL. **c** Significantly enriched biological process GO and KEGG categories (specialized metabolism) of expanded gene families in *S. tora*. **d** Lineage-specific expansion of the CHS-L gene family in *S. tora* and four other legumes. The 15 tandemly duplicated gene clusters are ordered and shown on chromosome 7, as well as one gene on chromosome 2.

*S. tora*’s genomic content is consistent with other sequenced plant genomes. A total of 45,268 genes were annotated with the average gene length (3,157 bp), exon sequence length (217 bp with 4.37 exons per gene), and intron length (655 bp) that were similar to those of other legume species (Table 1 and Supplementary Fig. 9). Among the protein-coding genes, 31,010 (68.50%) showed homology to characterized genes based on BLAST searches and 25,453 (56.23%) and 17,450 (38.55%) were assigned to Gene Ontology (GO) terms and KEGG pathways, respectively (Supplementary Table 5). As expected, the genes were unevenly distributed with an increase in density toward the ends of the pseudomolecules (Fig. 1a). We also identified genes encoding for 839 tRNA, 752 rRNA, 3,278 lncRNA (Fig. 1a, Supplementary Table 6, and Supplementary Data 1), and 1,644 transcription factors (TFs) from 36 families that accounted for 3.63% of the protein-coding genes (Supplementary Table 7).

### Comparative genomics and gene family evolution analysis

To assess candidate gene families involved in anthraquinone biosynthesis, we compared the *S. tora* genome with those from 15 related plant species. Reciprocal pairwise comparisons^39^ of the 16 species (15 legumes and grapevine) revealed that *S. tora* has the most species-specific genes of all the 16 plants compared, with 7,231 (15.9%) genes that are specific to *S. tora* (Fig. 1b, Supplementary Fig. 10, and Supplementary Table 8). We compared gene family expansion and contraction across the species to identify gene families that were expanded or contracted in *S. tora*. Of the 36,597 gene families found among the sixteen species, 2,874 and 3,371 gene families were expanded and contracted in *S. tora*, respectively (Supplementary Fig. 11). The gene families that were expanded in *S. tora* were enriched for several Gene Ontology (GO) and KEGG terms, including those involved in specialized metabolism including “phenylpropanoid biosynthesis,” “isoflavonoid biosynthesis,” and “terpene biosynthesis,” likely reflecting the importance of genes for the biosynthesis of phenolics, isoflavonoids, and terpenoids in *S. tora* (Fig. 1c and Supplementary Data 2). To investigate *S. tora* metabolism further, we developed a genome-scale metabolic network database of *S. tora* named StoraCyc and identified enriched metabolic pathways. Expanded gene families in *S. tora* were enriched in phenolic and nitrogen-containing specialized metabolism, cofactor, carbohydrate, and hormone metabolism of StoraCyc (Supplementary Fig. 12 and Supplementary Table 9). We also examined enriched metabolic domains in families that are expanded only in *S. tora*, rapidly expanded in *S. tora*, and rapidly expanded only in *S. tora*. Phenolic specialized metabolism was the only domain of metabolism enriched in all these families (Supplementary Fig. 12 and Supplementary Data 3).

We next probed which of the lineage-specifically expanded families might be involved in anthraquinone biosynthesis. In plants, type III polyketide synthases such as chalcone synthases (CHSs) are involved in the biosynthesis of plant specialized metabolites, particularly acetate-pathway-derived flavonoids, stilbenes, and aromatic polyphenols^33,40,41^. The *S. tora* CHS family contains twelve CHS (Supplementary Fig. 13) and sixteen CHS-L genes (Supplementary Fig. 13 and Supplementary Table 10). Interestingly, the CHS-L gene family specifically and rapidly expanded only in the *S. tora* genome (16 genes in *S. tora*, 5 in *C. fasciculata*, 2 in *A. hypogaea*, 1 in *M. truncatula*, 1 in *G. max*, and none in the other 11 species) (Fig. 1d and Supplementary Table 11). Twelve of the CHS-L genes are specific to the *S. tora* lineage and the majority of *S. tora* CHS-L genes (15 of 16) are distributed only in chromosome 7 and arranged in tandem (Fig. 1d). Interestingly, CHS genes contracted in the *S. tora* genome (Supplementary Table 11). Even though carminic acid (C-glucosylated anthraquinone) was produced in *Nicotiana* plants by combining an octaketide synthase gene from *Aloe arborescens*, two cyclases from *Streptomyces*, and a glycosyltransferase from an insect^42^, direct evidence of anthraquinone biosynthesis using plant CHS enzymes has not been established so far. However, several studies speculated the involvement of CHS-Ls in synthesizing anthraquinones^33,34,42^. At the genomic level, we found that the CHS-L gene family expanded most notably in *S. tora*, which may explain in part why *S. tora* is rich in anthraquinones.

### Metabolite profiling and transcriptomics of seed development

**Fig. 2:**
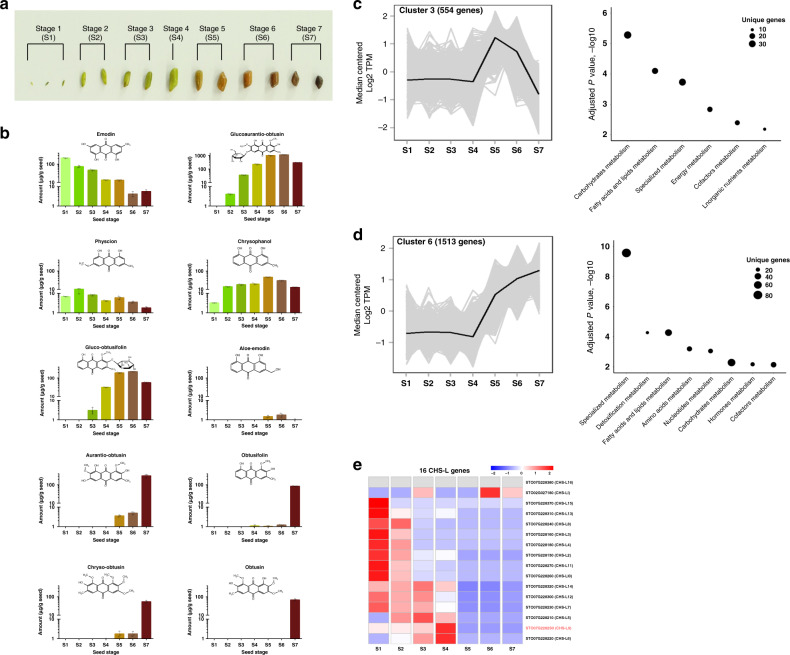
Analysis of anthraquinone contents and CHS-L gene expression during *S. tora* seed development. **a** Developmental progression of *S. tora* seeds (Stage 1–Stage 7). **b** Concentrations of ten anthraquinones during the seven developmental stages of *S*. *tora* seeds (mean ± SD, *n* = 3). Dots represent individual values. **c**, **d** Scaled transcript expression profiles (in transcripts per million, TPM) of cluster 3 (554 genes) and cluster 6 (1,513 genes) during seed development and enriched metabolic domains within these two clusters. **e** Expression analysis of CHS-L genes during seed development. Heatmap represents normalized transcripts per million (TPM) from two biological replicates. S1–S7 represents the seed-development stages of *S. tora*. The source data underlying Fig. 2b are provided as a Source Data file.

To test the hypothesis that CHS-Ls might be involved in anthraquinone biosynthesis in *S. tora*, we turned to the tissue that is enriched in anthraquinones, the seed. We profiled anthranoids from seven developmental stages of the seed (Fig. 2a), using ten standard anthraquinones (Supplementary Table 12) as references for quantification. Anthraquinone accumulation varied in each stage (Fig. 2b). Importantly, the profile shifted toward modified derivatives such as glucoaurantio-obtusin, aurantio-obtusin, obtusifolin, and chryso-obtusin during late stages of seed development (Fig. 2b and Supplementary Table 13) essentially becoming major storage metabolites in dry seeds.

To identify genes involved in the biosynthesis of anthraquinones during seed development, we performed transcriptome and metabolome analysis from developing seeds. The majority (68%) of genes decreased in expression during seed maturation (Supplementary Fig. 14 and Supplementary Data 9). Similarly, metabolic gene expression decreased across all metabolic domains during seed maturation (Supplementary Fig. 15 and Supplementary Data 4), consistent with metabolite-profiling results, which showed that the majority of primary metabolites involved in central carbon metabolism were reduced after stage 4 (Supplementary Figs. 16, 17, and Supplementary Data 5, 6). However, some genes increased in expression during seed maturation (32% genes represented by 5 clusters, Supplementary Fig. 14 and Supplementary Data 9), which we reasoned would be enriched in anthraquinone biosynthetic enzymes. To identify genes that showed similar expression patterns as anthraquinone biosynthesis, we first identified all genes that were differentially expressed relative to stage 1 during seed development. Co-expression analysis of differentially expressed genes during seed development detected nine co-expression clusters (Supplementary Fig. 14). Among them, clusters 3 and 6 showed similar patterns to anthraquinone accumulation in which genes were highly induced starting stage 5 (Fig. 2c, d). Cluster 6 was statistically overrepresented with genes annotated as transferases, UDP-glycosyltransferases, and oxidoreductases, which may reflect enzymes involved in the tailoring of anthraquinones to produce gluco-obtusifolin, glucoaurantio-obtusin, and other derivatives including aurantio-obtusin (Fig. 2b and Supplementary Table 14). In addition, genes in clusters 3 and 6 were enriched with specialized, fatty acid and lipid, cofactor, and carbohydrate metabolism in StoraCyc (Fig. 2c, d, and Supplementary Data 7).

### Identification of a candidate anthraquinone synthase family

With these data in hand, we searched specifically for CHS-L genes that were induced in stage 4 when the primary metabolite levels decrease and anthraquinones start to accumulate. Among the 16 CHS-L genes, two genes (STO07G228250 (CHS-L9) and STO07G228220 (CHS-L6)) showed high expression levels at stage 4 (Fig. 2e and Supplementary Table 15), where anthraquinone contents started to accumulate in seeds (Supplementary Table 13). Both of these genes share high amino acid sequence similarities with each other and with STO02G027180, the gene that was highly expressed at stage 6 of seed development (Fig. 2e). Further comparison of sequence alignment and phylogenetic tree analysis with previously characterized octaketide synthases, HpPKS and ArOKS, which were presumed to be involved in hypericin and barbaloin biosynthesis based on the production of the octaketide shunt products^35,37^, showed that STO07G228250 (CHS-L9) was more similar to them than the other two CHS-Ls (Supplementary Fig. 18). Therefore, we hypothesized that STO07G228250 (CHS-L9) could be engaged in anthraquinone biosynthesis in *S. tora*. As a control, we also selected a member of the CHS family, STO03G058250 (Supplementary Fig. 18), presumed to be involved in flavonoid biosynthesis, for biochemistry experiments.

### Biochemical confirmation of an anthraquinone enzyme class

**Fig. 3:**
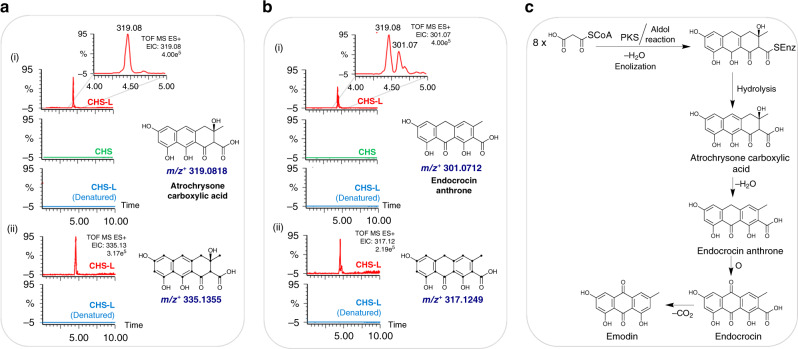
Enzyme assays and MS analysis of anthraquinones. **a** (i) Extracted ion chromatograms (EIC) for the compound with the mass 319.08 Da in reaction mixtures containing malonyl-CoA as substrate and chalcone synthase-like (CHS-L9) (STO07G228250), chalcone synthase (CHS) (STO03G058250), or heat-denatured CHS-L9. (ii) EIC for the mass 335.13 Da in reaction mixtures containing ^13^C_3_-malonyl-CoA containing CHS-L9 or heat-denatured CHS-L9 enzyme. Dots in the structure represent ^13^C-labeled carbons. **b** (i) EIC for the mass 301.07 Da in reaction mixtures containing malonyl-CoA as substrate containing CHS-L9, CHS, or heat-denatured CHS-L9. The inset shows a zoomed region of the EIC chromatogram. (ii) EIC for the mass 317.12 Da in reaction mixtures containing ^13^C_3_-malonyl-CoA containing CHS-L9 or heat-denatured CHS-L9 enzyme. **c** PKS-mediated biosynthetic pathway of anthraquinones. Two pathway intermediates, atrochrysone carboxylic acid and endocrocin anthrone, were produced in the CHS-L9-catalyzed reaction mixture.

To perform enzymatic assays, we expressed STO07G228250 (CHS-L9) and STO03G058250 (CHS) heterologously in *E. coli* and purified them to homogeneity (Supplementary Fig. 19). Enzyme assays were conducted in a phosphate buffer saline containing malonyl-CoA for successive condensation reactions to produce polyketides. STO07G228250 (CHS-L9)-catalyzed reaction mixture revealed the existence of two molecules with a molecular mass of 319.08 Da and 301.07 Da (Fig. 3). Neither of these metabolites was detected in reactions containing STO03G058250 (CHS) or heat-denatured STO07G228250 (CHS-L9) (control), indicating that these two masses are most likely the products of the PKS-catalyzed reaction (Fig. 3). The ESI-MS spectrum showed a compound with a distinct peak at *m/z*^+^ 319.0827 (retention time (*t*_R_) 4.45 min), which corresponds exactly to the mass of atrochrysone carboxylic acid (C_16_H_14_O_7_ with 319.0818 Da in the proton-adduct mode). Furthermore, the theoretical isotope model for the same chemical formula corroborated perfectly to the observed isotope mass (Supplementary Fig. 20). Likewise, the ESI-MS spectrum of the latter metabolite (*t*_R_ 4.62 min) *m/z*^+^ 301.0715 matched to the mass of endocrocin anthrone (C_16_H_12_O_6_ with calculated exact mass of 301.0712 Da) for which the theoretical isotope mass model and observed mass isotope were perfectly aligned (Supplementary Fig. 20). To further verify these metabolites as PKS-derived products, a set of reactions were conducted with STO07G228250 (CHS-L9) and heat-denatured STO07G228250 (dead CHS-L9) containing ^13^C_3_-malonyl-CoA as substrate. The EIC for all carbon-labeled atrochrysone carboxylic acid (^13^C_16_H_14_O_7_, exact mass: 335.1355 Da) (Fig. 3 and Supplementary Fig. 20) and endocrocin anthrone (^13^C_16_H_12_O_6_, exact mass: 317.1249 Da) (Fig. 3 and Supplementary Fig. 20) was confirmed to be present in only the CHS-L9 catalyzed reaction. The observed ESI-MS spectra aligned to the theoretical isotope mass of the corresponding metabolites. Except for these two metabolites, none of the other octaketide metabolites, such as emodin anthrone, endocrocin, emodin, chrysophanol, or islandicin, was produced even when NADPH was added in the reaction mixtures.

Previous studies reported the production of derailment products (SEK4 and SEK4b) by plant octaketide synthases such as HpPKS2 and ArOKS (Supplementary Fig. 1)^33,34^. We performed TOF-ESI-MS^2^ analysis for both of the precursor ions 319 and 301 and compared them to mass fragments of emodin and aloe-emodin (Supplementary Figs. 21 and 22), which share similar anthranoid scaffold and are known to be derived from atrochrysone carboxylic acid and endocrocin anthrone (Supplementary Fig. 1). The ESI-MS^2^ fragments of the precursor ions 319 (Supplementary Figs. 23 and 24) and 301 (Supplementary Figs. 25 and 26) shared most of the fragments with the emodin and aloe-emodin. However, TOF-ESI-MS^2^ sister fragments of SEK4 and SEK4b reported previously^34^ did not align to any of the fragments of standard anthraquinones, nor to atrochrysone carboxylic acid and endocrocin anthrone produced in the reactions. These evidences indicate that the metabolites produced in the reaction mixture are anthranoid scaffolds, not the octaketide shunt products. Altogether, these results indicate that STO07G228250 (CHS-L9), a type III PKS, carries out the first committed step of anthraquinone biosynthesis via polyketide pathway in plants. We could not detect a final product such as emodin or other fully oxidized products in the reaction mixture. The complete biosynthesis might need additional enzymes for decarboxylation and oxidation. It is possible that other CHS-L genes might also participate in anthraquinone biosynthesis. Unlike in plants, anthraquinones are produced via type II PKS enzymes in bacteria such as *Streptomyces*^43–45^, *Photorhabdus luminescens*^46,47^, and *Verrucosispora*^48^ and type I PKS enzymes in fungi^18,23^. Thus, the biosynthetic route of anthraquinones is distinct in plants and other anthraquinone-producing organisms, illustrating a convergent metabolic evolution.

In summary, the reference genome of *S. tora* revealed the rapid evolution of putative polyketide synthase genes. By combining metabolomics, transcriptomics, and biochemical characterization of a candidate polyketide synthase, we discovered the anthranoid-forming enzyme in plants. With these tools in hand, elucidation of genes involved in the rest of the anthraquinone biosynthesis pathway in *S. tora* and other species will be accelerated. These resources can also be used as a platform to develop a medicinally useful cultivar of *S. tora* with a high content of bioactive molecules.

## Methods

### DNA sequencing

We sequenced a cultivated diploid *Senna tora* cv. Myeongyun (voucher number: IT89788) grown in Jeonju, Korea (N: 35° 49′; E: 127° 09′). The total DNA was extracted from young fresh leaves of *S*. *tora* cv. Myeongyun using the modified cetyltrimethylammonium bromide (CTAB) method^49^. DNA purity and concentration were checked by electrophoresis analysis on 1.2% agarose gel and by DropSense96 Spectrophotometer (Trinean, Belgium). A total of 34 single-molecule real-time (SMRT) cells were run on the PacBio RS II system and 5 cells on the Sequel system using P6/C4 chemistry. We generated a total of 80.01 Gb of clean reads (Supplementary Table 1).

Illumina sequencing libraries were prepared according to the Illumina protocols. Briefly, 1 μg of genomic DNA was fragmented by Covaris. The fragmented DNA was repaired, and the base adenine was ligated to the 3′ end. Illumina adapters were then ligated to the fragments, and the proper samples were selected. The size-selected product was PCR-amplified, and the final product was validated using the Agilent Bioanalyzer. Then we sequenced 200-bp paired-end (PE) and 3–20- kb mate-pair (MP) libraries and 500-bp PE using the HiSeq™ 2500 and MiSeq platforms (Illumina, San Diego, USA), respectively. Finally, we generated a total of 577.93 Gb of clean reads for the 200- and 500-bp PE and 3-, 5-, 10-, and 20-kb MP libraries (Supplementary Table 1).

### Genome-size estimation

Total Illumina DNA sequences were subjected to preprocessing steps, which included adapter trimming, quality trimming (Q20), and contamination removal. Adapter and quality trimming were conducted using Trimmomatic v0.36 (ref. ^50^), and *S. tora* organellar genome contamination of each sample was removed by CLCMapper v4.2.0 (https://www.qiagenbioinformatics.com/products/clc-assembly-cell/) using the chloroplast genome (Genbank ID: NC_030193) and mitochondria genome sequences (Genbank ID: NC_038053)^51^. All preprocessed sequences were subjected to genome-size estimation using the *k*-mer-based method^52^. The *k*-mer frequencies (*k*-mer size = 21) obtained using the Jellyfish v2.0 method^53^, and the genome size was calculated by using the following formulas: (1) genome-coverage depth = (*k*-mer coverage depth × average read length)/(average read length – *k*-mer size + 1), and (2) genome size = total base number/genome-coverage depth. A total of 27.5 Gb of clean Illumina reads from the 200-bp PE library were used to determine the genome size of *S. tora*. In this study, the distribution of 21 *k*-mer showed a major peak at 50×. According to the total number of *k*-mers and the corresponding *k*-mer depth, the *S. tora* genome size was estimated to be ~547.02 Mb (Supplementary Fig. 2).

### Genome assembly

High-quality PE and MP sequences (Phred score >20) were obtained by removing low-quality sequences and duplicated reads from whole-genome NGS data. Three de novo assemblers, SOAPdenovo v2.04 (ref. ^54^), Allpaths-LG v48777 (refs. ^55,56^), and Platanus v1.2.1 (ref. ^57^), were performed using default parameters. For scaffolding of contig sequences, mate-pair (MP) reads were mapped to contig sequences and scaffold sequences were generated using SSPACE v3.0 with default parameters. To validate scaffold sequences, MP reads were remapped to the scaffold sequences and misscaffold sequences were disassembled into initial contig sequences using an in-house script.

The average coverage of SMRT sequences was about 146× by using RS II and Sequel systems. An average subread length was about 9 kb and the maximum length was 104.5 kb. We removed the sequences of *S. tora* organellar genomes. Then, the filtered subread sequences were assembled de novo using the diploid assembly FALCON v0.4 assembler^58^. To increase the assembly accuracy, the length cut-off option was specified based on the subreads’ N50 value of 14 kb and contigs were further corrected with Sequel data by Arrow (https://github.com/PacificBiosciences/GenomicConsensus, v2.1.0). To improve the quality of genome assembly results, we also performed error correction using default parameters of BWA and GATK’s FastaAlternativeReferenceMaker^59^ with haplotig-merged primary contigs and 101.2× Illumina reads (PE_200bp).

To obtain the best possible draft sequence, we compared the results obtained by SOAPdenovo2, Allpaths-LG, Platanus, and FALCON algorithms. De novo assembly by Platanus and FALCON outperformed the results produced by SOAPdenovo2 and Allpaths-LG (Supplementary Table 2). The number of contigs was lower, N50 length was longer, the assembled size was close to the estimated genome size, and their contiguity statistics were higher. With the assembly obtained by Platanus and FALCON, we also assessed the quality of genome assembly using Benchmarking Universal Single Copy Orthologs (BUSCO)^38^. The percentage of complete proteins was 90.6% for the Platanus assembly and 94.3% for the FALCON assembly (Supplementary Table 3). Based on these criteria, the assembly developed using FALCON assembler was chosen for the genome annotation. We evaluated the quality of the assembly by mapping the Illumina reads back to the scaffolds (99.7%) and expressed sequence tag (EST) sequences mapping to the scaffolds (97.2% of Iso-Seq and 89.9% of RNA-Seq) (Supplementary Table 16), supporting the high quality of the *S. tora* genome assembly.

### Physical map validation with BAC libraries

To validate the assembled genome against a physical map, we generated bacterial artificial chromosome (BAC) libraries. First, 15 g of young fresh leaves were harvested from growth-room-grown *S. tora* cv. Myeongyoun plants that have been placed in the dark for 48 h to reduce carbohydrate concentration, which may cause carryover contamination and be detrimental to subsequent enzyme reactions. Fresh leaf tissues were ground to a fine powder in liquid nitrogen using a pestle and mortar. Leaf tissues were transferred immediately to an ice-cold lysis buffer and gently stirred to extract nuclei. The nuclei were embedded in agarose plugs and transferred to proteinase K buffer to obtain high-molecular-weight (HMW) DNA. The HMW DNA was partially digested using *Hin*dIII- and *Bam*HI-restriction enzymes and underwent a size selection three times in order to obtain consistently large inserts. Size-selected DNA was ligated with pSMART BAC vector and transformed into DH10B-competent cells. The *Hin*dIII BAC library has an average insert size of 95 kb and a titer of 1.6 × 10^6^. Certified BAC clones were colonized on agar medium and cultured in liquid medium supplemented with chloramphenicol.

The ten BAC clones were completely sequenced using 454 Life Sciences GS FLX System (GS FLX) and ABI 3730×l DNA Analyzer. Analyzed sequencing data were assembled using Newbler v2.8 (https://www.ncbi.nlm.nih.gov/assembly/GCA_000507345.1/) and used to create contigs or scaffolds. To fill the gap of sequences, we used primer walking^60^. The primer walking method has been widely used in genome sequencing projects to determine the order of contigs and connect the remaining sequence gaps between the contigs. This way, a draft sequence for ten BAC clones was created. Finally, completed BAC clone sequences were checked by using the HiSeq sequence data for sequence error correction. To validate the genome assembly obtained by FALCON, we performed an all-by-all alignment (-minIdentity = 80–99, -minScore = 100, -fastMap) of the 10 complete BACs and the assemblies using BLAT v3.2.4 (ref. ^61^).

### Genotype-by-sequencing linkage analysis

Genomic DNA was extracted from the two parents (*S. tora* cv. Myeongyun (voucher number: IT89788) and ST-9 (voucher number: IT104602)) and 153 F2 progeny using a Qiagen plant DNAeasy kit. Two genotype-by-sequencing (GBS) libraries were prepared using *Ape*KI restriction enzyme as described in Elshire et al.^62^. The GBS libraries (74 F2 individuals + two parents; 79 F2 individuals + two parents) were sequenced on an Illumina HiSeq2500 system. Low-quality bases and adapter sequences were trimmed using Trimmomatic v0.36 (ref. ^50^) and the trimmed reads from each sample were mapped to the *S. tora* draft assembly using BWA-MEM^63^. HaplotypeCaller in GATK^59^ was used to call single-nucleotide polymorphisms (SNPs) and generate a raw vcf file. High-quality biallelic SNPs were selected using VCFtools^64^ with the following conditions: (1) minimum read depth ≥ 5, (2) minimum genotype quality ≥ 20, and (3) missing genotype ≤ 30%. The SNP positions that showed polymorphic homozygous SNPs between the parents were retained for linkage analysis. Linkage analysis was conducted using QTL IciMapping v4.1 (ref. ^65^) with the Kosambi function.

A total of 721.8 million raw PE reads were generated from two *Apek*I GBS libraries, and 372 million trimmed PE reads were used for subsequent linkage analysis. Of those, about 89.8% reads were mapped to the *S. tora* reference assembly and 88.6% (329.5 million reads) were concordantly mapped, which was representing about 2.1 million properly mapped PE reads per sample. The GATK HaplotypeCaller called 289,768 and 4.78 million unfiltered variants from libraries 1 and 2, respectively. After low-quality SNPs were filtered, 7,584 and 15,604 high-quality SNPs were obtained from libraries 1 and 2, respectively, and 5,071 markers were commonly represented in both. Three genetics maps independently constructed with three sets of SNP markers (from libraries 1 and 2 and common) were evaluated in terms of a number of anchored contigs and genome representation, and the map that used common markers between libraries 1 and 2 was selected for further analysis. This map contained 2,654 nonredundant markers representing 3,587 cM within 12 linkage groups (LG13 contained only one marker). With this linkage map, we tried to regroup markers by increasing group number parameters from 13 to 25; however, the efforts were not successful to make the 13th linkage group. Finally, the linkage map was compared to pseudochromosomes constructed by Hi-C and we were able to split LG5 into two groups: one with three contigs (164 markers covering 34.4 Mb) and the other with 8 contigs (263 markers covering about 44 Mb). The final *S. tora* genetic map with Hi-C information resulted in 13 linkage groups with 4,455 markers spanning 2,780 cM of genetic distance (Supplementary Data 8). It enabled to anchor 111 contigs (contig 3 split into two contigs: c31-1 and c31-2) to 13 linkage groups, which represented about 401 Mb of *S. tora* sequence assembly (Supplementary Table 17). Genetically, LG8 was the longest linkage group (347.5 cM) followed by LG13 (343.8 cM) and LG5 (313 cM). Whereas, 487 markers anchored about 45 Mb of sequences in LG5, which was the longest anchored chromosome. Physical distance per genetic distance was calculated as 144 kb/cM on average across the whole genome (Supplementary Fig. 7).

### Hi-C technology-assisted pseudochromosome construction

To generate pseudochromosomes, chromatin-conformation capture (Hi-C) data were generated using a Phase Genomics (Seattle, WA) Proximo Hi-C Plant Kit, which is a commercially available version of the Hi-C protocol^66^. Intact cells from two samples were cross-linked using a formaldehyde solution, digested using the *Sau*3AI restriction enzyme, and proximity-ligated with biotinylated nucleotides to create chimeric molecules composed of fragments from different regions of the genome that were physically proximal in vivo, but not necessarily genomically proximal. Continuing with the manufacturer’s protocol, molecules were pulled down with streptavidin beads and processed into an Illumina-compatible sequencing library. Sequencing was performed on an Illumina NextSeq 500 (Illumina, San Diego, USA), generating a total of 188,501,285 PE read pairs.

Reads were aligned to the reference assembly following the manufacturer’s recommendations (https://phasegenomics.hithub.io/2019/09/19/hic-alignment-and-qc.html). Briefly, reads were aligned using BWA-MEM with the −5SP and -t 8 options specified, and all other options as default. SAMBLASTER^67^ was used to flag PCR duplicates, which were later excluded from further analysis. Alignments were then filtered with samtools^68^ using the -F 2304 filtering flag to remove non-primary and secondary alignments.

Phase Genomics’ Proximo Hi-C genome scaffolding platform was used to create chromosome-scale scaffolds from the corrected assembly^69^. Similar to the LACHESIS method^70^, this process computes a contact frequency matrix from the aligned Hi-C read pairs, normalized by the number of *Sau*3AI restriction sites (GATC) on each contig, and constructs scaffolds in such a way as to optimize the expected contact frequency and other statistical patterns in Hi-C data. Approximately 20,000 separate Proximo runs were performed to optimize the number of scaffolds and scaffold construction in order to make the scaffolds as concordant with the observed Hi-C data as possible. The Hi-C sequences were aligned to the draft contig assemblies. Finally, Juicebox^71,72^ was used to correct scaffolding errors as well as to introduce two new breaks into two putative misjoined contigs (contigs 3 and 110). All contig sequences not anchored to chromosomes were constructed with 100 N’s as a linker following the order of contig sizes designated chromosome 00 (Chr 00). The length and number of contigs for each chromosome are shown in Supplementary Table 18.

### Repetitive-element analysis

Initially, repeat regions were predicted using the de novo method and classified into repeat subclasses. De novo repeat prediction for *S. tora* was conducted using RepeatModeler (http://www.repeatmasker.org/RepeatModeler/), which includes other methods such as RECON^73^, RepeatScout^74^, and TRF^75^. Furthermore, the repeats were masked using RepeatMasker v4.0.5 (http://www.repeatmasker.org/) with RMBlastn v2.2.27+ and classified into its subclasses with the reference of Repbase^76^ v20.08 databases (https://www.girinst.org/repbase/).

Transposable elements are major components of plant genomes, but they have not been examined in *S. tora*. The *S. tora* genome masked 53.9% of the assembly as repeat sequences. Long terminal repeat (LTR) retrotransposons, mainly Gypsy-type LTRs, are the most abundant, occupying 15.6% of the genome (Supplementary Table 19). The fraction of repeat sequences in the genome is very similar to other Leguminosae family plants such as pigeon pea (51.6%)^77^, mung bean (50.1%)^78^, and chickpea (49.4%)^79^.

### Genome annotation

The genes from the *S. tora* reference genome were predicted using an in-house gene prediction pipeline, which includes three modules: evidence-based gene modeler, ab initio gene modeler, and consensus gene modeler. To improve the accuracy of gene prediction, we downloaded a total of 118,390 Iso-Seq reads in GenBank SRA database (SRP159435)^15^. RNA-Seq from five tissues (leaf, root, stem, flower, and dry seed) and Iso-Seq data were aligned against the *S. tora* genome. Initially, the sequenced transcriptomes were mapped to the *S. tora* repeat-masked reference genome using Tophat^80^, and transcripts/gene structural boundaries were predicted using Cufflink^80^ and PASA^81^. To train the ab initio gene modeler AUGUSTUS^82^ and evidence-based gene modeler GENEID^83^, we selected a few genomes using Exonerate^84^. Genomes we used are *Abrus precatorius, Arachis hypogaea, Arachis duranensis, Arachis ipaensis, Cajanus cajan, Cicer arietinum, Faidherbia albida, Glycine max, Glycine soja, Lablab purpureus, Lupinus angustifolius, Medicago truncatula, Mucuna pruriens, Phaseolus vulgaris, Prosopis alba, Sclerocarya birrea, Trifolium medium, Trifolium subterraneum, Vigna angularis, Vigna radiata, Vigna subterranea, Vigna unguiculata,* and *Arabidopsis thaliana*. Finally, the predicted ab initio gene models, transcript models, and evidence-based gene models were subjected to build consensus gene models. The consensus genes were subjected to functional annotations from biological databases (NCBI-NR databases, SwissProt, Gene Ontology, and KEGG pathways) by using the Blast2GO^85^. The transcription factor genes were predicted through searching DNA-binding domains using InterProScan v5.36–75.0 (ref. ^86^) and the family name assigned through the rules given in PlantTFDB (v5.0, http://planttfdb.cbi.pku.edu.cn/). The gene models were supported by 97.2% Iso-Seq data, which comprised 118,390 high-quality isoforms derived from leaf, root, and two different developmental stages of seeds, and 89.9% RNA-Seq data derived from seed, leaf, root, stem, flower, and seven different stages of seeds, suggesting that the assembly includes most of the *S. tora* gene space (Supplementary Table 20).

### Identification of lncRNA

A pipeline for lncRNA identification was designed according to a previous study^87^. In brief, among the total transcripts obtained from reference-guided assembly of transcriptome data, transcripts with open-reading frame (ORF) for ≥100 amino acids and ≤200 nucleotides were removed. We also removed sequences with homology to protein sequences based on BLAST search against the SwissProt^88^ and Pfam^89^ protein databases. The coding potential of the remaining sequences was calculated using Coding Potential Calculator (CPC)^90^ and transcripts with CPC score ≥ −1.0 were removed as CPC scores between −1 and 1 are “weak noncoding” or “weak coding”. From the remaining transcripts, housekeeping RNAs (tRNA, rRNA, snRNA, and snoRNA) were removed by comparing with RNACentral database^91^ sequences (cutoff *E* value of 1e–10), and those completely matching with *S. tora* reference protein-coding gene sequences were also removed. Finally, we only retained the longest isoform for each gene to obtain the final set of 3,278 lncRNAs (Supplementary Data 1).

### Phylogenetic tree construction and evolution-rate estimation

To understand the evolutionary patterns of the *S. tora* genome and gene families, we performed comparative genome analysis. We used 14 legume species and one outlier (*Vitis vinifera*). The OrthoMCL v2.0.9 (ref. ^92^) method was used to find orthologous groups in the given genomes. The orthologous clusters were obtained by the Markov graph clustering (MCL v14–137) algorithm, through all-vs-all sequence similarity search by BLASTP v2.2.29+ with an *E*-value cutoff of 1e–3. The orthologous clusters that contain proteins from all 16 species were subjected to multiple-sequence alignment with MAFFT v7.305b^93^ and the alignments were corrected with Gblocks v0.91b^94^. The phylogenetic tree was reconstructed using IQ-Tree v1.5.0-beta^95^, using a maximum likelihood method with 1,000 bootstrap iterations. Here, the longest protein in each genome was selected among the proteins in each orthologous cluster. From the trees, the gene pattern changes such as contraction and expansion were observed among the genomes using CAFE v3.1 method^96^. Rapid expansion/contraction is indicated by statistically significant and non-random expansion/contraction at *P* < 0.01, as described in CAFE^96^. The evolutionary divergence timescale of the species was obtained from the clock and Yule model with the JTT substitution model (the gamma category count set to 4), which was implemented in BEAST2 method^97^. The calibration priors were set as 58–70 MYA for the common ancestor of *S. tora, C. fasciculata, M. pudica, and M. truncatula* and 105–115 MYA for the root according to the TimeTree database (http://timetree.org).

### Ks analysis

To calculate the synonymous-substitution Ks values, we selected the orthologous gene pairs between species and the paralogous pairs within a species from the orthology analysis. The selected proteins were further subjected to multiple-sequence alignment with MAFFT v7.305b^93^ and corrected with Gblocks v0.91b^94^. The corresponding genomic regions of conserved proteins, which were observed from the corrected multiple alignments, were subjected to Ks calculation using ParaAT v2.0 (ref. ^98^) with the Yang–Nielsen approach implemented in PAML^99^. The Ks distribution plot (Supplementary Fig. 27) was drawn using in-house Python and R scripts.

Ancient whole-genome duplication (WGD), also known as paleopolyploidization events, is shared throughout angiosperm history^100^ and represents a powerful evolutionary force for diversification, neofunctionalization, and innovation^101–103^. We did not detect the peak of the recent WGD found in soybean, suggesting that Caesalpinoideae, including *S. tora* and *Mimosa pudica,* do not have the soybean-specific WGD event (Supplementary Fig. 27)^104^. Homology analysis with 6,310 orthologous genes shared by *S. tora* and 15 other green plant species was used to construct a phylogenetic tree based on a concatenated sequence alignment using MAFFT v7.305b. In this phylogenetic tree, *S. tora*, as expected, clustered with other legume crops, although the evolutionary distance from *S. tora* to Papilionoideae such as soybean, *Medicago truncatula*, and chickpea was relatively large (Supplementary Fig. 11). The phylogenetic tree confirmed the grouping of Caesalpinioideae species such as *S. tora* and *M. pudica*. The first divergence between Caesalpinioideae and Papilionoideae was estimated at approximately 81.9–93.6 MYA (Supplementary Fig. 11). Furthermore, *Senna* and *Chamaecrista* genera diverged from the Mimosoid clade (*Faidherbia albida* and *Mimosa pudica*) ~59.4–66.5 MYA^105^ (Supplementary Fig. 11).

### Enzyme prediction

*S. tora* enzymes and metabolic pathways were predicted using the Plant Metabolic Network (PMN)’s pipelines^106^. This process starts by annotating amino acid sequences with the Ensemble Enzyme Prediction Pipeline (E2P2) version 4.0 to identify enzymes and assign reactions they may catalyze based on a database of known enzymes called Reference Protein Sequence Database (RPSD) version 4.2. Metabolic pathways were predicted using PathoLogic software, which is part of the Pathway Tools v23.5 package from SRI^107^. The predicted pathways were further refined using PMN’s Semi-Automated Validation Infrastructure (SAVI) software version 3.1 by applying pathway assignment criteria based on manual curations. In total, 6,159 enzymes and 442 metabolic pathways were predicted in *S. tora* into a database called StoraCyc, available online at https://plantcyc.org/.

To identify enriched metabolic pathways among expanded or rapidly expanded gene families, we created the following four datasets: Dataset 1 included 15,921 genes from 2,874 families that were expanded or rapidly expanded in *S. tora*. Dataset 2 included 10,571 genes from 1,775 families that were exclusively expanded or rapidly expanded in *S. tora*. Dataset 3 included 7,382 genes from 411 families that were rapidly expanded in *S. tora*. Dataset 4 included 5,693 genes from 306 families that were exclusively rapidly expanded in *S. tora* (Supplementary Data3). The background used in this enrichment analysis was all genes in *S. tora*. P values of the enrichment analysis were calculated with a hypergeometric test using the phyper() function followed by multiple test correction using false-discovery rate (FDR) via *P*.adjust(), both functions from the stats package version 3.6.2 in R version 3.6.3. Significant enrichment was defined as an adjusted *P* value ≤ 0.01.

### Primary metabolite profiling

Metabolome analysis was performed with 21 samples of frozen seed powders (~50 mg each) collected from seven seed developmental stages using the Capillary Electrophoresis Time of Flight Mass Spectrometry (CE-TOF-MS). CE-TOF-MS was run in two modes for cationic and anionic metabolites at Human Metabolome Technologies (Yamagata, Japan). The samples were mixed with 500 μL of methanol containing internal standards (50 μM) and homogenized using a homogenizer (a cell-breakage machine with beads (MS-100R, TOMY Digital Biology, Tokyo, Japan)). Then, chloroform (500 μL) and Milli-Q water (200 μL) were added to the homogenates, mixed thoroughly, and centrifuged (2300 × *g*, 4 °C, 5 min). The water layer (200 μL) was filtrated twice through 5-kDa cut-off filter (Ultra-free MC-PLHCC, Human Metabolome Technologies, Yamagata, Japan) to remove macromolecules. The filtrate was centrifuged and resuspended in 50 μL of ultrapure water immediately before the measurement. Cationic metabolite levels were analyzed using a commercial fused silica capillary (H3305-1002, HMT; i.d. 50 μM × 80 cm) with a commercial cationic electrophoresis buffer (H3301–1001, HMT), or anionic electrophoresis buffer (H3301–1020, HMT) as the electrolyte. A commercial sheath liquid (H3301–1020, HMT) was delivered at a rate of 10 μL/min. Approximately 10 nL of sample solution was injected at a pressure of 50 mbar for 10 s, and applied capillary voltages were set at 27 kV (cation mode) and 30 kV (anion mode), respectively. For both cationic and anionic modes, the spectrometer was scanned from *m/z* 50 to 1,000.

Peaks detected in CE-TOF-MS were extracted using an automated integration software (MasterHands ver. 2.16.0.15 developed at Keio University) in order to obtain peak information including *m/z*, migration time (MT), and peak area. The peak detection limit was set at the signal-noise ratio (S/N) of 3. Signal peaks corresponding to isotopomers, adduct ions, and other product ions of known metabolites were excluded, and the remaining peaks were annotated with putative metabolites from the MasterHands database based on their MTs and *m/z* values. The tolerance range for the peak annotation was configured at ±0.5 min for MT and ±10 ppm for *m/z*. For the 178 peaks detected (Supplementary Data 5), the average relative area and standard deviations (S.D.) were calculated in the 7 developmental stages of *S. tora* seeds. Absolute quantification was performed for 110 metabolites, including glycolytic and TCA-cycle intermediates, amino acids, and nucleic acids. All the metabolite concentrations were calculated by normalizing the peak area of each metabolite with respect to the area of the internal standard (solution ID: H3304-1002, HMT, Inc.) and by using standard curves, which were obtained by single-point (100 μM) calibrations. Finally, we obtained absolute quantitative values for 69 out of 110 metabolites (Supplementary Data 6). The ratio of the average relative peak area and *P* value from Welch’s *t* tests were calculated between the two stages (stage 1 vs. other stages).

### Anthraquinone extraction and analysis

*S. tora* seeds were collected and sorted into seven different ripening stages (Stage 1–Stage 7) depending on their size, color, and hardness. Classified seeds were ground with a mortar and pestle using liquid nitrogen to a fine powder and freeze-dried. Powdered samples (20 mg) were extracted with 1 mL of methanol using sonication for 30 min at 60 °C. After extraction, samples were centrifuged at 500 × *g* for 3 min at 25 °C, and the supernatant was filtered with 0.2 μM Acrodisc MS syringe filters with PTFE membrane (Pall Corporation, Port Washington, NY, USA). The filtrate was completely dried by EvaT-0200 Total Concentration System equipped with EvaS-3600 N2 generator (Goojung engineering, Seoul, Korea), mixed with methanol, and filtered again with Acrodisc 0.2 μM MS syringe filter for liquid chromatography–mass spectrometry (LC–MS) analysis.

Quantitative analysis of anthraquinones was performed by a 3200 QTRAP mass spectrometer with a Turbo V ion source (AB Sciex, Ontario, CA, USA) coupled with a VANQUISH UHPLC system (Thermo Fisher Scientific, CA, USA) equipped with binary solvent manager, sample manager, column heater, and photodiode array detector. UHPLC was performed on a ZORBAX Eclipse Plus column (1.8 μM, 2.1 mm × 100 mm, Agilent Technology, CA, USA) and mobile phases consisted of 5 mM ammonium acetate in water (eluent A) and 100% acetonitrile (eluent B). The gradient conditions were as follows: 0–1 min, 10% B; 1–4.5 min, 10–30% B; 4.5–8 min, 30–50% B; 8–11 min, 50–100% B; 11–14 min, 100% B. The flow rate was 0.5 mL/min and two microliters of samples were injected. For detecting peaks from test samples, MS parameter in ESI-negative mode was used as follows: nebulizing gas, 50 psi; heating gas, 50 psi; curtain gas, 20 psi; desolvation temperature, 550 °C; ion-spray voltage floating, 4.5 kV. The data obtained from MRM mode were quantitated using MultiQuant 3.0.2 software (AB SCIEX).

### RNA sequencing and analysis

Total RNA was isolated from seven developmental stages of seeds (Stage 1–Stage 7) (Supplementary Table 20). RNA extraction and RNA-Seq library preparations were performed, and RNA-Seq libraries were sequenced on the Illumina NextSeq 500 (Illumina, San Diego, USA). First, low-quality bases (PHERD score (Q) < 20) and adaptor contamination were removed by Trimmomatic v0.36 using the parameters “ILLUMINACLIP:TruSeq3-SE:2:30:10 LEADING:3 SLIDINGWINDOW:4:15 MINLEN:36”^2^. After checking for quality scores and read lengths, RNA-Seq reads were mapped *to S. tora* genome using STAR-2.6.0a with default parameters^108^. Expectation Maximization (RSEM-1.3.1)^109^ method was used to obtain the expression value for each gene in the genome (Supplementary Data 9). The read counts estimated by RSEM were subjected to edgeR v3.22.5 (ref. ^110^) to obtain differential expression scores along with the statistical significance based on the FDR. Furthermore, we applied the standard filters, i.e., genes per million (TPM) ≥ 0.3, read counts ≥ 5, and log2-fold changes ≥ 1 or ≤ −1 to derive the final list of differentially expressed genes^111^. Finally, the expressed genes (i.e., TPM ≥ 0.3 and read count ≥ 5) were included to show the different expression patterns during seed development. An in-house R script was used to generate the heatmap.

To analyze gene expression patterns of metabolic genes during seed development, all expressed (TPM > 0) *S. tora* genes predicted to catalyze small-molecule metabolism in StoraCyc were mapped to StoraCyc’s metabolic domains (Supplementary Data 4) based on their associated reactions. A violin plot was generated using ggplot2 version 3.3.2 in R version 3.6.3 to show the expression patterns of genes belonging to metabolic domains during seed development (Supplementary Fig. 15 and Supplementary Data 4).

The genes determined as DEGs in at least one pair of comparisons were selected, resulting in a total of 13,488 genes. The mean across replicates with base 2 of logarithm was clustered by *k*-means (*k* = 9) using the standard function of *k*-means in R version 3.6.3 with default parameters. Core genes were identified using a cluster score and overlaid on the plot representing expression values with ggplot2 version 3.3.2 in R version 3.6.3.

To identify enriched StoraCyc metabolic domains in clusters 3 and 6, we compared the metabolic domain annotations of genes annotated to these clusters to those annotated to all genes in *S. tora*. P values of the enrichment analysis were calculated with a hypergeometric test using phyper() followed by multiple test correction using False Discovery Rate via *P*.adjust(), both functions from the stats package version 3.6.2 in R version 3.6.3. Significant enrichment was defined as an adjusted *P* value ≤ 0.01. To avoid biases introduced by datasets containing a small number of genes, we defined a minimum threshold of at least ten genes present in each metabolic domain per cluster in order for the domain to be considered significantly enriched. The bubble plot was generated using ggplot2 version 3.3.2 in R version 3.6.3.

### Heterologous protein expression and enzyme assays

STO07G228250 (1,173 bp) encoding CHS-L9 and STO03G058250 (1,173 bp) encoding CHS cDNAs were PCR-amplified using a pair of oligonucleotide primers (Supplementary Table 21). STO07G228250 and STO03G058250 were cloned in pET28a(+) vector. The *E. coli* BL21 (DE3) strain harboring correct pET28a(+)_STO07G228250 and pET28a(+)_STO03G058250 plasmids was used for protein production. Cultures were induced by 0.4 mM isopropyl-β-D-thiogalactopyranoside (GeneChem, Daejeon, Korea) to start the recombinant protein expression. After incubation at 20 °C for 20–24 h, the cells were harvested by centrifugation, washed twice with 100 mM phosphate buffer saline (pH 7.5) containing 10% glycerol, and disrupted by sonication. The homogenates were centrifuged at 13,475 × *g* for 30 min at 4 °C to isolate soluble proteins from insoluble cell debris. The supernatants were applied to a separate column containing 1 ml of His_6_ Ni-Superflow Resin (Takara, Japan), which was equilibrated with a buffer containing 100 mM phosphate buffer saline (pH 7.5), 500 mM NaCl, 5 mM imidazole, 1 mM dithiothreitol, and 10% glycerol. The His_6_-tagged recombinant proteins were then eluted with eight column volumes of the aforementioned buffer containing 50 mM of imidazole. The elution was repeated with the same buffer containing 250 mM of imidazole. The purity and molecular mass of the recombinant proteins were verified by 12% SDS-PAGE. The fractions containing the pure protein were then pooled and concentrated using Amicon Ultra 15 (Millipore, 30 K NMWL centrifugal filters). Protein concentrations were measured by the Bradford method using the Bradford reagent (Protein Assay Dc, Bio-Rad, Hercules, CA, USA) using bovine serum albumin as standard.

Enzyme assays for anthraquinone biosynthesis were carried out in 1 ml volume in a microcentrifuge tube containing 5 mM of malonyl-CoA (Sigma-Aldrich, St. Louis, USA), 10 mM of MgCl_2_, and 10 µg/ml of pure protein in 100 mM phosphate buffer saline (pH 7.5). An identical reaction mixture containing the same amount of heat-denatured protein served as a negative control. A separate reaction was carried out with the same reaction constituents with an additional 1 mM of NADPH as a cofactor. Similarly, assays were carried out with identical reaction components, except for malonyl-CoA, which was replaced with the same amount of ^13^C_3_-malonyl-CoA (Sigma-Aldrich, St. Louis, USA). All reaction mixtures were incubated at 30 °C for 6 h, and stopped by heating the reaction mixture at 85 °C for 3 min.

For the STO03G058250 (CHS) enzyme, separate sets of reactions were carried out in the presence of *p*-coumaroyl-CoA (PlantMetaChem, Giessen, Germany) as the starting substrate and malonyl-CoA and ^13^C_3_-malonyl-CoA as extender substrates. Each reaction mixture contained 2 mM of *p*-coumaroyl-CoA, 5 mM of extender substrates, 10 mM of MgCl_2_, and 10 µg/ml of pure protein in 100 mM phosphate buffer saline (pH 7.5). An identical reaction mixture without the starting substrate served as a negative control. Each reaction was performed in three biological replicates.

### Enzyme assay quantification

The quenched reaction mixtures were centrifuged at 13,475 × *g* for 30 min to separate denatured protein, filtered through 0.2-µm syringe filter, and subjected to reverse-phase ultrapressure liquid chromatography (RPUPLC) coupled with photodiode array (PDA) when necessary, followed by high-resolution time-of-flight electrospray ionization (HRTOF ESI-MS) analysis. All enzyme assays were performed in triplicates.

RPUPLC-PDA was performed with an RP-18 column (50-mm long, 2.1-mm internal diameter, and 1.7-µm particle size) in Acquity (Waters) with UPLC LG 500 nm PDA detector using water as aqueous solvent A and acetonitrile (Thermo Fisher Scientific Korea, Seoul, Korea) as organic solvent B at the flow rate of 0.3 mL/min for 12 min under the following conditions of solvent B (0–100%) for (0–7) min, 100% for (7–9.5) min, and 0% for (9.6–12) min. HRQTOF ESI-MS and ESI-MS^2^ were performed in Acquity SYNAPT G2-S mass spectrometer (Waters, Milford, MA, USA). The selected precursor ions were further subjected to TOF-ESI-MS^2^ analysis in positive ionization mode.

The CHS enzyme STO03G058250 was investigated for its possible involvement in flavonoid biosynthesis. The reaction of STO03G058250 with *p*-coumaroyl-CoA and malonyl-CoA generated naringenin chalcone along with bisnoryangonin, and *p*-coumaroyltriacetic acid lactone (CTAL) demonstrating its participation in flavonoid biosynthesis in *S. tora* (Supplementary Figs. 28 and 29). The pyrone ring containing metabolites, bisnoryangonin and CTAL, are the shunt products produced after two and three malonyl-CoA condensations, respectively^112^. None of the stilbene-type derivatives were produced in the reactions.

### Reporting summary

Further information on research design is available in the Nature Research Reporting Summary linked to this article.

## Supplementary information

Supplementary Information file

Peer Review

Reporting Summary

Description of Additional Supplementary Files

Supplementary Data 1

Supplementary Data 2

Supplementary Data 3

Supplementary Data 4

Supplementary Data 5

Supplementary Data 6

Supplementary Data 7

Supplementary Data 8

Supplementary Data 9

## Data Availability

Data supporting the findings of this work are available within the paper and its Supplementary Information files. A reporting summary for this article is available as a Supplementary Information file. The datasets and plant materials generated and analyzed during the current study are available from the corresponding author upon request. Genome sequence reads, transcriptome sequence reads, Hi-C sequence reads, GBS sequence reads, and BAC library sequence data are deposited in GenBank under project number PRJNA605066. The genome assemblies and annotation files are deposited in GenBank under accession number JAAIUW000000000. The *S. tora* genome is also available at http://nabic.rda.go.kr/Species/Senna_tora2. Source data are provided with this paper.

## Source data

Source Data Chem Draw Files

## Code availability

In-house Python and R scripts for gene prediction, Ks distribution plot, and heatmap analyses can be freely downloaded at GitHub [https://github.com/MyungheeJung/Senna_tora.git].
