# Author Correction: Genome-enabled discovery of anthraquinone biosynthesis in *Senna tora*

**DOI:** 10.1038/s41467-021-21987-7

**Published:** 2021-03-08

**Authors:** Sang-Ho Kang, Ramesh Prasad Pandey, Chang-Muk Lee, Joon-Soo Sim, Jin-Tae Jeong, Beom-Soon Choi, Myunghee Jung, Daniel Ginzburg, Kangmei Zhao, So Youn Won, Tae-Jin Oh, Yeisoo Yu, Nam-Hoon Kim, Ok Ran Lee, Tae-Ho Lee, Puspalata Bashyal, Tae-Su Kim, Woo-Haeng Lee, Charles Hawkins, Chang-Kug Kim, Jung Sun Kim, Byoung Ohg Ahn, Seung Yon Rhee, Jae Kyung Sohng

**Affiliations:** 1 Genomics Division, National Institute of Agricultural Sciences, RDA, Jeonju, 54874 Republic of Korea; 2 Department of Pharmaceutical Engineering and Biotechnology, Sun Moon University, Asan, 31460 Republic of Korea; 3 Metabolic Engineering Division, National Institute of Agricultural Sciences, RDA, Jeonju, 54874 Republic of Korea; 4 Department of Herbal Crop Research, National Institute of Horticultural and Herbal Science, RDA, Eumseong, 55365 Republic of Korea; 5 Phyzen Genomics Institute, Seongnam, 13488 Republic of Korea; 6 Department of Forest Science, College of Agriculture and Life Science, Seoul National University, Seoul, 08826 Republic of Korea; 7 Department of Plant Biology, Carnegie Institution for Science, Stanford, CA 94305 USA; 8 Department of Applied Plant Science, College of Agriculture and Life Science, Chonnam National University, Gwangju, 61186 Republic of Korea; 9 Present Address: Department of Biological Engineering, Massachusetts Institute of Technology, Cambridge, MA 02139 USA; 10 Present Address: DNACARE Co. Ltd, Seoul, 06730 Republic of Korea

**Keywords:** Genomics, Plant evolution, Secondary metabolism

Correction to: *Nature Communications* 10.1038/s41467-020-19681-1, published online 18 November 2020.

In the original version of this Article, two sentences were incorrectly stated. First, in the last sentence of the second paragraph of the Introduction, “Bacteria, fungi and insects make anthraquinones via a polyketide pathway using complex polyketide synthase enzymes” should read “Bacteria, fungi and insects make anthraquinones via a polyketide pathway using type I or II polyketide synthases”. Second, in the second to last paragraph of the Results and discussion section, the sentence “Unlike in plants, anthraquinones are produced via type II PKS enzymes in bacteria such as *Streptomyces*^43-45^, *Photorhabdus luminescens*^46,47^ and *Verrucosispora*”^48^ should read “Unlike in plants, anthraquinones are produced via type II PKS enzymes in bacteria such as *Streptomyces*^43-45^, *Photorhabdus luminescens*^46,47^ and *Verrucosispora*^48^, and type I PKS enzymes in fungi”^18,23^.

In addition, two Supplementary Data files were incorrectly cited and one Supplementary Data file was omitted to cite in the Results and discussion section. First, in the second paragraph of the section “Metabolite profiling and transcriptomics of seed development” the Supplementary Data file supporting the statement “The majority (68%) of genes decreased in expression during seed maturation” was incorrectly cited as Supplementary Data 4. It should be Supplementary Data 9. Second, in the same section and paragraph the Supplementary Data file supporting the statement “Similarly, metabolic gene expression decreased across all metabolic domains during seed maturation (Supplementary Fig. 15)” was omitted to cite. The sentence should read “Similarly, metabolic gene expression decreased across all metabolic domains during seed maturation (Supplementary Fig. 15 and Supplementary Data 4)”. Third, in the same section and paragraph the Supplementary Data file supporting the statement “However, some genes increased in expression during seed maturation” was incorrectly cited as Supplementary Data 7. It should be Supplementary Data 9. These have now been corrected in both the PDF and HTML versions of the Article.

Furthermore, the original HTML version of this Article was updated shortly after publication because the previous HTML version linked to an incorrect Source Data file.

