# Supplementary material for: Genome-enabled discovery of anthraquinone biosynthesis in Senna tora
Source: Nat Commun. 2020 Nov 18;11:5875. doi: 10.1038/s41467-020-19681-1 (PMC7674472; doi:10.1038/s41467-020-19681-1)
Supplement: Supplementary file 1 — Supplementary Information file [file 41467_2020_19681_MOESM1_ESM.pdf]

Genome-enabled discovery of anthraquinone biosynthesis in *Senna tora*

Kang *et al*

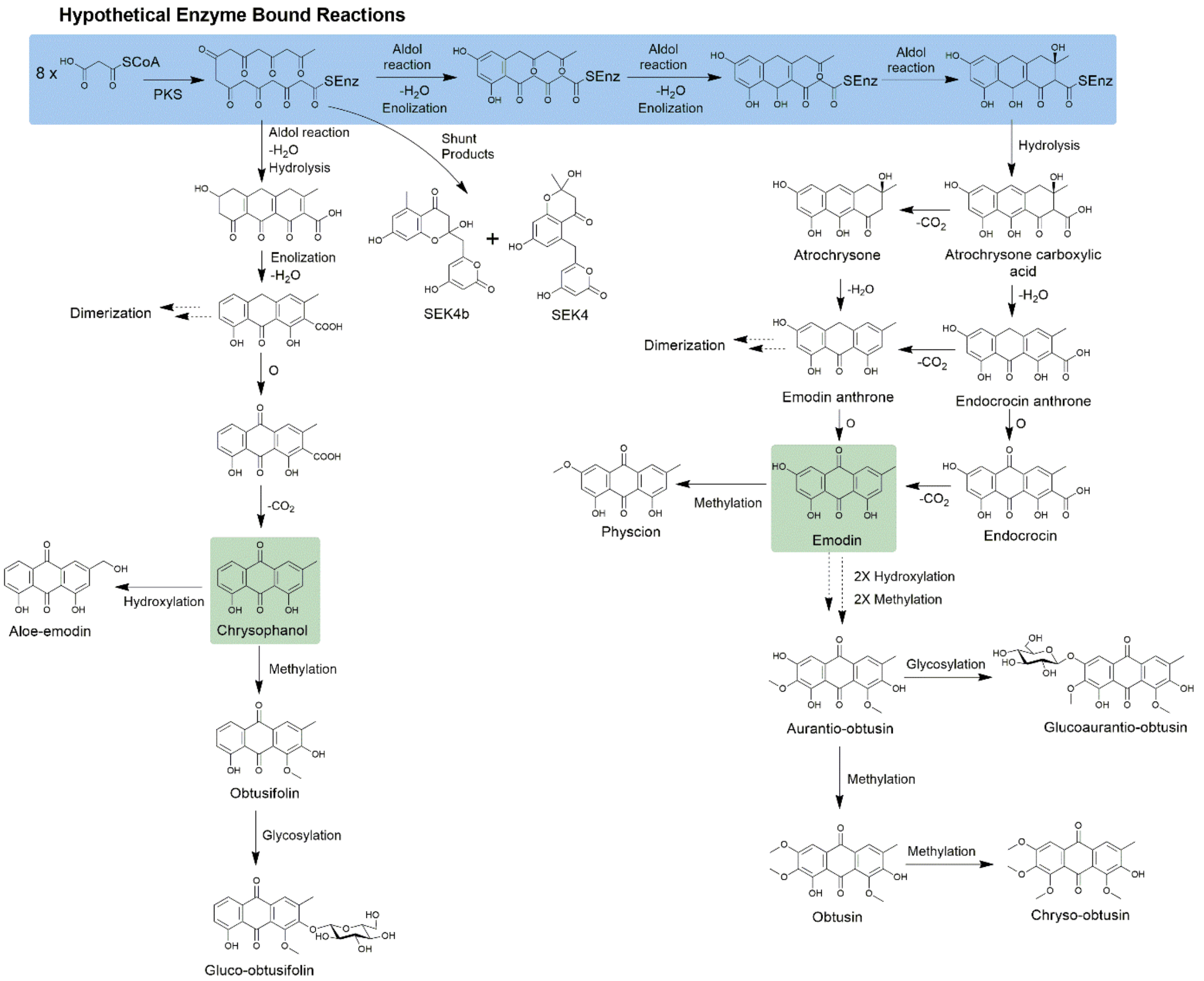

Supplementary Figure 1. Proposed PKS-mediated anthraquinone biosynthesis pathways. Eight molecules of malonyl-CoA are condensed to produce a linear octaketide non-reduced polyketide, which undergoes sequential cyclization and enolization (highlighted in blue shade), and released from PKS to produce atrochrysone carboxylic acid, the first PKS-produced anthranoid scaffold. Decarboxylation and oxidation results in final anthraquinones such as emodin. The intermediates might undergo dimerization reactions to produce anthraquinone dimers. Biosynthesis of anthraquinones such as chrysophanol and islandicin follows different dehydration and enolization steps. Final anthraquinones highlighted in green shade. The possible biosynthesis routes of post-modified anthraquinones detected at different phases of seed development are also shown.

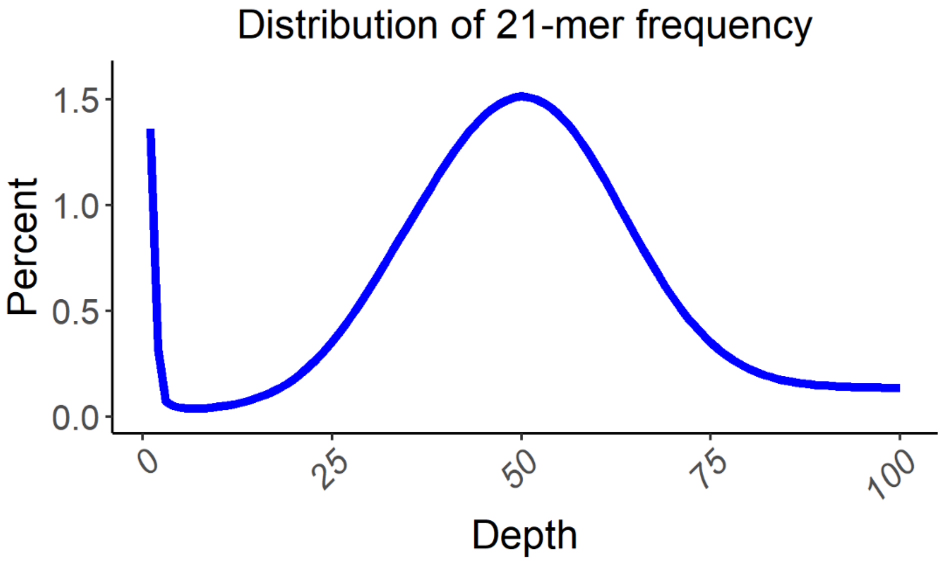

Supplementary Figure 2. Genome size estimation from distribution of 21-mer frequency in the sequencing reads. The reads used for *k*-mer distribution analysis were from the 200 bp paired-end library. A total of 27.5 Gb high-quality short-reads were used and only one peak was observed.

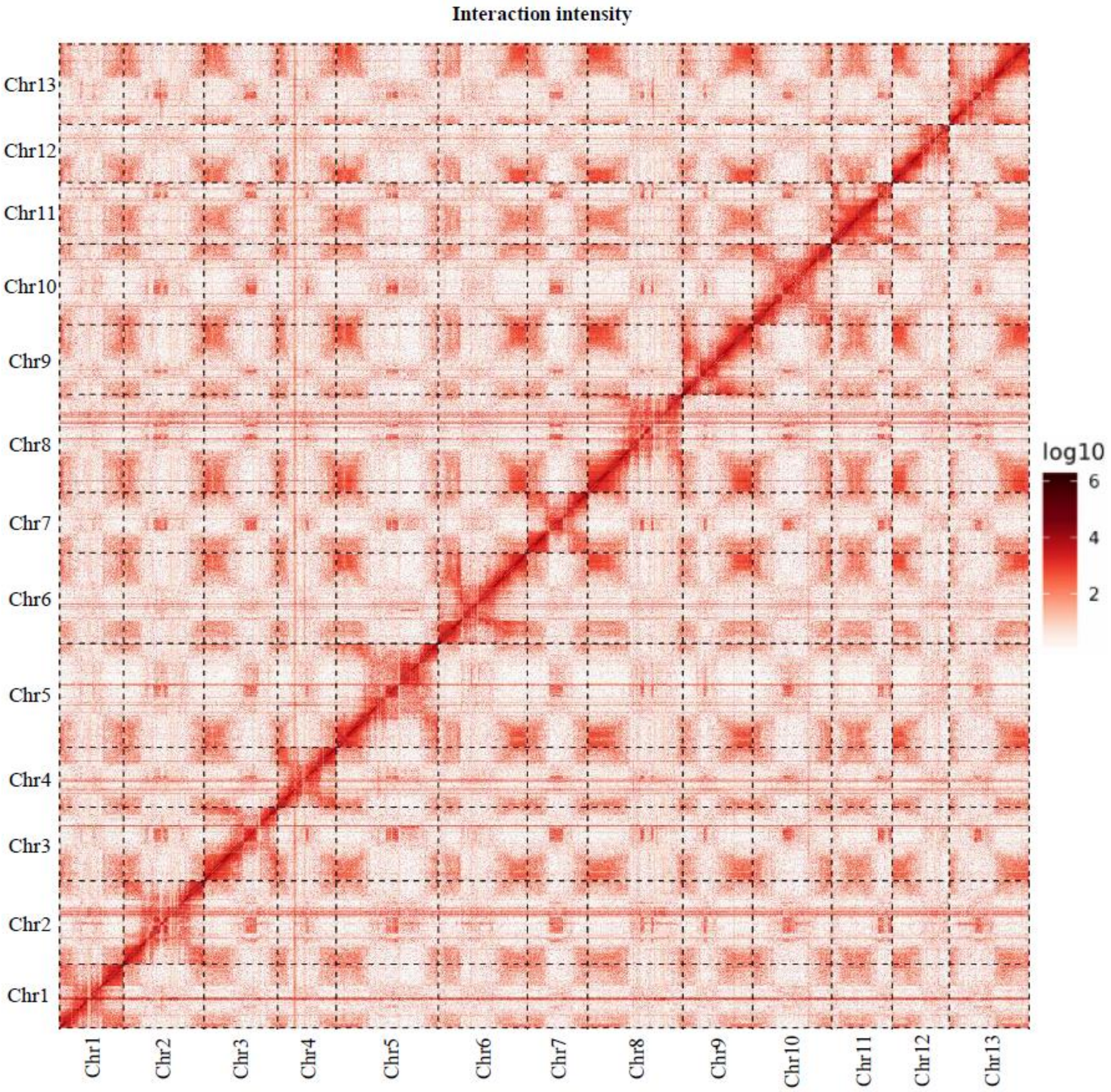

Supplementary Figure 3. Heatmap of chromosome conformation capture analysis. The intensity of interaction indicates the normalized count of Hi-C link on a logarithmic scale. The colored bar on the right represents the strength of interaction.

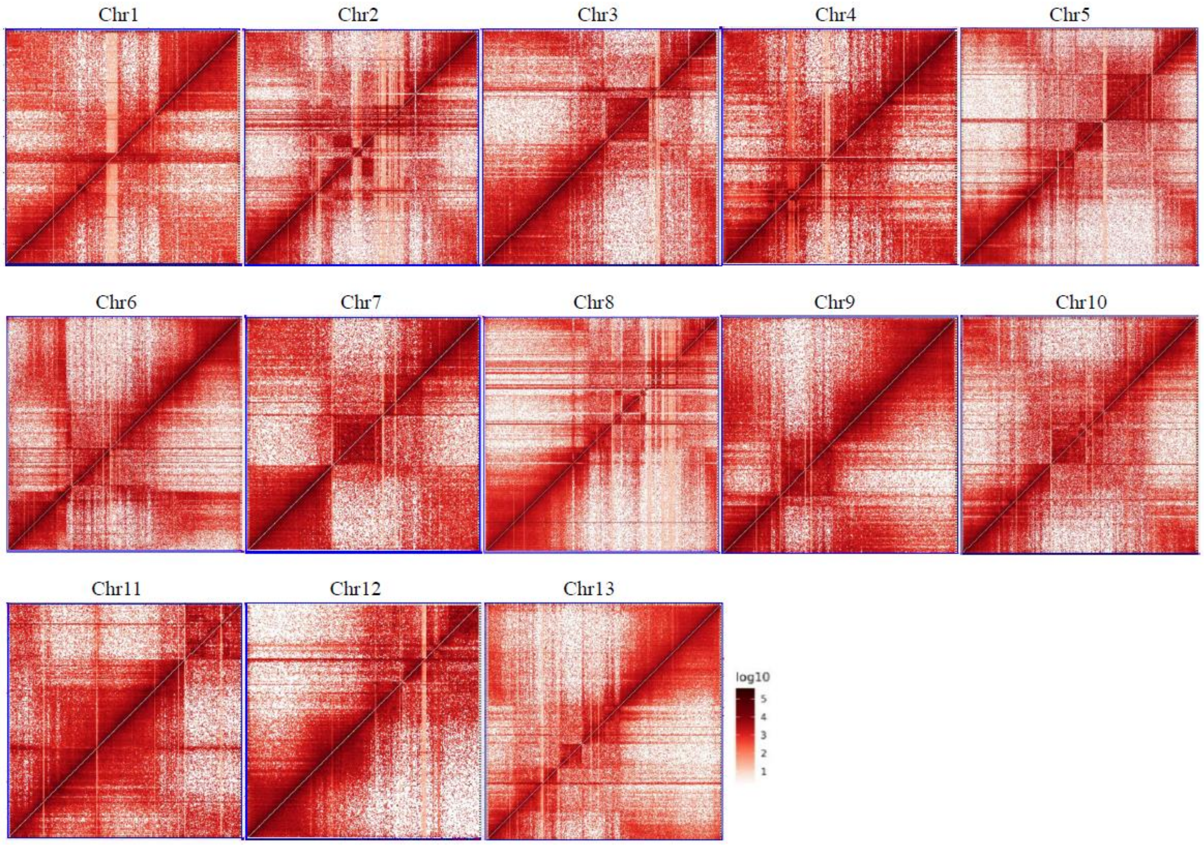

Supplementary Figure 4. Genome-wide analysis of chromatin interactions at 100-kb resolution in *S. tora* genome. The colored bar represents the strength of interaction.

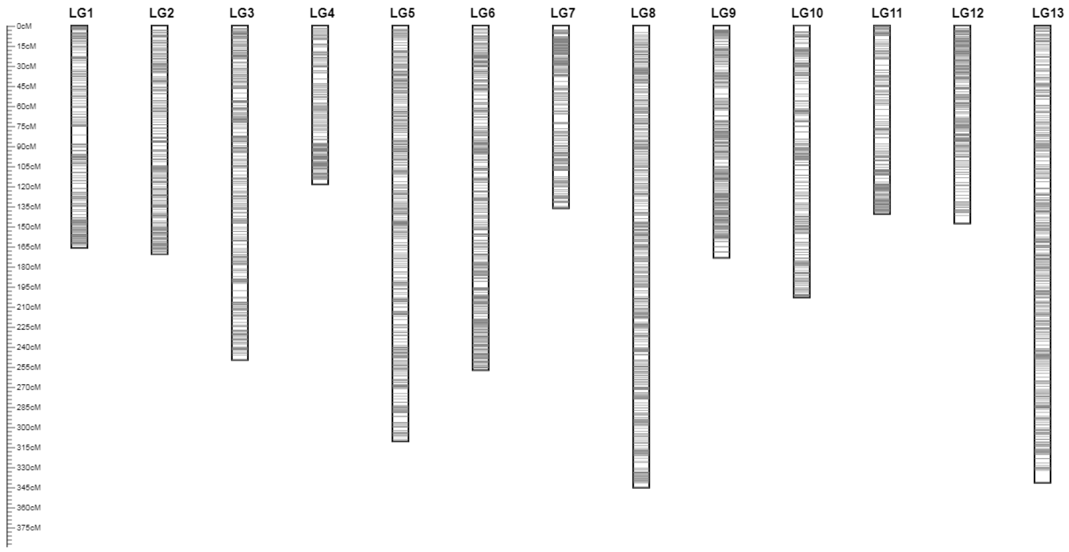

Supplementary Figure 5. Linkage map of an *S. tora* F_2 population derived from Myeongyun x ST-9. Linkage map was generated by integrating maps from two independent F_2 libraries. Gray bands in each linkage group indicate mapped markers. Numbers of each linkage group correspond to the numbering of chromosomes in this work.

Comparative Genome Structure (Chr03 and B050-B02)

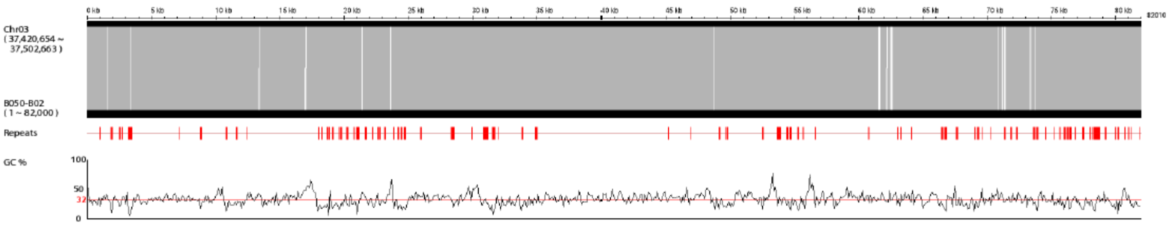

Comparative Genome Structure (Chr05 and B020-G17)

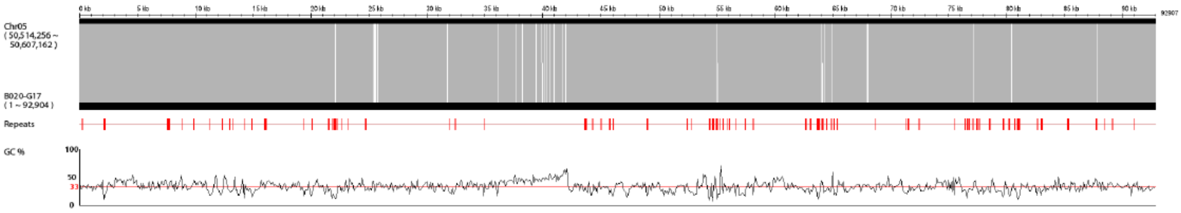

Comparative Genome Structure (Chr06 and B016-D19)

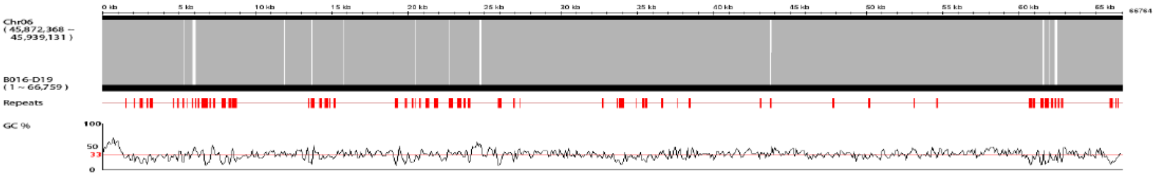

Comparative Genome Structure (Chr06 and H036-G09)

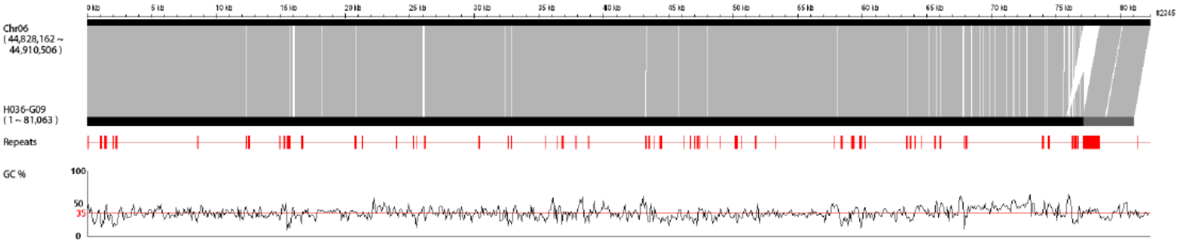

Comparative Genome Structure (Chr08 and H001-O11)

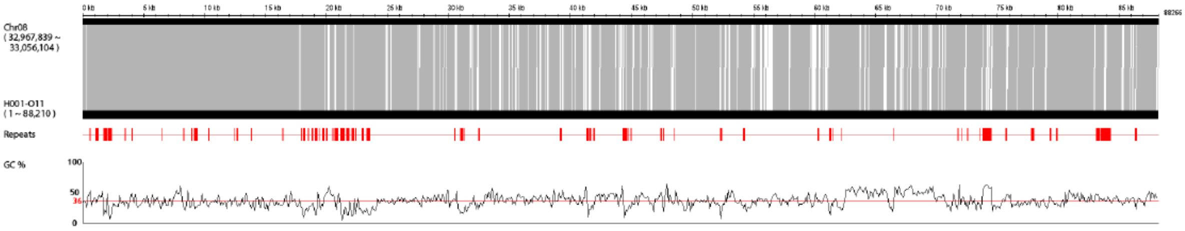

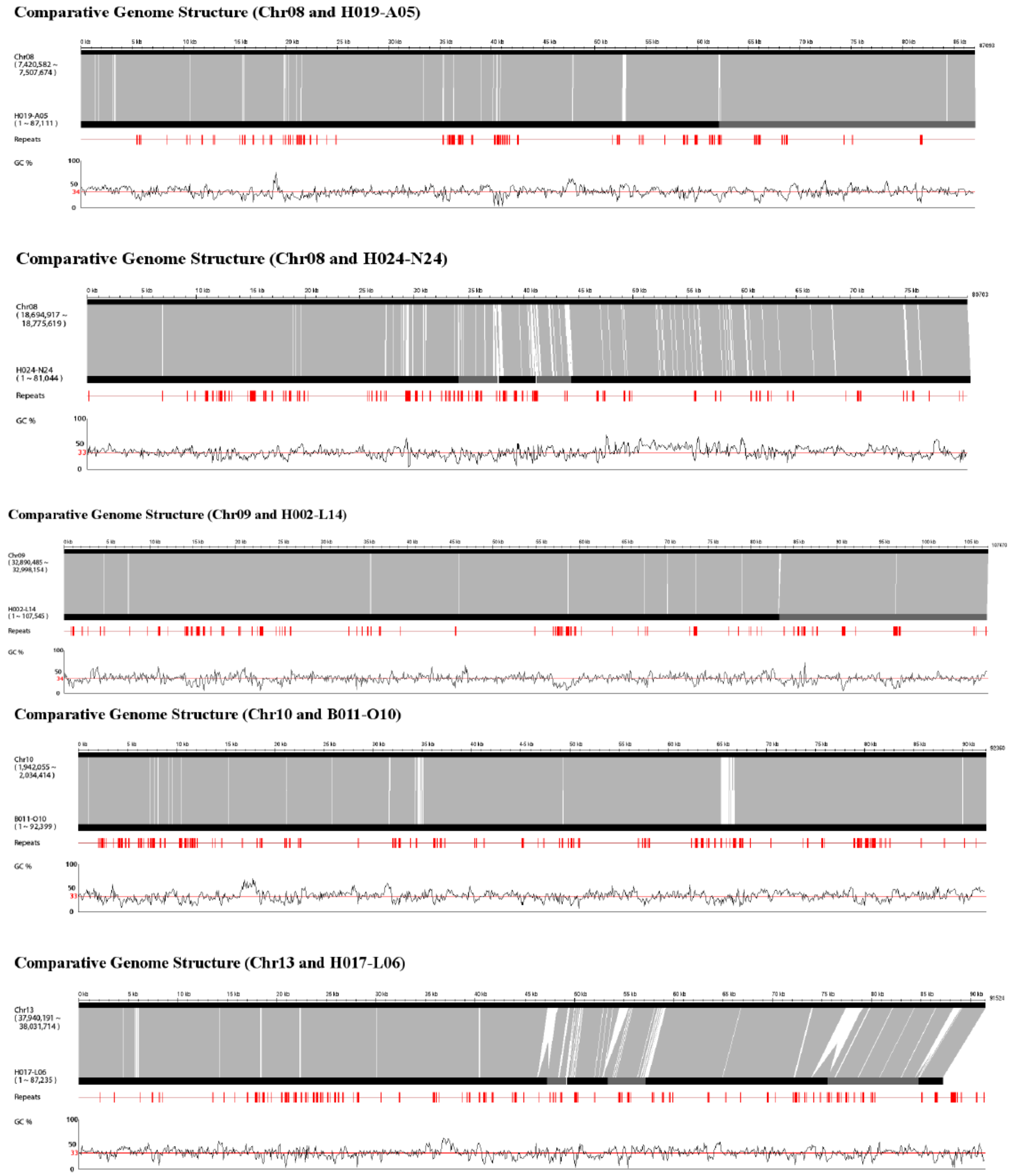

Supplementary Figure 6. Genome coverage evaluated by ten fully sequenced BAC clones sequenced by Sanger technology and 454 Life Sciences GS FLX System. Repeats (transposable elements) and GC contents (%) are also shown.

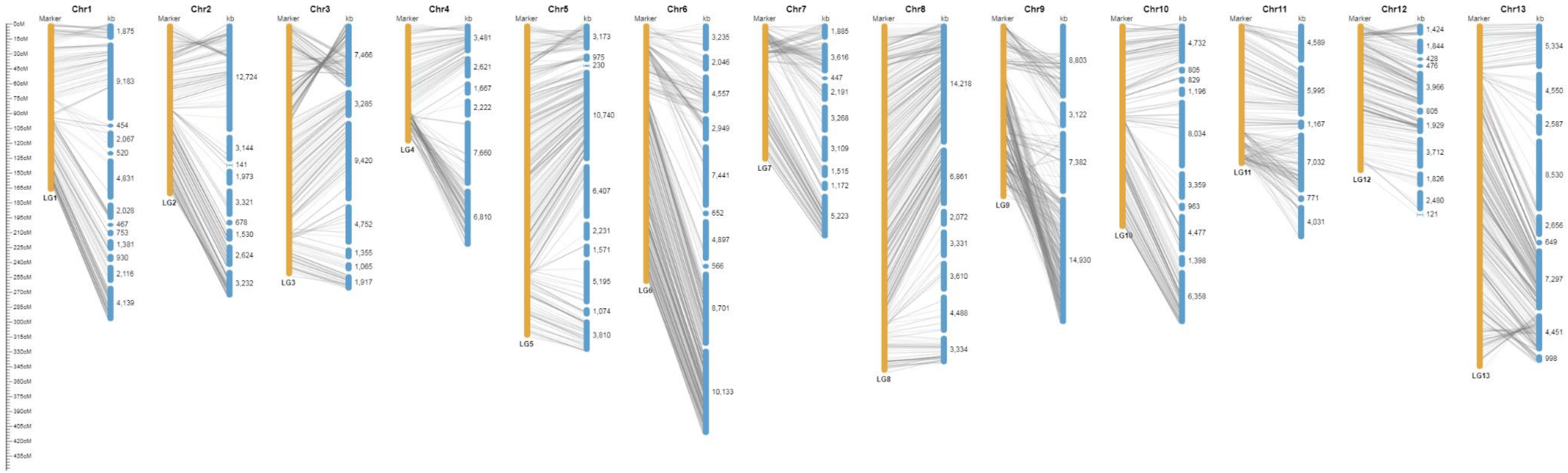

Supplementary Figure 7. Alignment of the genome sequence assembly with the genetic map of diploid *S. tora*. Assembled scaffolds (blue; 401.1 Mb, or 76.2% of the assembled genome sequence) were anchored to the thirteen linkage groups with 4,455 genetic markers (orange). Blue scaffolds were anchored and oriented using the Hi-C data. Numbers next to blue bars indicate length in nucleotides (kb).

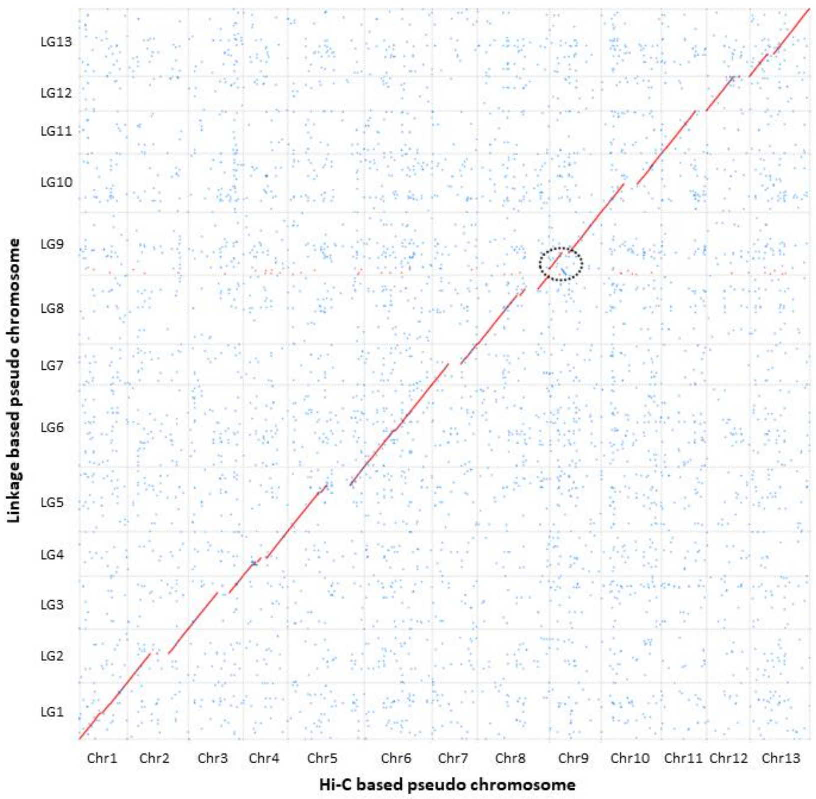

Supplementary Figure 8. Corroboration of pseudo chromosomes constructed by the genetic map and Hi-C approaches. Dotted circle indicates one major discrepancy between Chr9 and LG9.

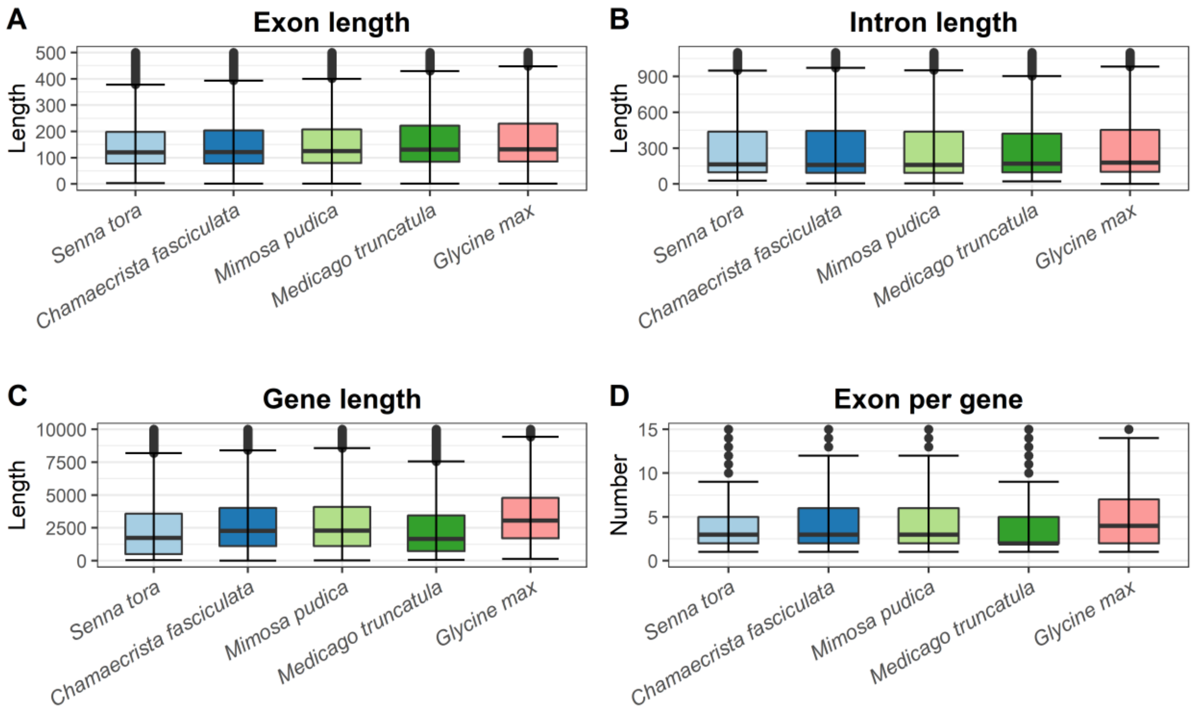

Supplementary Figure 9. Comparison of gene model features of *S. tora*, *C. fasciculate*, *M. pudica*, *M. truncatula*, and *G. max*. The length distributions of (A) exons, (B) introns, (C) genes, and (D) exon per gene are shown. The minimum and maximum bars are set at 1.5 * interquartile range (IQR). The boxes represent the IQR starting at the 25th percentile (Q1), including the middle line as the median, and ending with the 75th percentile (Q3). Points beyond the whiskers indicate outliers.

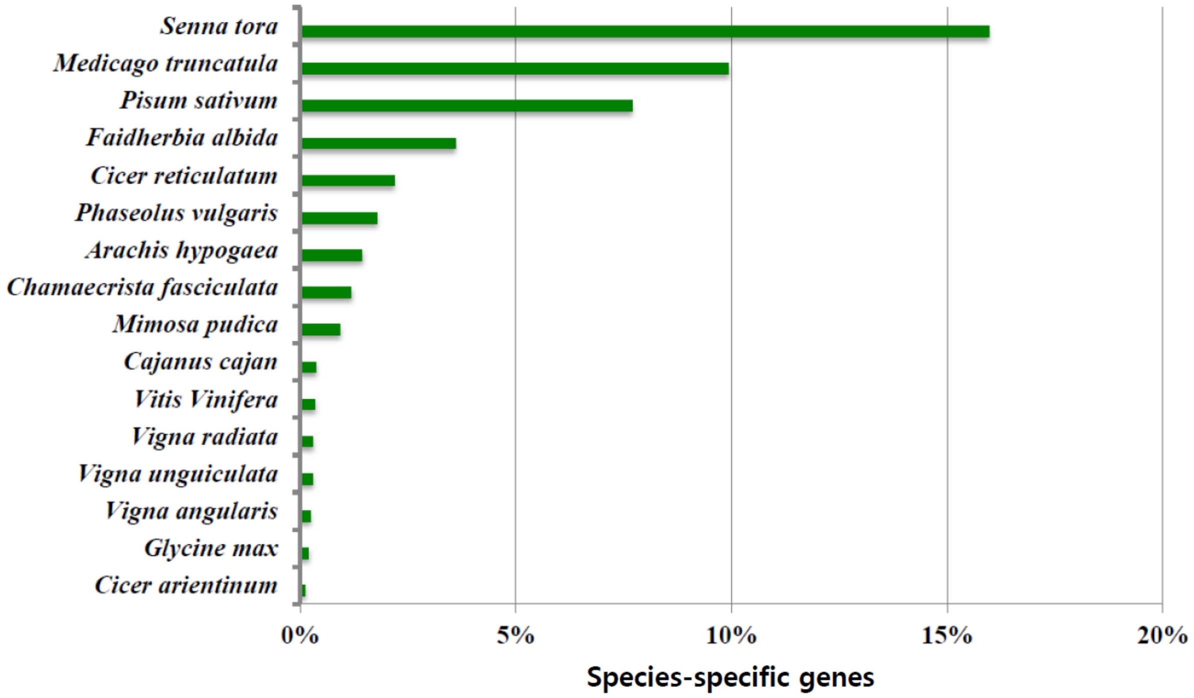

Supplementary Figure 10. Percentage of species-specific genes from 16 plant species.

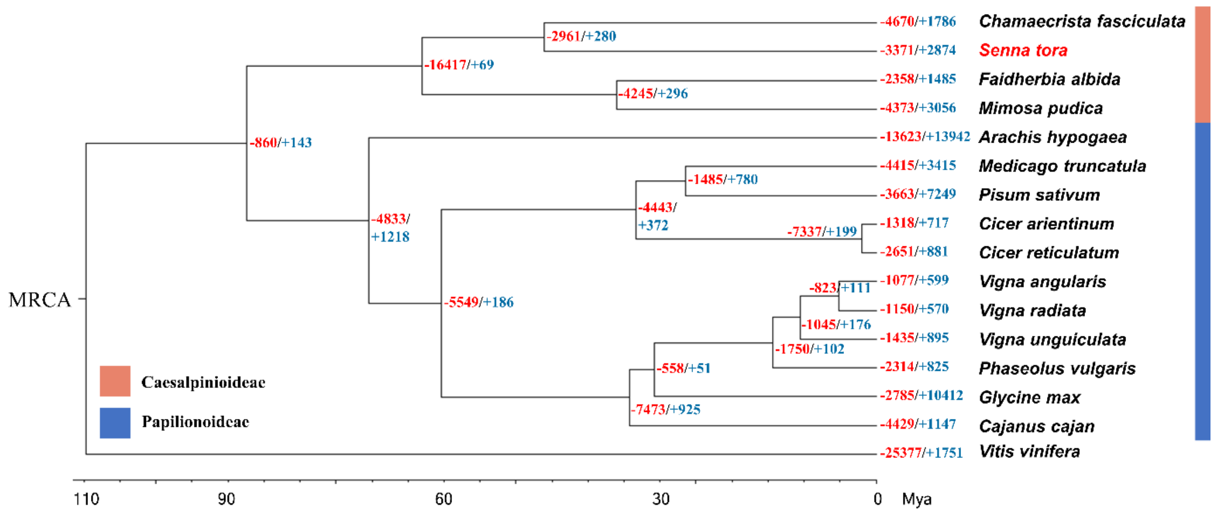

Supplementary Figure 11. Phylogenetic relationship and the expansion and contraction of gene families between Caesalpinioideae and Papilionoideae. Numbers on the branches show the number of gene gains (+, blue) and losses (-, red). The divergence times (MYA: Million Years Ago) are indicated by the scale bar at the bottom. *V. vinifera* was used as an outgroup. MRCA: Most Recent Common Ancestor.

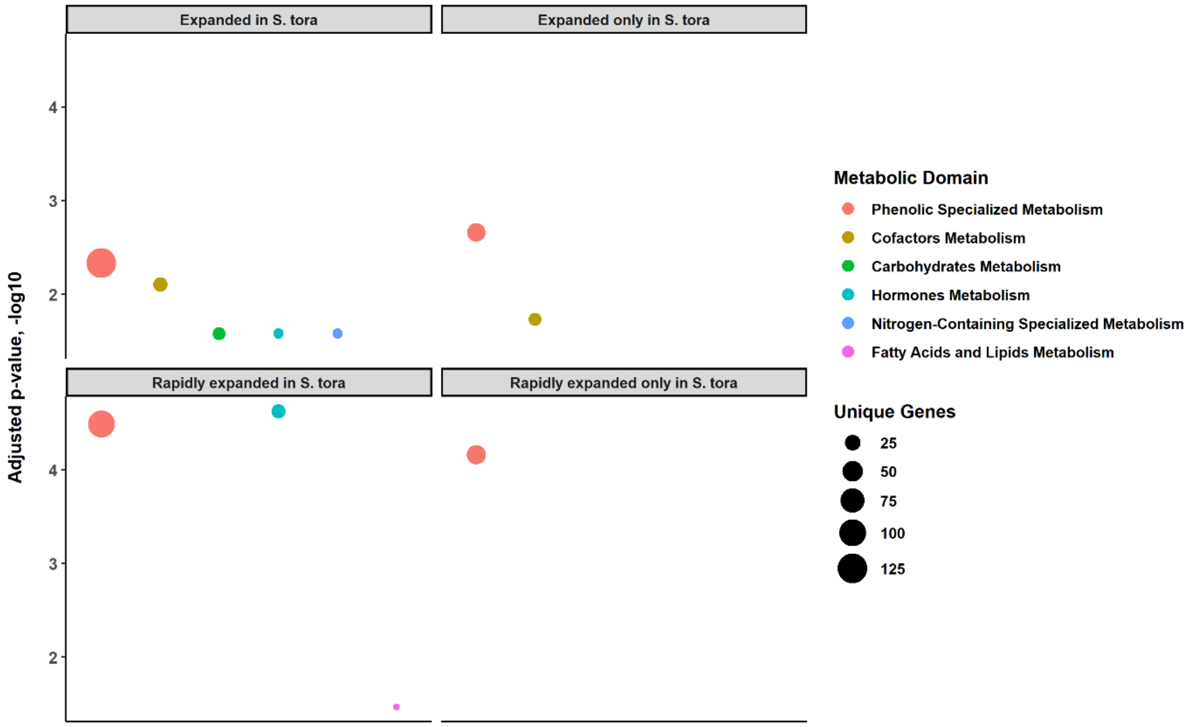

Supplementary Figure 12. Enriched StoraCyc metabolic domains of gene families that expanded in *S. tora*.

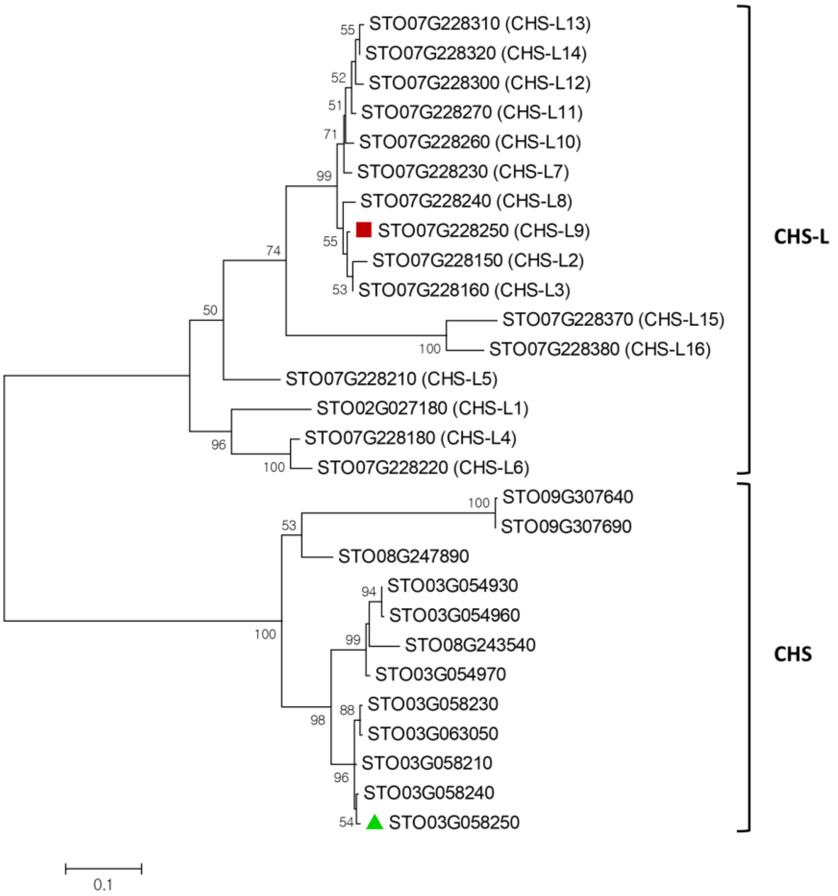

Supplementary Figure 13. The phylogenetic tree of CHS (12 genes) and CHS-L (16 genes) gene families in *S. tora*. This phylogenetic tree was generated using the maximum likelihood (ML) method with 1,000 bootstraps by MEGA v7.0 (https://www.megasoftware.net/), after alignment of predicted amino acid sequences by MUSCLE. Bootstrap support values (≥50%). Red square and green triangle indicate the CHS-L and CHS enzymes that were biochemically characterized in this study.

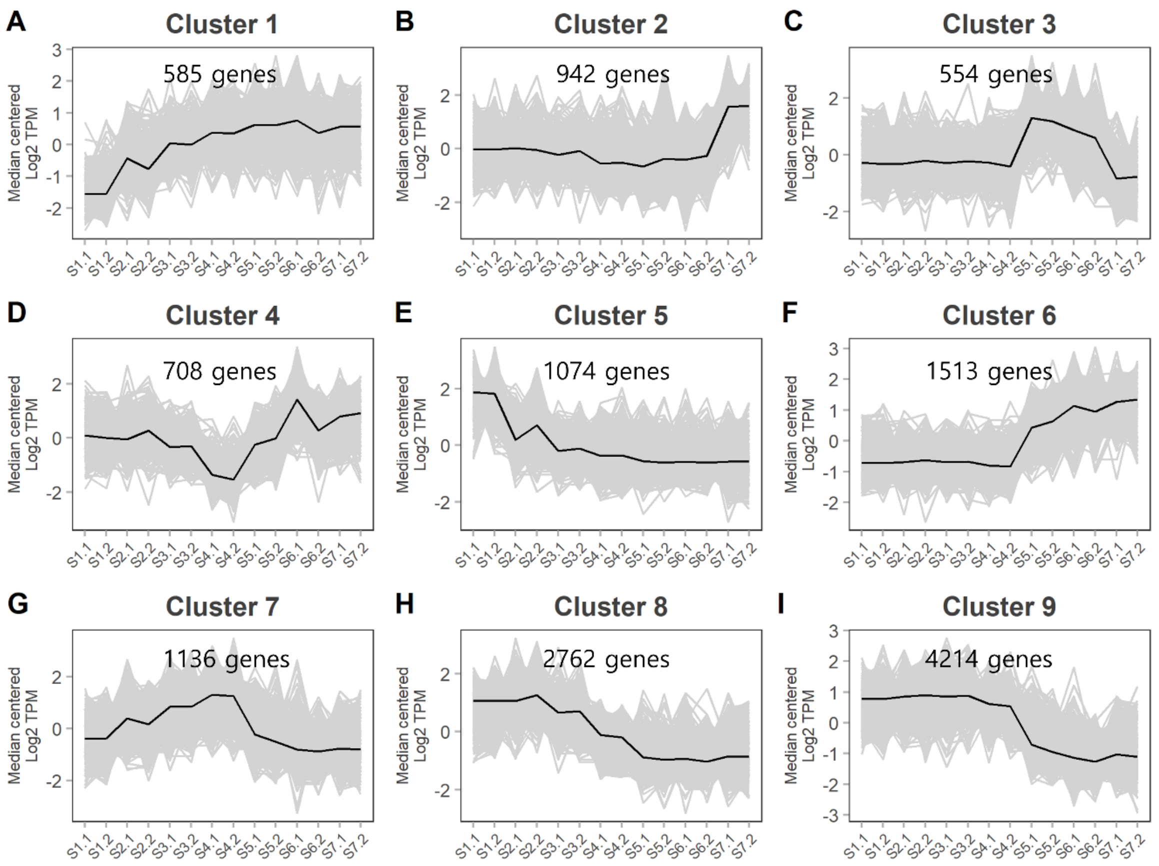

Supplementary Figure 14. Scaled transcript expression profiles of representative gene co-expression clusters during seed development in *S. tora*. TPM: transcripts per million. Numbers in x-axis indicate seed stages (S1 to S7) and two biological replicates (.1 and .2).

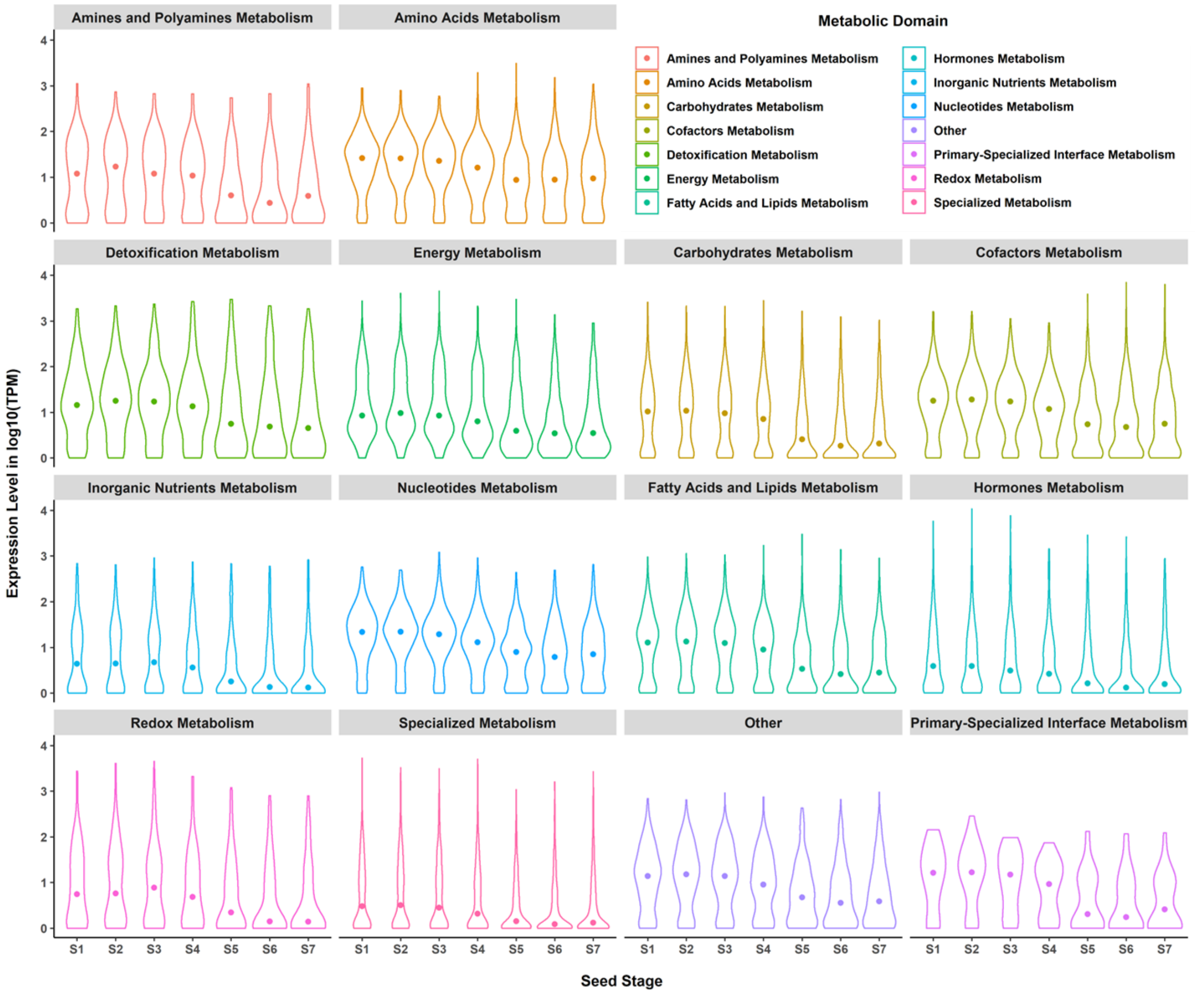

Supplementary Figure 15. Metabolic gene expression of 14 metabolic domains in StoraCyc throughout seed maturation. Dots represent median expression.

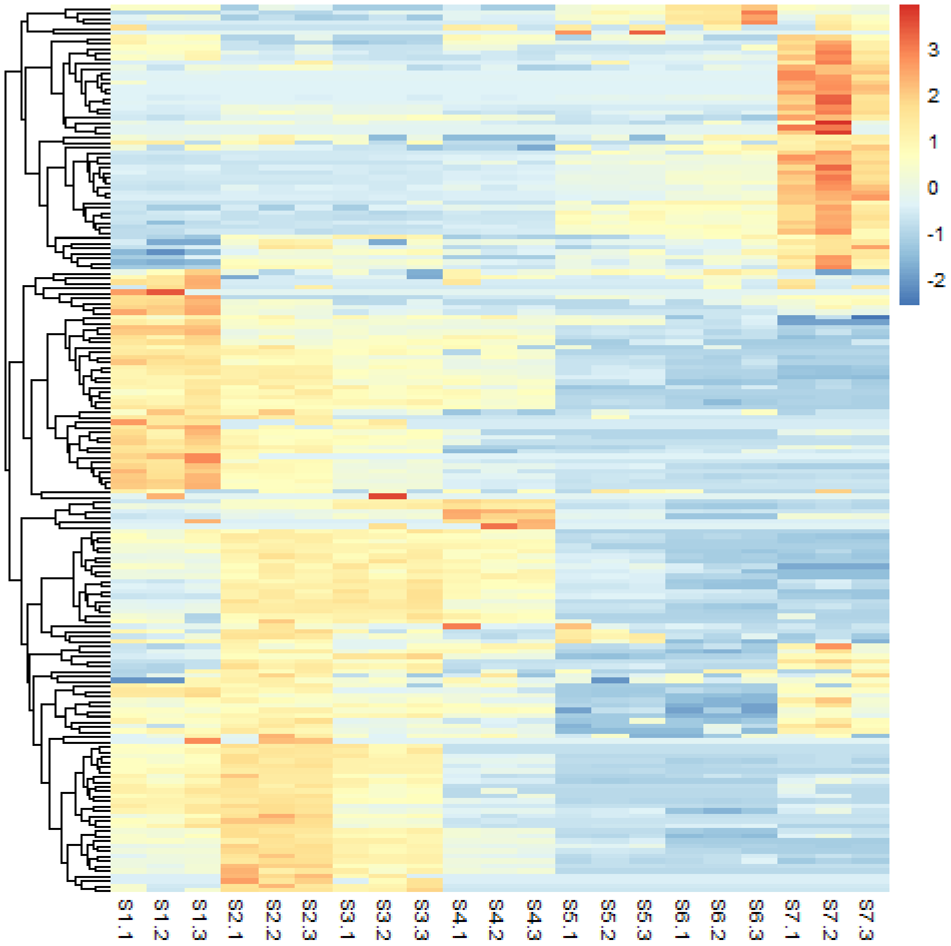

Supplementary Figure 16. Hierarchical clustering analysis of primary metabolites from seven developmental stages of *S. tora* seeds. Heatmap was drawn for the relative area of 178 putative metabolites using pheatmap R package v1.0.12 with hierarchical clustering. X-axis labels indicate seed stages (S1 to S7) and three biological replicates (.1, .2, .3).

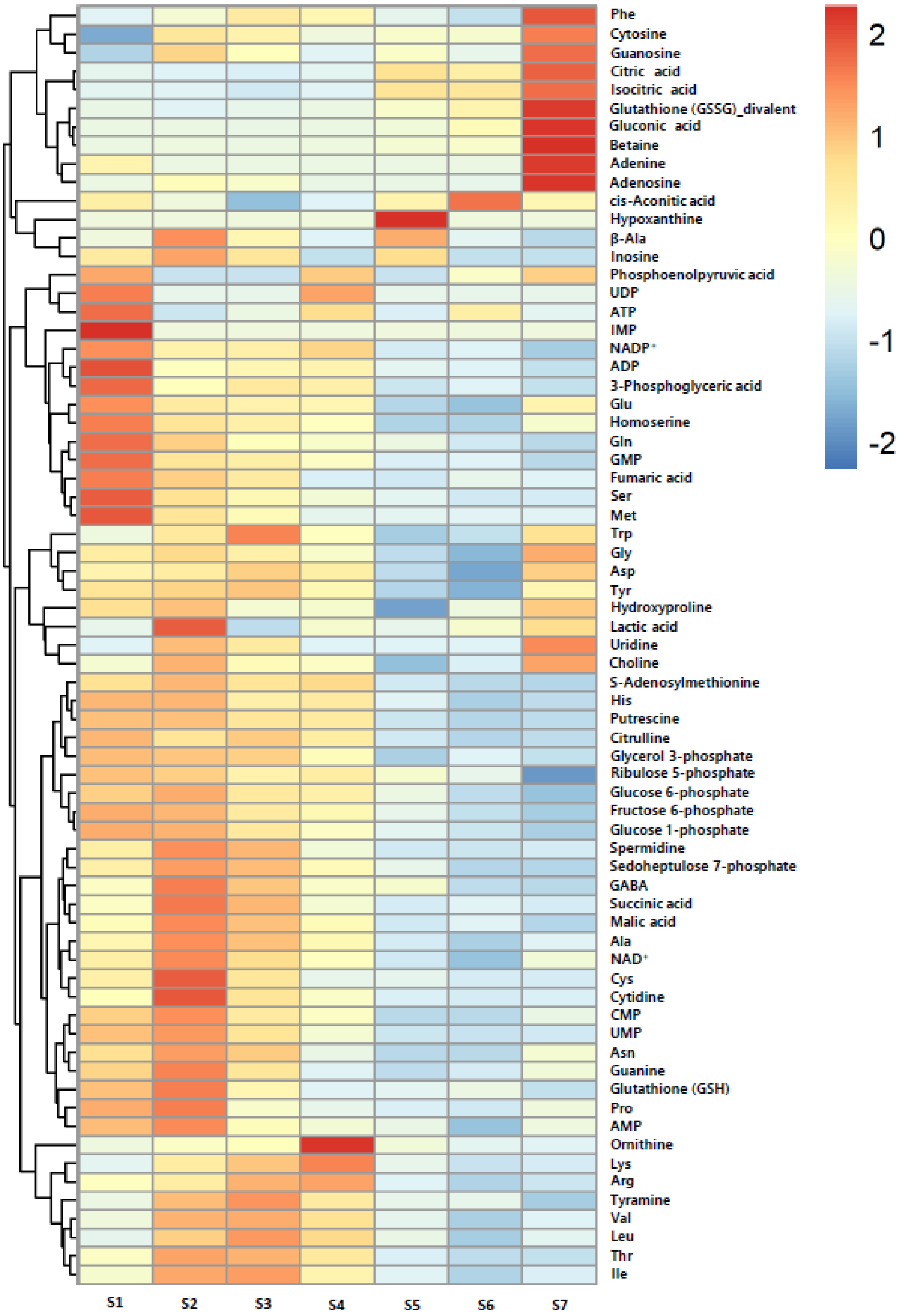

Supplementary Figure 17. Quantitative estimation of 69 primary metabolites during seed development. Heatmap indicates normalized concentration from three biological replicates.

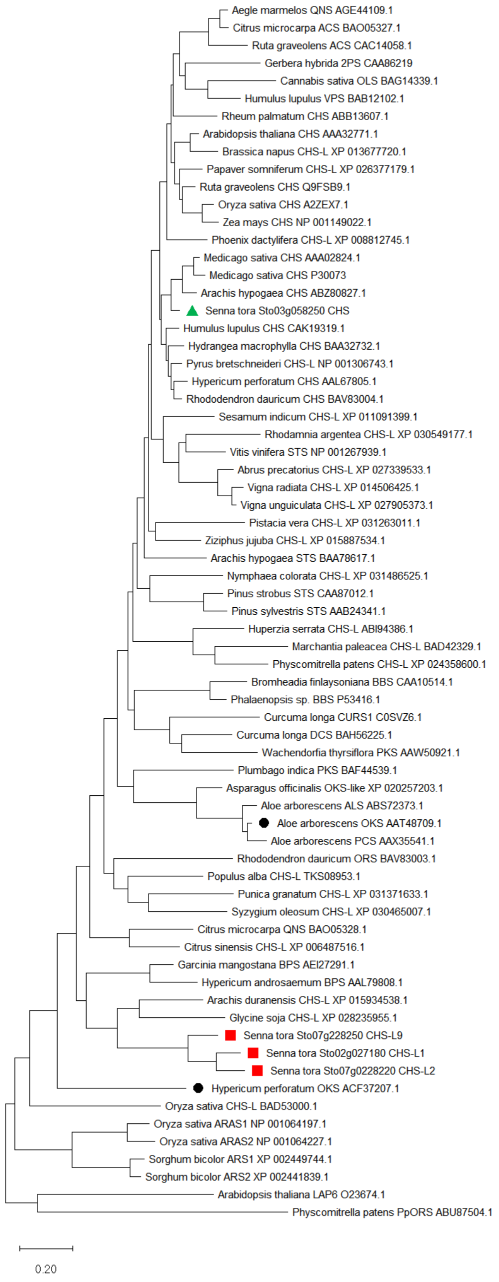

Supplementary Figure 18. The phylogenetic tree analysis of selected CHS and CHS-L genes in *S. tora* and other plant species. This phylogenetic tree was generated using the Maximum Likelihood method and JTT matrix-based model[1] after alignment of predicted amino acid sequences by MUSCLE. The tree with the highest log likelihood (-29513.52) is shown. Initial tree(s) for the heuristic search were obtained automatically by applying Neighbor-Join and BioNJ algorithms to a matrix of pairwise distances estimated using the JTT model, and then selecting the topology with superior log likelihood value. The tree is drawn to scale, with branch lengths measured in the number of substitutions per site. This analysis involved 69 amino acid sequences. There were a total of 880 positions in the final dataset. Evolutionary analyses were conducted in MEGA X[2]. Green colored triangle and red colored rectangle represent the CHS and CHS-Ls from *S. tora* described in this study. Octaketide synthases from *Aloe arboresens* and *Hypericum perforatum* are labeled with black circle.

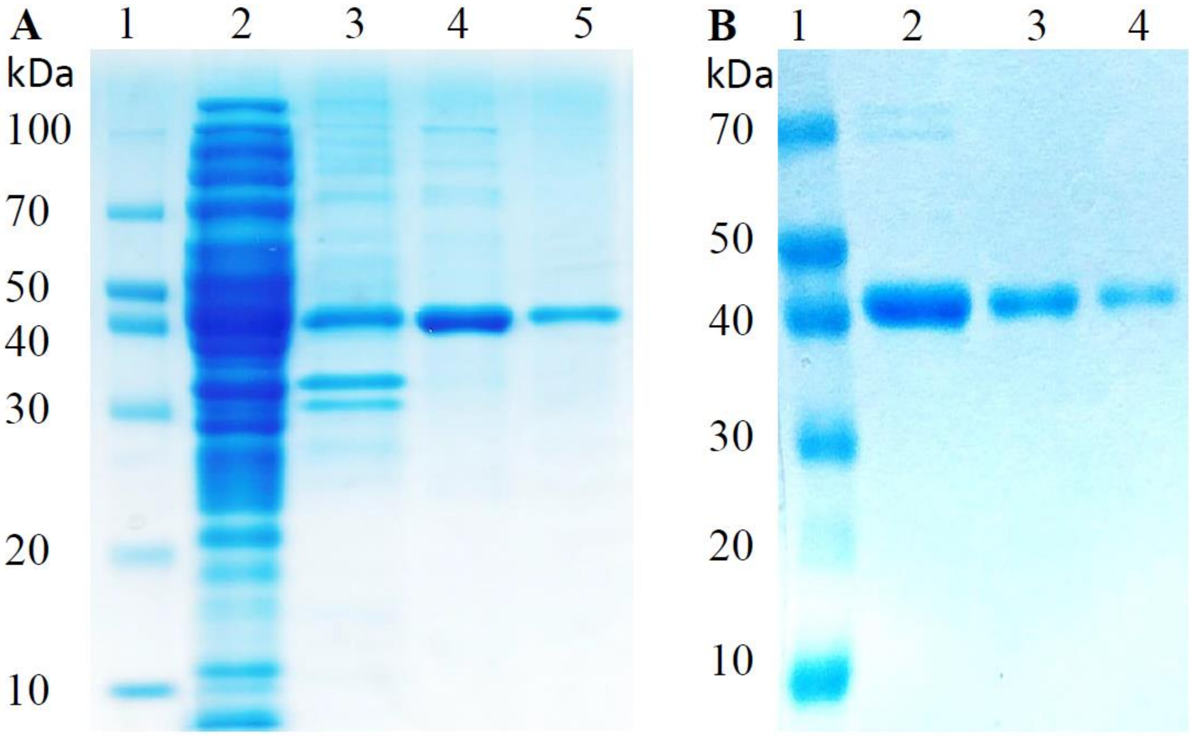

Supplementary Figure 19. 12%SDS-PAGE analysis. (A) STO07G228250 (CHS-L9). Lane 1: Standard protein ladder; Lane 2: total soluble fraction; Lane 3: total insoluble fraction; Lanes 4 and 5: pure protein fractions. The pure fractions of the protein were pooled and concentrated. (B) STO03G058250 (CHS). Lane 1: standard protein ladder; Lanes 2-4: pure soluble fractions. The pure fractions of the protein were pooled and concentrated. The experiments were reproduced at least three times. Source data are provided as a Source Data file.

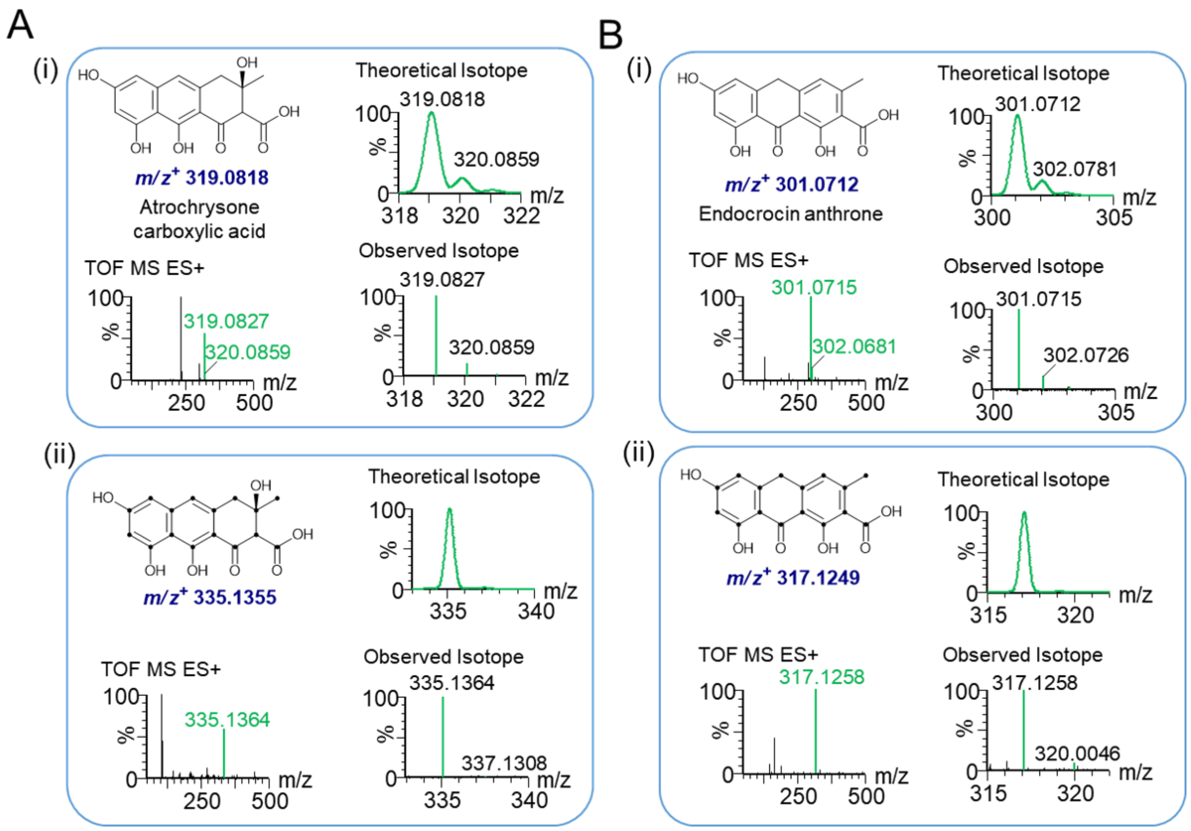

Supplementary Figure 20. TOF ESI-MS analysis of the anthranoids generated in reaction assays. (A) (i) TOF ESI-MS spectrum for the speculated product atrochrysone carboxylic acid with the molecular formula $C_{16}H_{14}O_7$ for which calculated theoretical exact mass was 319.0818 Da in proton adduct form. The theoretical mass isotope perfectly aligned to the observed mass isotope. (ii) ESI MS-spectrum for the speculated ^{13}C-labelled atrochrysone carboxylic acid with molecular formula $^{13}C_{16}H_{14}O_7$ for which calculated theoretical exact mass was 335.1355 Da in proton adduct form. The theoretical mass isotope perfectly aligned to observed mass isotope. (B) (i) TOF ESI-MS spectrum for the speculated product endocrocin anthrone with molecular formula $C_{16}H_{12}O_6$ for which the calculated theoretical exact mass was 301.0712 Da in proton adduct form. The theoretical mass isotope perfectly aligned to the observed mass isotope. (ii) TOF ESI-MS spectrum for the speculated ^{13}C-labelled endocrocin anthrone with molecular formula $^{13}C_{16}H_{12}O_6$ for which calculated theoretical exact mass was 317.1249 Da in proton adduct form. The theoretical mass isotope perfectly aligned to the observed mass isotope.

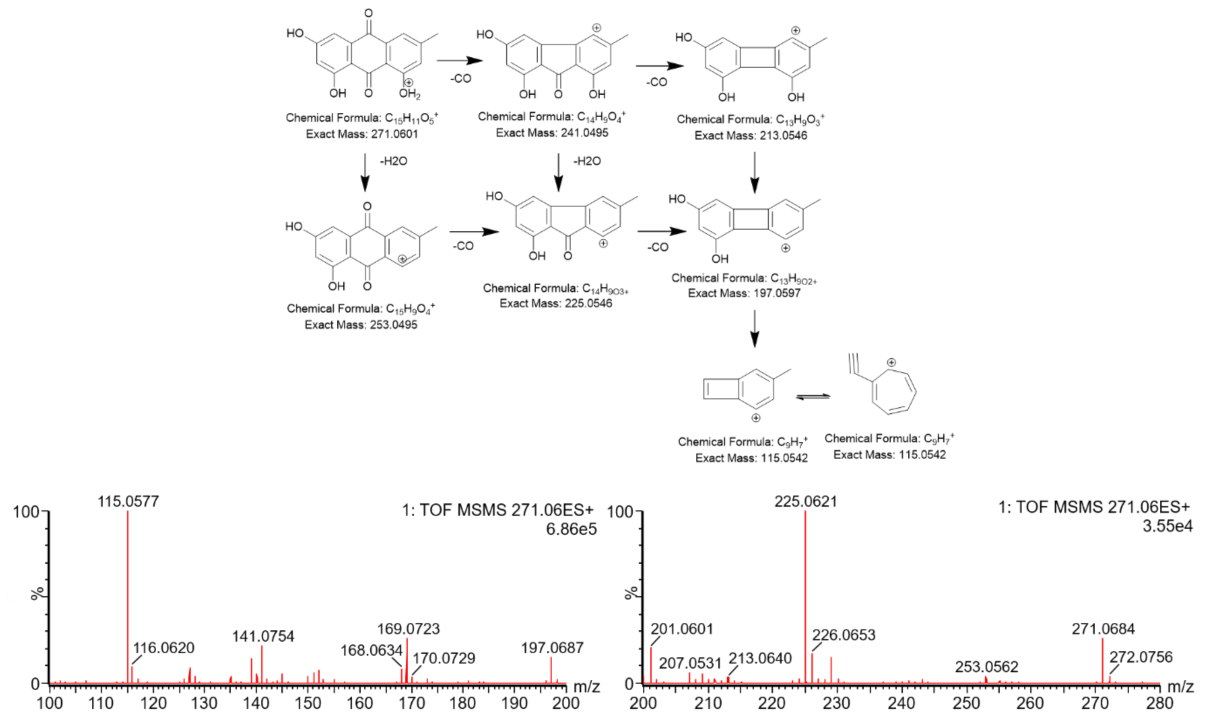

Supplementary Figure 21. ESI-MS^2 analysis of emodin (precursor ion 271) and proposed MS-MS fragmentation of emodin.

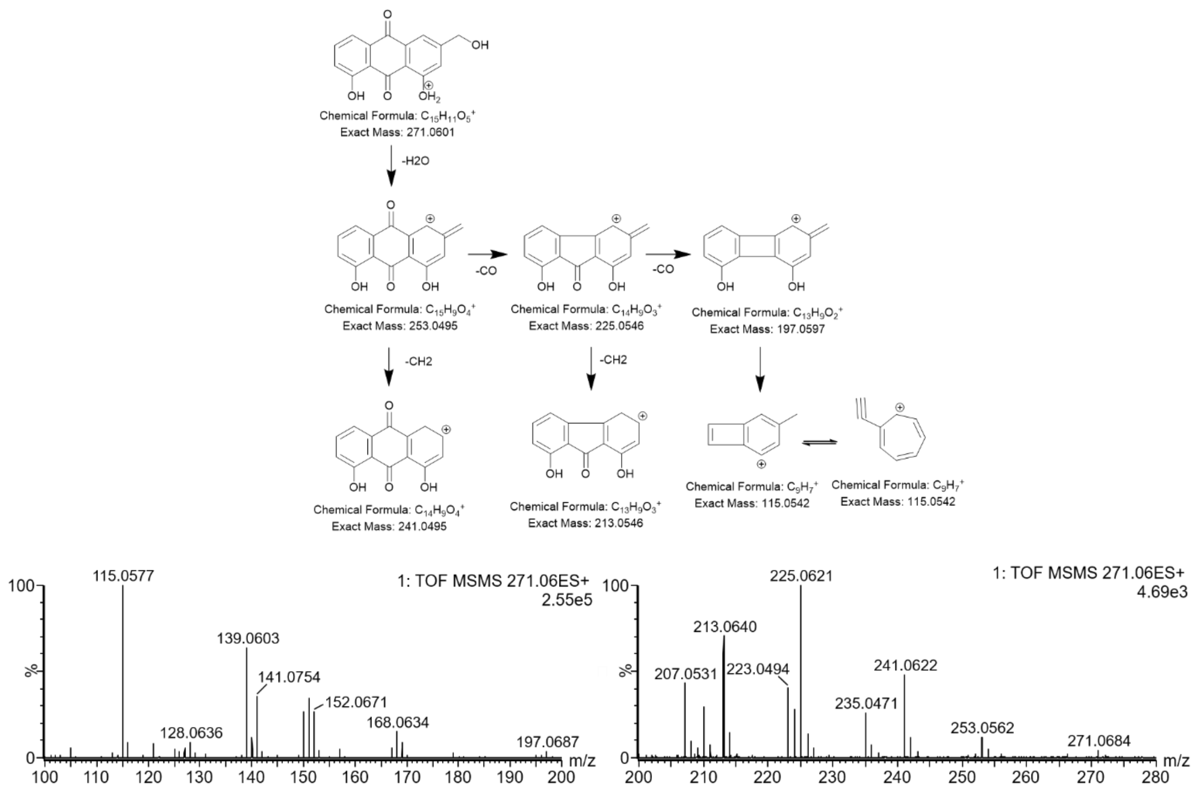

Supplementary Figure 22. ESI-MS^2 analysis of aloe-emodin (precursor ion 271) and proposed MS-MS fragmentation of aloe-emodin.

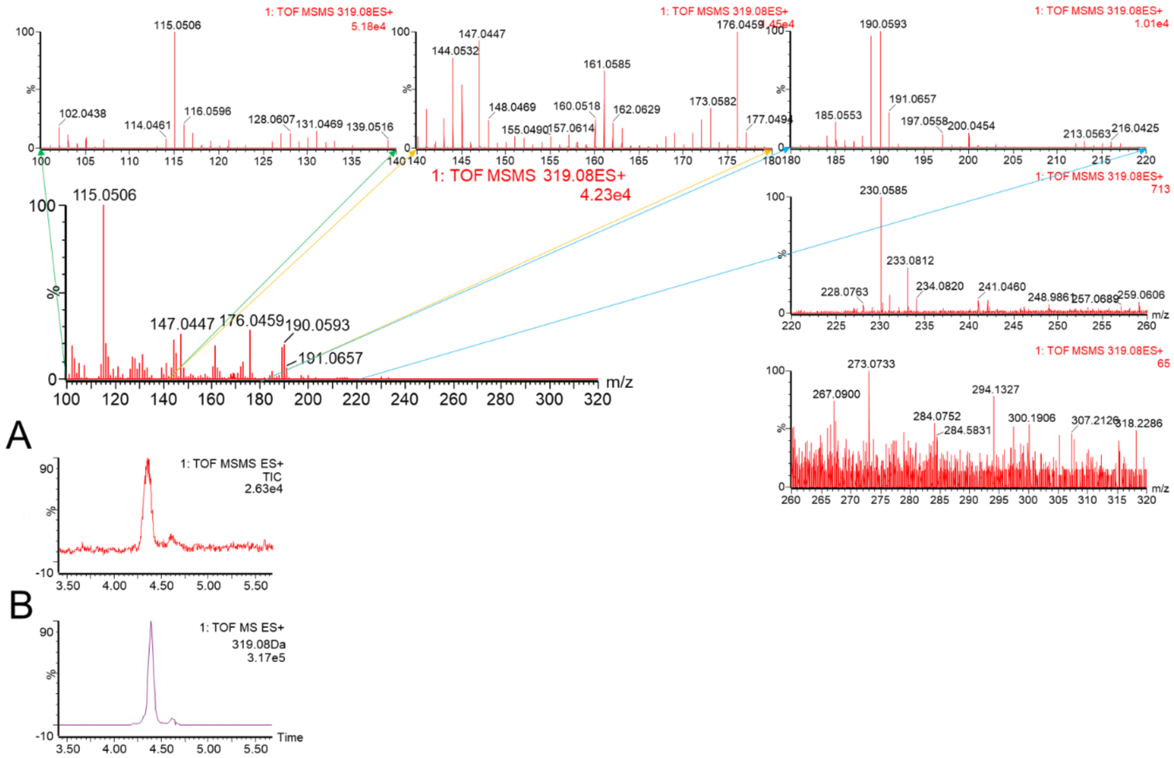

Supplementary Figure 23. High resolution ESI-MS and ESI-MS^2 analysis of atrochrysone carboxylic acid (precursor ion 319.08), one of the metabolites observed in CHS-L9 (STO07G228250) catalyzed in vitro reaction mixture. (A) Total ion chromatogram (TIC) of ESI-MS^2 for precursor ion 319 and (B) extracted ion chromatogram (EIC) of ESI-MS for mass 319.08 Da. Mass spectra extracted from ESI-MS^2 (precursor ion 319) are shown in expanded view. The structure for selected mass spectra observed in ESI-MS^2 analysis are proposed in Supplementary Fig. 24.

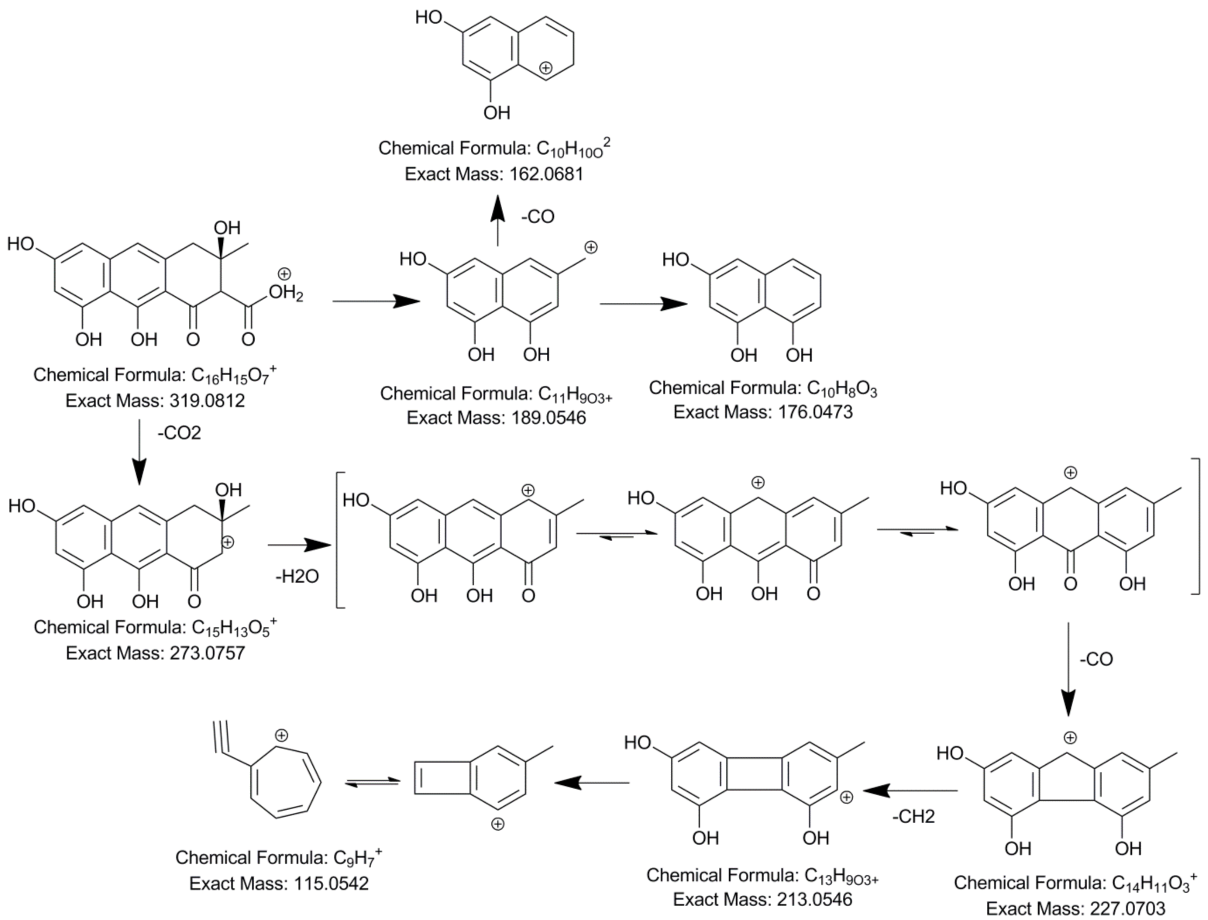

Supplementary Figure 24. Proposed ESI-MS^2 fragmentation of atrochrysone carboxylic acid based on ESI-MS^2 fragmentation pattern of emodin and aloe-emodin under identical instrumentation and analytical conditions. The structures correspond to the selected ESI-MS^2 spectra observed.

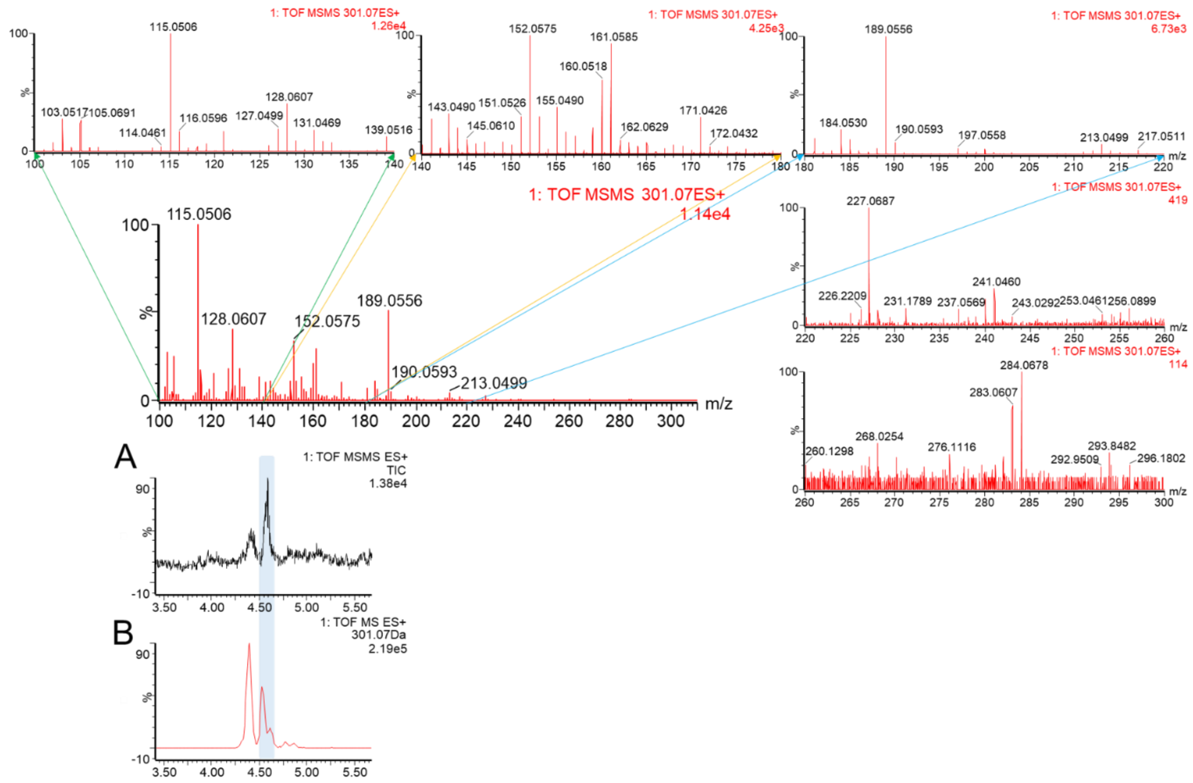

Supplementary Figure 25. High resolution ESI-MS and ESI-MS^2 analysis of endocrocin anthrone (precursor ion 301.07, shaded peak), one of the metabolites observed in CHS-L9 (STO07G228250) catalyzed *in vitro* reaction mixture. (A) Total ion chromatogram (TIC) of ESI-MS^2 for precursor ion 301 and (B) extracted ion chromatogram (EIC) of ESI-MS for mass 301.07 Da. Mass spectra extracted from ESI-MS^2 (precursor ion 301) are shown in expanded view. The structure for selected mass spectra observed in ESI-MS^2 analysis are proposed in Supplementary Fig. 26. The front peak corresponds to atrochrysone carboxylic acid.

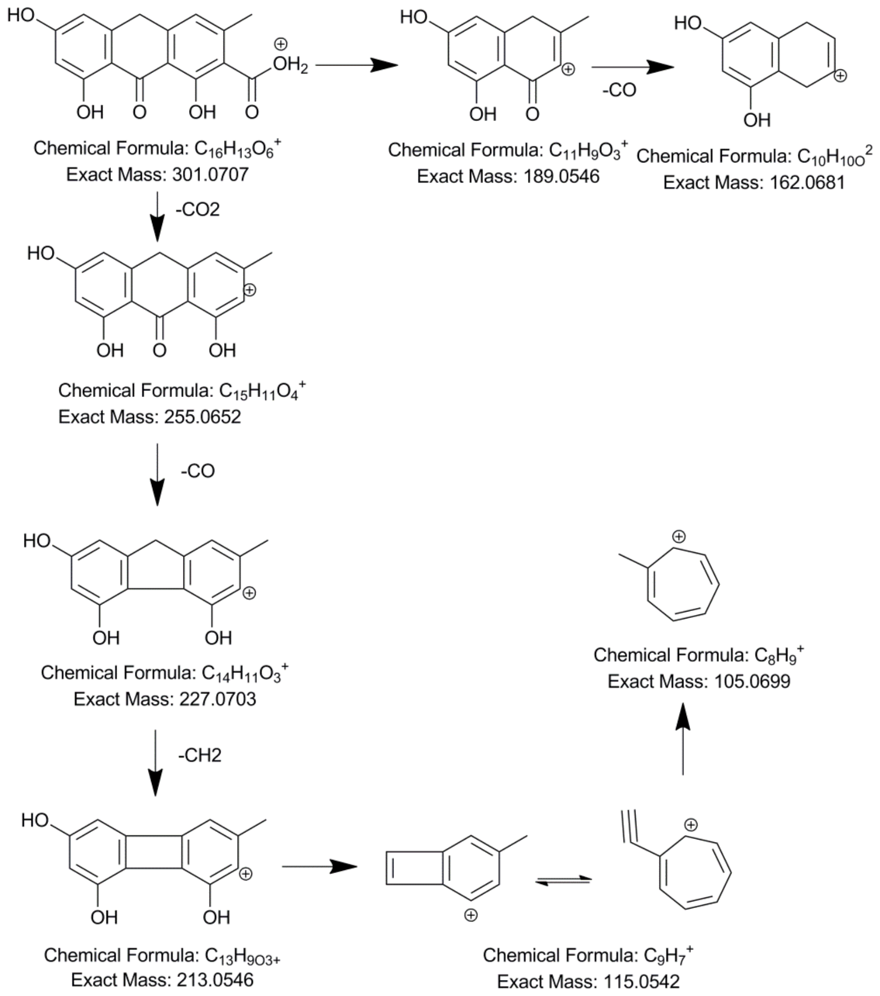

Supplementary Figure 26. Proposed ESI-MS^2 fragmentation of endocrocin anthrone based on ESI-MS^2 fragmentation pattern of emodin and aloe-emodin under identical instrumentation and analytical conditions. The structures correspond to the selected ESI-MS^2 spectra observed.

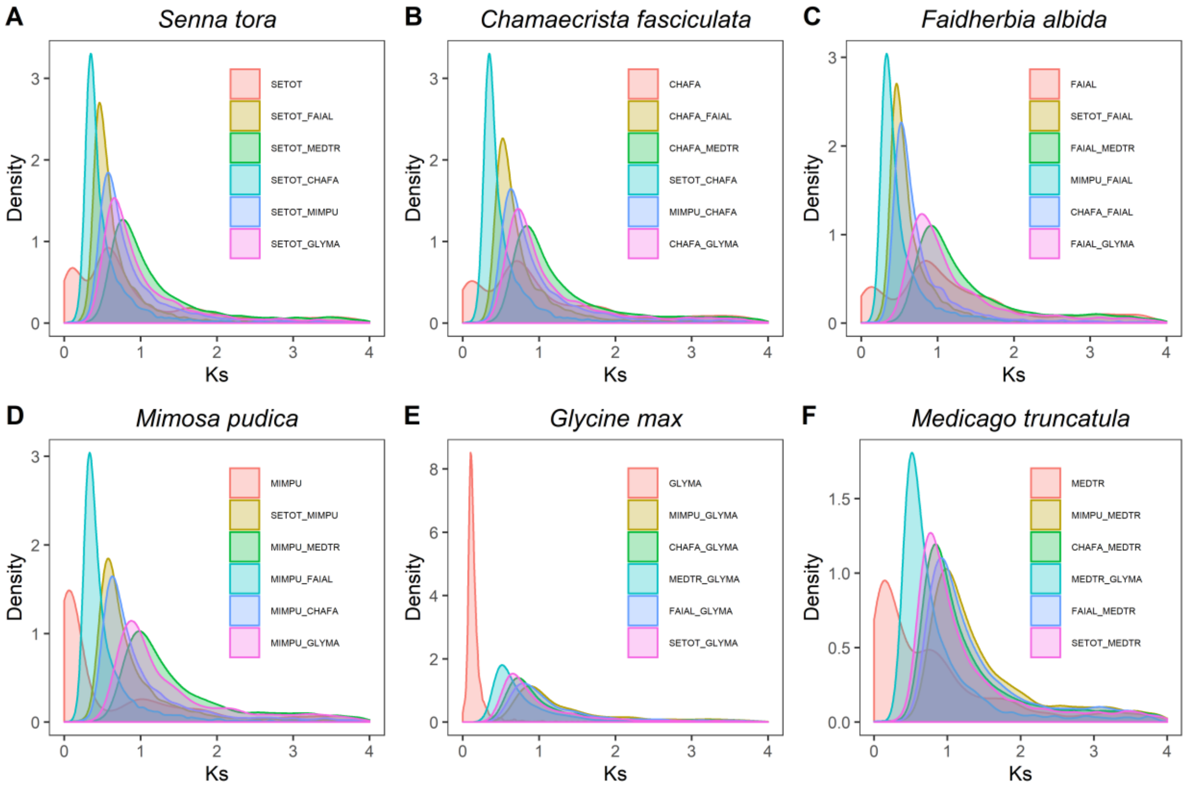

Supplementary Figure 27. Distribution of the synonymous substitution rate (Ks) among (A) *S. tora*, (B) *C. fasciculata*, (C) *F. albida*, (D) *M. pudica*, (E) *G. max*, and (F) *M. truncatula* in intra- and inter-genomic comparisons. Intra-genomic analysis indicates the WGD events and inter-genomic comparisons represent the divergence. *S. tora* (SETOT), *C. fasciculata* (CHAFA), *M. pudica* (MIMPU), *F. albida* (FAIAL), *M. truncatula* (MEDTR), *G. max* (GLYMA).

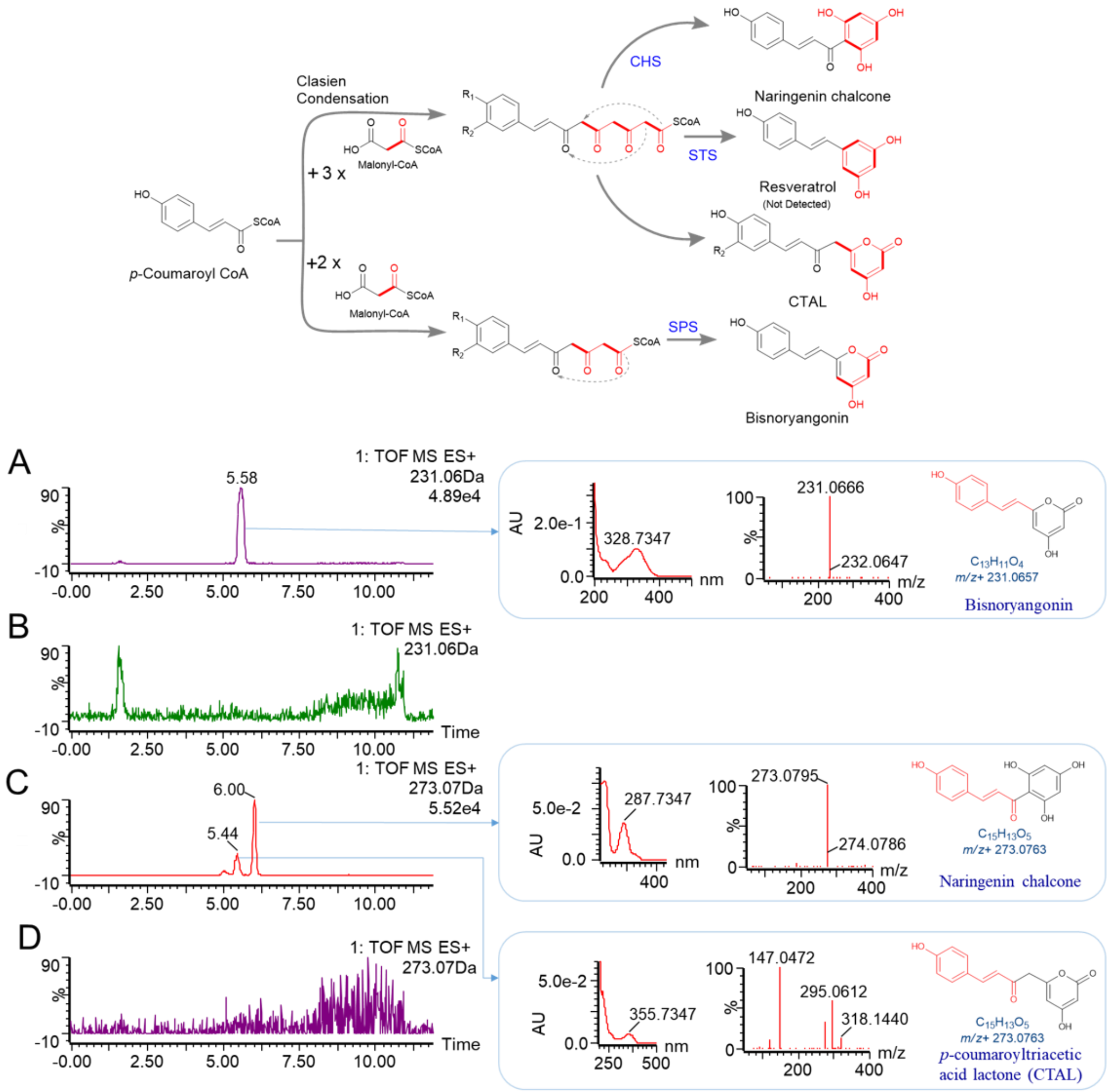

Supplementary Figure 28. High resolution TOF ESI-MS analysis of STO03G058250 (CHS) reaction mixture containing *p*-coumaroyl-CoA as a starter unit. Extracted ion chromatogram (EIC) for mass 231.06 Da in (A) reaction mixture and (B) reaction mixture containing only malonyl-CoA. EIC for mass 273.07 Da in (C) reaction mixture and (D) reaction mixture containing only malonyl-CoA. UV-VIS, ESI-MS spectrum along with their possible structure of each peak is shown with arrow. The CHS enzyme produced bisnoryangonin, naringenin chalcone, and *p*-coumaroyl triacetic acid lactone (CTAL) in the reaction mixture. Scheme in the top shows folding of the polyketide chain to produce different metabolites by different type III PKS enzymes. No stilbene type of product was detected. CHS: chalcone synthase; STS: stilbene synthase; and SPS: styryl pyrone synthase.

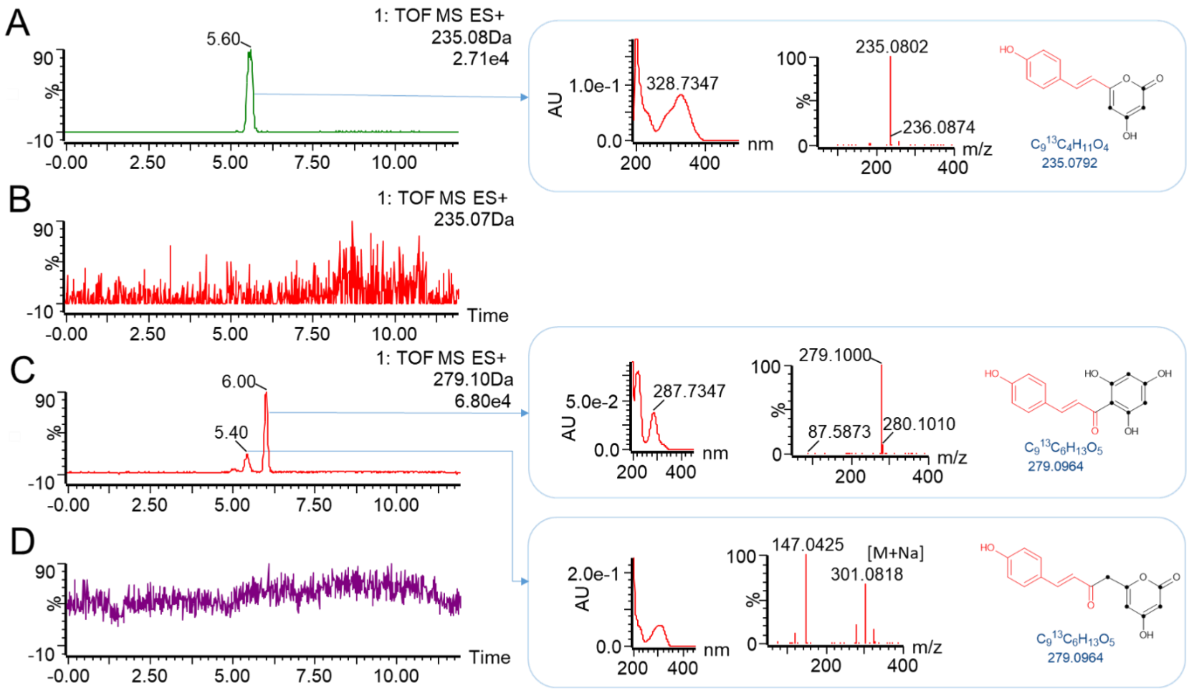

Supplementary Figure 29. High resolution TOF ESI-MS analysis of STO03G058250 (CHS) reaction mixture containing *p*-coumaroyl-CoA as a starter unit and $^{13}C_3$-malonyl-CoA as extender substrate. Extracted ion chromatogram for mass 235.07 Da in (A) reaction mixture and (B) reaction mixture containing only $^{13}C_3$-malonyl-CoA. EIC for mass 279.09 Da in (C) reaction mixture and (D) reaction mixture containing only $^{13}C_3$-malonyl-CoA. UV-VIS and ESI-MS spectrum, along with their possible structure of each peak, are shown with arrow. The malonyl derived structural part contains ^{13}C-carbon. The exact same retention time, UV spectrum, and increased mass confirmed production of bisnoryangonin, naringenin chalcone, and *p*-coumaroyl triacetic acid lactone (CTAL) in the CHS catalyzed reaction mixture.

Supplementary Table 1. Summary of whole genome sequence data generated in the project.

Platform	Library	Data (Gb)	Depth (x)	Q20 (%)	Q30 (%)	Accession No.
HiSeq	PE[†]_200bp-1	42.14	77.03	97.0	92.8	SRR11036019
	PE_200bp-2	44.51	81.36	96.9	92.4	SRR11036018
	PE_200bp-3	46.27	84.58	96.6	91.9	SRR11036017
	MP[†]_3kb-1	22.65	41.40	94.0	86.8	SRR11216106
	MP_3kb-2	24.38	44.56	93.2	85.5	SRR11216105
	MP_3kb-3	23.23	42.46	92.8	85.1	SRR11216104
	MP_5kb-1	25.46	46.54	93.4	86.0	SRR11216103
	MP_5kb-2	19.22	35.13	92.2	84.2	SRR11216112
	MP_5kb-3	20.40	37.29	92.1	84.0	SRR11216111
	MP_10kb-1	43.49	79.50	93.8	87.4	SRR11216114
	MP_10kb-2	43.17	78.91	94.4	88.6	SRR11216113
	MP_10kb-3	44.17	80.74	93.0	86.7	SRR11216110
	MP_20kb-1	45.56	83.28	92.3	85.8	SRR11216109
	MP_20kb-2	45.80	83.72	91.6	85.0	SRR11216108
	MP_20kb-3	44.49	81.33	92.5	86.7	SRR11216107
MiSeq	PE_500bp-1	13.90	25.41	89.7	80.4	SRR11212850
	PE_500bp-2	14.46	26.43	90.1	80.0	SRR11212849
	PE_500bp-3	14.63	26.74	91.0	80.9	SRR11212848
	Total	577.93	1056.41	--	--	--

[†] PE and MP represent pair-end and mate-pair, respectively.

Platform	Data (Gb)	Depth (x)	Average length (bp)	Accession No.
PacBio RS II system	40.88	74.73	12,275	SRR11128217
PacBio Sequel system	39.13	71.53	11,221	SRR11128212
				SRR11128213
				SRR11128214
				SRR11128215
				SRR11128216
Total	80.01	146.26	--	--

Supplementary Table 2. Summary statistics of the *S. tora* genome assemblies.

	**SOAPdenovo2**		**Allpaths-LG**		**Platanus**		**FALCON**
	Contigs	Scaffolds (1k over)	Contigs	Scaffolds (1k over)	Contigs	Scaffolds (1k over)	Contigs
**No**	47,840	1,270	28,597	5,323	16,941	4,550	957
**Length (bp)**	563,756,844	602,528,808	574,497,226	603,199,927	523,669,775	536,028,526	533,300,920
**N50 (bp)**	27,794	2,221,313	71,672	1,298,531	248,367	2,358,844	3,966,958
**Largest (bp)**	249,767	13,596,213	1,234,493	9,174,740	1,329,697	14,262,124	14,930,962
**Average (bp)**	11,784	474,432	20,089	113,319	30,911	117,808	557,263
**N (bp)**		30,295,731		28,700,086		12,358,751	--

Supplementary Table 3. BUSCO (v3.0.0) evaluation of the assembly from Platanus and FALCON assemblies.

Category	Platanus		FALCON	
(embryophyta_odb9)	Number	Percentage (%)	Number	Percentage (%)
Complete BUSCOs	1,305	90.6	1,358	94.3
Complete and single-copy BUSCOs	1,156	80.3	1,244	86.4
Complete and duplicated BUSCOs	149	10.3	114	7.9
Fragmented BUSCOs	66	4.6	16	1.1
Missing BUSCOs	69	4.8	66	4.6
Total BUSCO groups searched	1,440	100	1,440	100

Supplementary Table 4. Comparison of the assembled pseudochromosomes and 10 independently sequenced BACs.

No.	BAC ID	BAC length (bp)	Chr ID	Coverage (%)	Identities (%)
1	B050-B02	82,000	Chr3	100	99.94
2	B020-G17	92,904	Chr5	100	99.93
3	B016-D19	66,759	Chr6	100	99.95
4	H036-G09	81,063	Chr6	98.4	99.88
5	H001-O11	88,210	Chr8	100	99.49
6	H019-A05	87,111	Chr8	100	99.90
7	H024-N24	81,044	Chr8	100	99.81
8	H002-L14	107,545	Chr9	100	99.97
9	B011-O10	92,399	Chr10	100	99.79
10	H017-L06	87,235	Chr13	96	99.83

Supplementary Table 5. Functional annotations of protein-coding genes in *S. tora*.

	NO.	Percent (%)
**TOTAL**	45,268	
**NR**	31,010	68.50
**GO**	25,453	56.23
**KEGG**	17,450	38.55
**SWISSPROT**	23,533	51.99
**EGGNOG**	17,786	39.29
**NO HIT**	13,708	30.28

Supplementary Table 6. Annotation of rRNA and tRNA genes in *S. tora*.

Type		Copy	Average length (bp)	Total length (bp)	% of genome
rRNA	5S	432	119.13	51463	0.009
	5.8S	106	155.24	16455	0.003
	18S	107	1839.17	196791	0.036
	28S	107	3968.50	424629	0.078
tRNA		839	74.33	62363	0.011

Supplementary Table 7. Transcription factor genes in *S. tora* and 15 other plant species.

TF family	Species‡															
	SETOT	CHAFA	MIMPU	FALAL	ARHYP	MEDTR	CICAR	CICRE	PISSA	GLYMA	CAJCA	PHAVU	VIGRA	VIGAN	VIGUN	VITVI
**AP2/ERF**	169	159	151	167	209	210	157	140	192	341	184	179	184	187	195	148
**B3**	69	51	63	48	195	125	56	49	147	114	77	70	60	62	77	67
**BBR-BPC**	3	5	5	5	10	2	3	2	16	10	5	5	5	5	6	5
**BES1**	9	7	3	7	16	7	6	7	8	16	6	7	7	8	8	8
**bHLH**	137	130	131	149	225	159	128	104	271	300	166	154	158	154	149	125
**bZIP**	72	53	53	67	104	68	58	55	111	130	67	63	69	73	69	56
**C2C2**	102	86	86	101	135	102	86	80	114	180	98	94	91	92	95	68
**C2H2**	13	13	12	12	16	1	12	10	15	23	12	11	11	13	13	8
**C3H**	53	33	46	41	77	50	41	39	77	76	47	40	42	43	41	40
**CAMTA**	7	6	5	5	25	8	7	7	13	15	9	8	9	8	8	4
**CPP**	6	7	3	8	22	7	5	5	18	12	6	6	6	5	5	6
**DBB**	6	6	7	5	7	6	5	4	12	13	7	7	8	9	8	6
**E2F/DP**	7	6	6	7	17	6	6	8	11	14	7	7	7	8	7	7
**EIL**	9	18	5	5	14	10	7	6	10	14	6	7	7	4	7	4
**FAR1**	71	44	45	25	214	60	33	17	36	48	41	25	71	52	59	69
**GeBP**	7	4	5	6	11	6	8	6	7	9	6	5	7	5	5	6
**GRAS**	54	54	46	52	80	67	47	48	60	99	63	55	58	56	57	48
**GRF**	9	10	13	9	17	8	8	8	14	22	10	10	9	9	9	9
**Homeobox**	73	63	63	77	122	85	73	64	98	150	78	82	82	96	78	55
**HSF**	25	33	23	16	42	25	21	19	45	50	28	30	32	31	30	19
**LBD(AS2/LOB)**	57	50	63	51	92	61	49	35	51	87	54	50	47	50	47	48
**LFY**	2	1	1	1	4	1	1	1	1	2	1	1	1	1	1	1
**MADS**	127	63	80	60	134	140	83	52	179	157	81	79	67	69	77	78
**MYB**	270	219	253	241	467	288	222	162	330	506	287	270	265	258	260	245
**NAC**	103	91	88	94	174	96	77	64	120	173	94	90	93	101	105	85
**NF-X1**	2	3	1	2	3	3	3	3	3	4	3	2	2	2	2	2
**NF-Y**	10	7	7	10	19	8	8	7	16	20	13	9	8	9	8	6
**Nin-like**	11	11	10	9	29	11	9	9	18	26	14	12	11	10	12	9
**NZZ/SPL**	1	1	0	1	2	0	1	1	0	0	0	2	2	2	2	2
**S1Fa-like**	2	2	0	1	3	3	3	1	3	4	3	2	1	1	2	2
**SBP**	25	19	11	22	34	23	20	17	33	43	23	23	22	22	25	18
**SRS**	10	9	11	9	27	10	8	8	9	21	11	10	11	11	9	5
**TCP**	24	24	24	22	57	21	23	19	29	52	28	27	27	28	28	21
**Whirly**	2	3	1	2	8	3	3	3	4	7	3	3	3	5	3	2
**WRKY**	80	70	76	74	166	107	78	60	116	173	98	91	93	91	94	61
**ZF-HD**	17	42	21	18	24	18	17	15	18	43	20	19	18	18	18	17
**Total**	**1,644**	**1,403**	**1,418**	**1,429**	**2,801**	**1,805**	**1,372**	**1,135**	**2,205**	**2,954**	**1,656**	**1,555**	**1,594**	**1,598**	**1,619**	**1,360**

‡ represents *S. tora* (SETOT), *C. fasciculata* (CHAFA), *M. pudica* (MIMPU), *F. albida* (FAIAL), *A. hypogaea* (ARHYP), *M. truncatula* (MEDTR), *C. arientinum* (CICAR), *C. reticulatum* (CICRE), *P. sativum* (PISSA), *G. max* (GLYMA), *C. cajan* (CAJCA), *P. vulgaris* (PHAVU), *V. radiata* (VIGRA), *V. angularis* (VIGAN), *V. unguiculata* (VIGUN), *V. vinifera* (VITVI)

Supplementary Table 8. Statistics of orthologs and paralogs in Fabaceae and *Vitis vinifera*.

Species	Genome size (Mbp)	No. coding genes	No. ortholog genes	No. paralog genes	No. species-specific genes	No. uncertain genes	No. non-species-specific genes	Species-specific genes (%)	P-value	Source of the data
Senna tora	**526.40**	**45,268**	**23,461**	**8,938**	**7,231**	**5,638**	**38,037**	**15.97**	**< 0.00001**	
Chamaecrista fasciculata	429.27	32,832	24,314	3,275	388	4,855	32,444	1.18	< 0.00001	ftp://parrot.genomics.cn/gigadb/pub/10.5524/101001_102000/101045/
Mimosa pudica	557.21	33,108	24,883	3,264	304	4,657	32,804	0.92	< 0.00001	ftp://parrot.genomics.cn/gigadb/pub/10.5524/101001_102000/101049/
Faidherbia albida	653.73	28,979	21,624	818	1,040	5,497	27,939	3.59	< 0.00001	https://bioinformatics.psb.ugent.be/orcae/aocc/overview/Faial/
Arachis hypogaea	2539.16	83,709	44,585	27,369	1,202	10,553	82,507	1.44		http://peanutgr.fafu.edu.cn/index.php
Medicago truncatula	412.92	41,939	29,619	7,200	4,165	9,460	46,279	9.93		GCF_000219495.3
Cicer arientinum	530.89	35,754	23,044	400	36	1,482	24,926	0.10		GCF_000331145.1
Cicer reticulatum	416.66	26,404	21,425	1,514	573	2,892	25,831	2.17		ftp://ftp.bioinfo.wsu.edu/species/Cicer_reticulatum/C.reticulatum_PI489777_v1/
Pisum sativum	3920.16	57,835	35,155	11,388	4,447	6,845	53,388	7.69		https://urgi.versailles.inra.fr/download/pea/
Glycine max	979.05	71,219	41,070	1,110	137	4,477	46,657	0.19		GCF_000004515.5
Cajanus cajan	592.97	41,387	25,788	899	148	2,284	28,971	0.36		GCF_000340665.1
Phaseolus vulgaris	521.08	32,720	24,899	374	579	2,282	27,555	1.77		GCF_000499845.1
Vigna radiata	463.64	42,284	24,438	439	123	1,961	26,838	0.29		GCF_000741045.1
Vigna angularis	467.30	37,769	24,491	305	85	1,753	26,549	0.23		GCF_001190045.1
Vigna unguiculata	519.07	41,173	26,063	749	117	1,301	28,113	0.28		GCF_004118075.1
Vitis vinifera	486.20	41,208	21,064	2,002	140	2,470	28,971	0.36		GCF_000003745.3

Supplementary Table 9. Metabolic domain enrichment of expanded gene families in *S. tora*.

Metabolic domain	Unique genes	-log10(p.value)
Phenolic specialized metabolism	126	2.330
Cofactors metabolism	20	2.103
Carbohydrates metabolism	15	1.578
Hormones metabolism	9	1.579
Nitrogen-containing specialized metabolism	9	1.579

Supplementary Table 10. A summary of the 16 CHS-L genes in *S. tora*.

Number	Gene ID	Size (CDS)	Size (aa)	Mw (Da)	pI	No. of Exon	No. of Intron
1	Sto02g027180 (CHS-L1)	1149	382	41950.02	6.01	2	1
2	Sto07g228150 (CHS-L2)	1173	390	42634.98	6.36	2	1
3	Sto07g228160 (CHS-L3)	1173	390	42793.11	6.28	2	1
4	Sto07g228180 (CHS-L4)	1182	393	43436.73	6.50	2	1
5	Sto07g228210 (CHS-L5)	1185	394	43471.72	6.40	2	1
6	Sto07g228220 (CHS-L6)	1185	394	43488.73	6.02	2	1
7	Sto07g228230 (CHS-L7)	1173	390	41798.01	6.21	2	1
8	Sto07g228240 (CHS-L8)	1278	425	46806.84	6.74	2	1
9	Sto07g228250 (CHS-L9)	1173	390	42792.12	6.32	2	1
10	Sto07g228260 (CHS-L10)	1167	388	42664.03	6.32	2	1
11	Sto07g228270 (CHS-L11)	1173	390	42915.26	6.44	2	1
12	Sto07g228300 (CHS-L12)	1173	390	42879.21	6.43	2	1
13	Sto07g228310 (CHS-L13)	1170	389	42799.18	6.40	2	1
14	Sto07g228320 (CHS-L14)	1170	389	42750.11	6.40	2	1
15	Sto07g228370 (CHS-L15)	1194	397	43748.55	6.47	2	1
16	Sto07g228380 (CHS-L16)	1188	395	43358.92	6.83	2	1

Supplementary Table 11. List of statistically significant expanded/contracted CHS-L and CHS subfamilies in *S. tora* and 15 related species.

Subfamilies		Species‡															
		SETOT	CHAFA	MIMPU	FAIAL	ARHYP	MEDTR	CICAR	CICRE	PISSA	GLYMA	CAJCA	PHAVU	VIGRA	VIGAN	VIGUN	VITVI
CHS-L	E/C*	Rapid_E†	E	-	-	E	-	-	-	C	-	C	-	-	-	-	C
	Gain/Loss gene count	11	-	-	-	1	-	-	-	-1	-	-1	-	-	-	-	-1
	Orthologous gene	16	5	0	0	2	1	0	0	0	1	0	0	0	0	0	0
CHS	E/C*	C	E	C	C	Rapid_E†	Rapid_E†	-	-	Rapid_C†	E	C	C	-	C	Rapid_E†	Rapid_E†
	Gain/Loss gene count	-3	7	-1	-5	32	11	-	-	-10	4	-2	-2	-	-1	7	17
	Orthologous gene	12	22	11	7	48	21	6	6	0	15	9	8	8	7	17	39

* E/C represents expansion/contraction. † Rapid_E and Rapid_C indicate rapid expansion and rapid contraction (see Methods). ‡ represents *S. tora* (SETOT), *C. fasciculata* (CHAFA), *M. pudica* (MIMPU), *F. albida* (FAIAL), *A. hypogaea* (ARHYP), *M. truncatula* (MEDTR), *C. arientinum* (CICAR), *C. reticulatum* (CICRE), *P. sativum* (PISSA), *G. max* (GLYMA), *C. cajan* (CAJCA), *P. vulgaris* (PHAVU), *V. radiata* (VIGRA), *V. angularis* (VIGAN), *V. unguiculata* (VIGUN), *V. vinifera* (VITVI).

Supplementary Table 12. List of anthraquinone standards used in this study.

No.	Name	Formula	Average MS(Da)	Monoisotopic MS(Da)	$[M+H]^+$	$[M-H]^-$	Production for MRM
1	Glucoaurantio-obtusin	$C_{23}H_{24}O_{12}$	492.436	492.127	493.1341	491.1195	242.1
2	Obtusin	$C_{18}H_{16}O_7$	344.321	344.09	345.0969	343.08233	313
3	Chryso-obtusin	$C_{19}H_{18}O_7$	358.348	358.105	359.1125	357.09798	342.1
4	Chrysophanol	$C_{15}H_{10}O_4$	254.242	254.058	255.0652	253.05063	225.1
5	Emodin	$C_{15}H_{10}O_5$	270.241	270.053	271.0601	269.04555	225.1
6	Gluco-obtusifolin	$C_{22}H_{22}O_{10}$	446.41	446.121	447.1286	445.11402	268.2
7	Aurantio-obtusin	$C_{17}H_{14}O_7$	330.294	330.074	331.0812	329.06668	298.9
8	Aloe-emodin	$C_{15}H_{10}O_5$	270.241	270.053	271.0601	269.04555	240.1
9	Physcion	$C_{16}H_{12}O_5$	284.268	284.068	285.0758	283.0612	239.9
10	Obtusinfolin	$C_{16}H_{12}O_5$	284.268	284.068	285.0758	283.0612	92.1

Supplementary Table 13. Contents of ten anthraquinone compounds at different stages of *S. tora* seed development (Stage1-Stage7).

Compounds	Amount of anthraquinones (μg/g)*						
	**Stage1**	**Stage2**	**Stage3**	**Stage4**	**Stage5**	**Stage6**	**Stage7**
Glucoaurantio-obtusin	N.D	4.03±0.30	35.45±1.48	224. 80±20.80	1009.73±66.67	1144.47±24.91	296.00±11.01
Obtusin	N.D	N.D	N.D	N.D	N.D	N.D	68.22 ±8.00
Chryso-obtusin	N.D	N.D	N.D	0.82±0.18	1.76±0.58	1.77±0.49	54.66±8.03
Chrysophanol	3.24±0.05	13.98±0.65	18.23±1.67	19.69±1.89	46.23±2.25	30.21±1.63	12.89±0.98
Emodin	215.5±12.51	74.39±9.64	47.34±4.32	13.68±0.49	13.27±1.19	4.13±1.24	5.52±1.21
Gluco-obtusifolin	N.D	N.D	3.07±1.14	31.16±1.21	193.04±12.45	224.13±4.92	56.78±3.79
Aurantio-obtusin	N.D	N.D	N.D	0.57±0.14	3.57±0.41	4.94±0.76	312.20±73.63
Aloe-emodin	N.D	N.D	N.D	N.D	1.47±0.19	1.79±0.40	0.93±0.19
Physcion	6.84±0.18	10.19±0.72	8.21±0.71	4.16±0.32	5.97±1.14	3.55±0.34	1.81±0.25
Obtusifolin	N.D	0.13±0.02	0.20±0.06	1.10±0.13	1.04±0.08	1.22±0.04	84.45±3.27
**Total**	**225.58±12.74**	**102.72±11.33**	**112.5±9.38**	**295.98±25.16**	**1,276.08±84.96**	**1,416.21±34.73**	**893.46±110.36**

* indicates mean of three biological replicate experiments. Source data are provided as a Source Data file.

Supplementary Table 14. Significantly enriched molecular function GO categories of the gene expression cluster 6 during seed development.

GO ID	GO description	Number of genes	FDR-corrected p-value
GO:0016758	transferase activity, transferring hexosyl groups	39	0.00001
GO:0010427	abscisic acid binding	10	0.00142
GO:0008194	UDP-glycosyltransferase activity	20	0.00514
GO:0051536	iron-sulfur cluster binding	15	0.01000
GO:0004864	protein phosphatase inhibitor activity	9	0.01000
GO:0038023	signaling receptor activity	9	0.01000
GO:0000978	RNA polymerase II proximal promoter sequence-specific DNA binding	8	0.01362
GO:0004842	ubiquitin-protein transferase activity	39	0.03018
GO:0010295	(+)-abscisic acid 8'-hydroxylase activity	4	0.03018
GO:0036402	proteasome-activating ATPase activity	4	0.03018
GO:0047216	inositol 3-alpha-galactosyltransferase activity	3	0.03472
GO:0016705	oxidoreductase activity, acting on paired donors, with incorporation or reduction of molecular oxygen	37	0.04860

Supplementary Table 15. Expression analysis of CHS-L genes during seed development.

Gene_ID	Gene Expression Value (TPM)						
	S1	S2	S3	S4	S5	S6	S7
Sto07g228380 (CHS-L16)	0	0	0	0	0	0	0
Sto02g027180 (CHS-L1)	0	0	0.06	0	0	0.11	0.05
Sto07g228370 (CHS-L15)	0.24	0	0	0	0	0	0
Sto07g228310 (CHS-L13)	2096.42	569.03	214.12	425.35	1.535	0.52	1.98
Sto07g228240 (CHS-L8)	475.80	429.96	37.77	1.72	0	0.11	1.01
Sto07g228160 (CHS-L3)	1618.38	655.23	25.51	7.15	0.06	0.06	0
Sto07g228180 (CHS-L4)	7.93	4.735	0.205	0.16	0	0	0
Sto07g228150 (CHS-L2)	5324.20	3288.64	1248.77	1575.40	21.33	0.19	1.08
Sto07g228270 (CHS-L11)	1432.91	613.92	182.52	89.99	0.34	0.87	5.25
Sto07g228260 (CHS-L10)	3246.98	1451.94	325.57	295.97	1.01	0.34	3.14
Sto07g228320 (CHS-L14)	119.29	125.24	154.36	104.18	0.59	1.67	11.84
Sto07g228300 (CHS-L12)	59.63	46.03	53.13	22.61	0.84	1.42	4.57
Sto07g228230 (CHS-L7)	263	186.74	187.37	106.19	1.28	1.76	11.46
Sto07g228210 (CHS-L5)	10.91	350.99	463.93	281.29	0.56	0.06	0
Sto07g228250 (CHS-L9)	1055.83	1053.35	1274.55	2696.51	23.22	2.04	5.40
Sto07g228220 (CHS-L6)	3.54	1289.72	3110.70	5017.52	32.58	0.87	20.37

Supplementary Table 16. Mapping statistics of Illumina, RNA-Seq, and Iso-Seq data in this study.

	Library	No. sequencing reads	No. high quality reads	Mapping rate (%)
Genome-Seq	PE_200-1	279,095,332	185,792,369	99.75
	PE_200-2	294,819,090	205,902,564	99.75
	PE_200-3	306,485,872	213,085,841	99.74
RNA-Seq	Seed	22,986,190	22,122,628	84.92
	Flower	69,614,064	54,230,644	85.15
	Leaf	45,662,632	39,833,132	73.21
	Stem	66,562,030	52,162,636	84.45
	root	46,386,464	40,201,556	89.62
	Stage1-1 (S[†])	44,230,592	39,568,687	94.05
	Stage1-2 (S)	41,572,424	37,710,345	94.83
	Stage2-1 (S)	40,494,100	34,912,700	93.32
	Stage2-2 (S)	38,637,340	34,932,019	89.88
	Stage3-1 (S)	44,752,688	40,510,133	95.49
	Stage3-2 (S)	39,833,596	36,001,604	92.36
	Stage4-1 (S)	44,470,644	40,165,885	95.50
	Stage4-2 (S)	37,584,824	34,093,193	93.71
	Stage5-1 (S)	38,394,282	34,885,044	95.16
	Stage5-2 (S)	42,445,844	38,388,021	83.86
	Stage6-1 (S)	38,629,298	34,615,713	95.46
	Stage6-2 (S)	41,408,918	37,541,325	94.69
	Stage7-1 (S)	38,492,940	35,313,423	87.78
	Stage7-2 (S)	38,251,900	35,065,516	85.39
Iso-Seq	Consensus seq.	768,745	118,390	97.18

[†]S indicates seed.

Supplementary Table 17. **Summary of *S. tora* genetic map and anchored contigs.**

LG	No. of markers	Genetics length (cM)	No. of contig	Physical length (bp)	Anchored contig list
1	235	167.2	13	30,974,193	c102, c129, c14, c150, c152, c174, c175, c23, c60, c61, c64, c76, c78
2	280	171.89	9	29,371,314	c154, c256, c36, c37, c41, c44, c48, c68, c97
3	358	251.46	7	29,263,208	c103, c119, c15, c34, c40, c73, c84
4	226	119.36	6	24,463,614	c30, c52, c56, c6, c82, c87
5	417	312.65	10	35,412,120	c117, c12, c125, c2, c217, c26, c43, c59, c7, c95
6	487	259.11	10	45,180,853	c1, c124, c13, c161, c17, c38, c46, c62, c79, c83
7	257	137.4	9	22,430,049	c109, c11, c177, c29, c35, c45, c58, c69, c98
8	508	347.47	7	37,916,848	c21, c28, c32, c33, c42, c5, c63
9	426	174.66	4	34,240,060	c39, c3-1, c3-2, c85
10	279	204.49	10	32,156,945	c100, c107, c130, c137, c143, c16, c20, c31, c8, c81
11	263	141.72	6	23,588,853	c108, c149, c18, c24, c4, c9
12	281	148.82	11	19,016,816	c145, c173, c184, c25, c27, c273, c54, c70, c74, c75, c99
13	438	343.8	9	37,054,565	c10, c123, c163, c19, c22, c49, c53, c80, c86
Total	4,455	2,780.03	111	401,069,438	

Supplementary Table 18. Statistics of the assembled 13 chromosomes of *S. tora*.

Chromosome	Anchored contig number	Length of chromosome (bp)	N (%)
Chr1	21	32,816,166	0.01
Chr2	35	42,009,719	0.01
Chr3	17	37,860,065	0.00
Chr4	39	30,689,712	0.01
Chr5	26	52,777,034	0.00
Chr6	14	46,512,068	0.00
Chr7	17	30,975,534	0.01
Chr8	30	49,705,205	0.01
Chr9	7	35,860,388	0.00
Chr10	21	41,499,577	0.00
Chr11	22	30,871,617	0.01
Chr12	28	29,799,933	0.01
Chr13	13	41,270,226	0.00
Chr00 (Unlocalized)	431	23,709,733	0.01
**Total**	721	526,356,977	0.01

Supplementary Table 19. Statistics of repeat elements in the *S. tora* genome.

Types	Counts	Masked length	Masked (%)
**Retroelements**	**146,719**	**126,200,327**	**23.97**
SINEs:	3,648	320,822	0.06
ALUs	258	32,943	0.01
LINEs:	36,028	17,305,497	3.29
CR1	33	1,657	0.00
L1	10,974	4,629,733	0.88
L2	8,913	9,617,265	1.83
RTE-BovB	16,036	3,043,940	0.58
Penelope	61	11,083	0.00
LTR elements:	106,503	108,574,008	20.63
Ty1/Copia	36,468	24,658,248	4.68
Ty3/Gypsy	67,989	81,902,798	15.56
BEL/Pao	594	732,594	0.14
Caulimoviruses	554	1,034,136	0.20
**DNA transposons**	**126,655**	**34,116,808**	**6.48**
MULE-MuDR	37,089	8,340,270	1.58
CMC-EnSpm	17,286	8,174,915	1.55
hAT-Ac	26,300	5,529,442	1.05
hAT-Tag1	11,009	2,970,951	0.56
hAT-Charlie	3,450	2,231,696	0.42
Unclassified:	325,176	111,279,209	21.14
**Total interspersed repeats:**		**261,871,205**	**49.75**
Small RNA:	2,030	377,643	0.07
Satellites:	72	21,056	0.00
Simple repeats:	191,675	13,232,686	2.51
**Total**		**283,551,945**	**53.87**

Supplementary Table 20. Statistics of the RNA-Seq reads produced by Illumina sequencing platform.

Stage	Sample	Read bases	Reads	GC (%)	Q20 (%)	Q30 (%)	Accession No.
1	Stage1-1	5,716,321,575	44,230,592	44.33	89.46	77.52	SRR11050224
	Stage1-2	5,416,922,426	41,572,424	44.31	90.71	79.20	SRR11050223
2	Stage2-1	5,222,538,241	40,494,100	45.15	90.36	79.00	SRR11050213
	Stage2-2	4,957,068,902	38,637,340	43.89	90.41	78.57	SRR11050212
3	Stage3-1	5,849,017,608	44,752,688	45.23	90.52	78.95	SRR11050211
	Stage3-2	5,182,252,082	39,833,596	45.52	90.38	78.70	SRR11050210
4	Stage4-1	5,794,527,131	44,470,644	46.98	90.32	79.02	SRR11050209
	Stage4-2	4,900,442,069	37,584,824	47.75	90.71	79.78	SRR11050208
5	Stage5-1	5,022,226,735	38,394,282	47.26	90.86	79.96	SRR11050207
	Stage5-2	5,555,427,803	42,445,844	48.23	90.44	79.33	SRR11050206
6	Stage6-1	5,171,309,937	38,629,298	45.82	89.61	78.35	SRR11050222
	Stage6-2	5,547,031,298	41,408,918	46.55	90.66	78.80	SRR11050221
7	Stage7-1	5,193,556,843	38,492,940	47.35	91.74	82.09	SRR11050220
	Stage7-2	5,148,850,942	38,251,900	47.13	91.67	82.09	SRR11050219

Supplementary Table 21. Primers used for the study of CHS-L9 (STO07G228250) and CHS (STO03G058250) genes.

Names	Used for	Sequences* (5′-3′)
Sto07g228250F	Cloning	AAGGATCCATGGAGAGTGCTGGAG
Sto07g228250R	Cloning	AACTCGAGCTAGTCTCTCAGAGGG
Sto03g058250F	Cloning	GAATTCATGGTGAGTGTGAGTGAGATC
Sto03g058250R	Cloning	AAGCTTTTAGTTAACTCCCACACTGCG

*Underlined sequences represent restriction enzyme sites.

Supplementary References

1. Jones, D.T., Taylor, W.R. & Thornton, J.M. The rapid generation of mutation data matrices from protein sequences. *Bioinformatics* **8**, 275-282 (1992).
2. Kumar, S., Stecher, G., Li, M., Knyaz, C. & Tamura, K. MEGA X: molecular evolutionary genetics analysis across computing platforms. *Mol Biol Evol* **35**, 1547-1549 (2018).
