# Supplementary material for: Genome-enabled discovery of anthraquinone biosynthesis in Senna tora
Source: Nat Commun. 2020 Nov 18;11:5875. doi: 10.1038/s41467-020-19681-1 (PMC7674472; doi:10.1038/s41467-020-19681-1)
Supplement: Supplementary file 3 — Reporting Summary [file 41467_2020_19681_MOESM3_ESM.pdf]

nature research

Corresponding author(s): Sang-Ho Kang
Last updated by author(s): Oct 5, 2020

Reporting Summary

Nature Research wishes to improve the reproducibility of the work that we publish. This form provides structure for consistency and transparency in reporting. For further information on Nature Research policies, see our Editorial Policies and the Editorial Policy Checklist.

Statistics

For all statistical analyses, confirm that the following items are present in the figure legend, table legend, main text, or Methods section.

n/a	Confirmed	
☐	☒	The exact sample size (*n*) for each experimental group/condition, given as a discrete number and unit of measurement
☐	☒	A statement on whether measurements were taken from distinct samples or whether the same sample was measured repeatedly
☒	☐	The statistical test(s) used AND whether they are one- or two-sided<br>*Only common tests should be described solely by name; describe more complex techniques in the Methods section.*
☒	☐	A description of all covariates tested
☐	☒	A description of any assumptions or corrections, such as tests of normality and adjustment for multiple comparisons
☐	☒	A full description of the statistical parameters including central tendency (e.g. means) or other basic estimates (e.g. regression coefficient) AND variation (e.g. standard deviation) or associated estimates of uncertainty (e.g. confidence intervals)
☐	☒	For null hypothesis testing, the test statistic (e.g. *F*, *t*, *r*) with confidence intervals, effect sizes, degrees of freedom and *P* value noted<br>*Give P values as exact values whenever suitable.*
☒	☐	For Bayesian analysis, information on the choice of priors and Markov chain Monte Carlo settings
☒	☐	For hierarchical and complex designs, identification of the appropriate level for tests and full reporting of outcomes
☒	☐	Estimates of effect sizes (e.g. Cohen's *d*, Pearson's *r*), indicating how they were calculated

Our web collection on statistics for biologists contains articles on many of the points above.

Software and code

Policy information about availability of computer code

Data collection	SOAPdenovo v2.04, ALLpaths-LG v48777, Platanus v1.2.1, SSPACE v3.0, FALCON v0.4, Arrow v2.1.0, BWA, and GATK were used in genome assembly. Hi-C data was assembled into super-scaffold using LACHESIS and Juicebox. Newbler v2.8 was used in BAC assembly.
Data analysis	BUSCO v3.0.0 and BLAT v3.2.4 were used in genome assessment. QTL IciMapping v4.1 was used in linkage map analysis. Trimmomatic v0.36 was used for quality control of both genome-Seq and RNAseq sequences. CLCMapper v4.2.0 was used for genome-Seq to remove contaminants and Jellyfish v2.0 estimated the genome size. TRF (Tandem Repeats Finder) v4.07b, RepeatMasker v4.0.5, RepeatModeler v1.0.8, RepeatScout v1.0.5, RMBlastn v2.2.27+, Tophat v2.1.1, Cufflinks v2.2.1, PASA v2.4.1, AUGUSTUS v3.0.3, GENEID v1.4, Exonerate v2.2.0, BLASTP v2.2.27+, InterProScan v5.36-75.0, PlantTFDB v5.0 database (http://planttfdb.cbi.pku.edu.cn/) were used in genome annotation. OrthoMCL v2.0.9, MAFFT v7.305b, Gblocks v0.91b, IQ-Tree v1.5.0-beta, CAFE v3.1, BEAST2. ParaAT v2.0 was used in phylogenetic tree construction, phylogenomic dating and genome duplication. edgeR v3.22.5 was used for expression analysis. MEGA v7.0, MEGA vX, and MUSCLE were used in phylogenetic tree construction of CHS and CHS-L genes. E2P2 v4.0, RPSD v4.2, and Pathway Tools v23.5 were used to predict enzymes and metabolic pathways in S. tora. In-house Python and R scripts for gene prediction and Ks distribution plot and heatmap analyses can be downloaded at https://github.com/MyungheeJung/Senna_tora.git.

For manuscripts utilizing custom algorithms or software that are central to the research but not yet described in published literature, software must be made available to editors and reviewers. We strongly encourage code deposition in a community repository (e.g. GitHub). See the Nature Research guidelines for submitting code & software for further information.

Data

Policy information about availability of data

All manuscripts must include a data availability statement. This statement should provide the following information, where applicable:

- Accession codes, unique identifiers, or web links for publicly available datasets
- A list of figures that have associated raw data
- A description of any restrictions on data availability

All the raw sequences are deposited to NCBI SRA under the BioProject PRJNA605066 (https://www.ncbi.nlm.nih.gov/bioproject/PRJNA605066). The genome assemblies and annotation files are deposited in GenBank under the accession number JAAIUW000000000 (https://www.ncbi.nlm.nih.gov/nuccore/JAAIUW000000000). The S. tora genome is also available at http://nabic.rda.go.kr/Species/Senna_tora2.

Field-specific reporting

Please select the one below that is the best fit for your research. If you are not sure, read the appropriate sections before making your selection.

- [x] Life sciences
- [ ] Behavioural & social sciences
- [ ] Ecological, evolutionary & environmental sciences

For a reference copy of the document with all sections, see nature.com/documents/nr-reporting-summary-flat.pdf

Life sciences study design

All studies must disclose on these points even when the disclosure is negative.

Sample size	We produced genome data for Senna tora with 200 and 500 bp PE, 3, 5, 10, 20 and 20 kb MP, and PacBio RS II and Sequel systems. We also produced BAC clones and GBS libraries.
Data exclusions	No data exclusions in this manuscript.
Replication	For the accuracy of gene prediction, RNA-seq from five tissues (leaf, root, stem, flower, and dry seed) were done with one biological replicate (These data were used only in the gene prediction, so no replication was needed in this study). The RNA-seq experiments from seven developmental stages of seeds were done with two biological replicates. Primary and secondary metabolites assays were performed in triplicates. All enzyme assays were performed in triplicates.
Randomization	No randomization in this manuscript because no randomization was needed in this study (genome sequencing, annotation and biochemical assays).
Blinding	No blinding in this manuscript because the nature of our work described in the paper (genome sequencing, annotation and biochemical assays) does not necessitate blinded experiments.

Reporting for specific materials, systems and methods

We require information from authors about some types of materials, experimental systems and methods used in many studies. Here, indicate whether each material, system or method listed is relevant to your study. If you are not sure if a list item applies to your research, read the appropriate section before selecting a response.

Materials & experimental systems

n/a	Involved in the study	
x		Antibodies
x		Eukaryotic cell lines
x		Palaeontology and archaeology
x		Animals and other organisms
x		Human research participants
x		Clinical data
x		Dual use research of concern

Methods

n/a	Involved in the study	
x		ChIP-seq
x		Flow cytometry
x		MRI-based neuroimaging
