# Supplementary material for: Genome-enabled discovery of anthraquinone biosynthesis in Senna tora
Source: Nat Commun. 2020 Nov 18;11:5875. doi: 10.1038/s41467-020-19681-1 (PMC7674472; doi:10.1038/s41467-020-19681-1)
Supplement: Supplementary file 4 — Description of Additional Supplementary Files [file 41467_2020_19681_MOESM4_ESM.docx]

**Description of Additional Supplementary Files**

Supplementary Data 1

Annotation of long non-coding RNA (lncRNA) genes in the genome *S. tora.*

Supplementary Data 2

All significantly enriched biological process GO and KEGG categories of expanded gene families in S. tora compared to other 15 species (*C. fasciculata*, *M. pudica*, *F. albida*, *A. hpogaea*, *M. truncatula*, *C. arientinum*, *C. reticulatum*, *P. Sativum*, *G. max*, *C. cajan*, *P. vulgaris*, *V. radiata*, *V. angularis*, *V. unguiculata*, and *V. vinifera*).

Supplementary Data 3

Genes and metabolic domain enrichment of families that expanded specifically or rapidly+specifically expanded in *S. tora* or families that expanded / rapidly expanded in *S. tora* and other species.

Supplementary Data 4

Expression and metabolic domains of all genes predicted to catalyse small molecule metabolism in StoraCyc.

Supplementary Data 5

178 putative metabolites from seven different seed development in *S. tora*.

Supplementary Data 6

Quantitative estimation of 69 primary metabolites during seed development in *S. tora*.

Supplementary Data 7

Metabolic domain enrichment among genes in coexpression clusters 3 and 6.

Supplementary Data 8

Final linkage map including the linkage group (LG), SNP marker, and cM position.

Supplementary Data 9

Expression value for each gene (TPM > 0.0) from seven different seed development in *S. tora*.
