# Supplementary figures and images for: Genome-enabled discovery of anthraquinone biosynthesis in Senna tora
Source: Nat Commun. 2020 Nov 18;11:5875. doi: 10.1038/s41467-020-19681-1 (PMC7674472; doi:10.1038/s41467-020-19681-1)

Original file for Supplementary Fig. 19A :


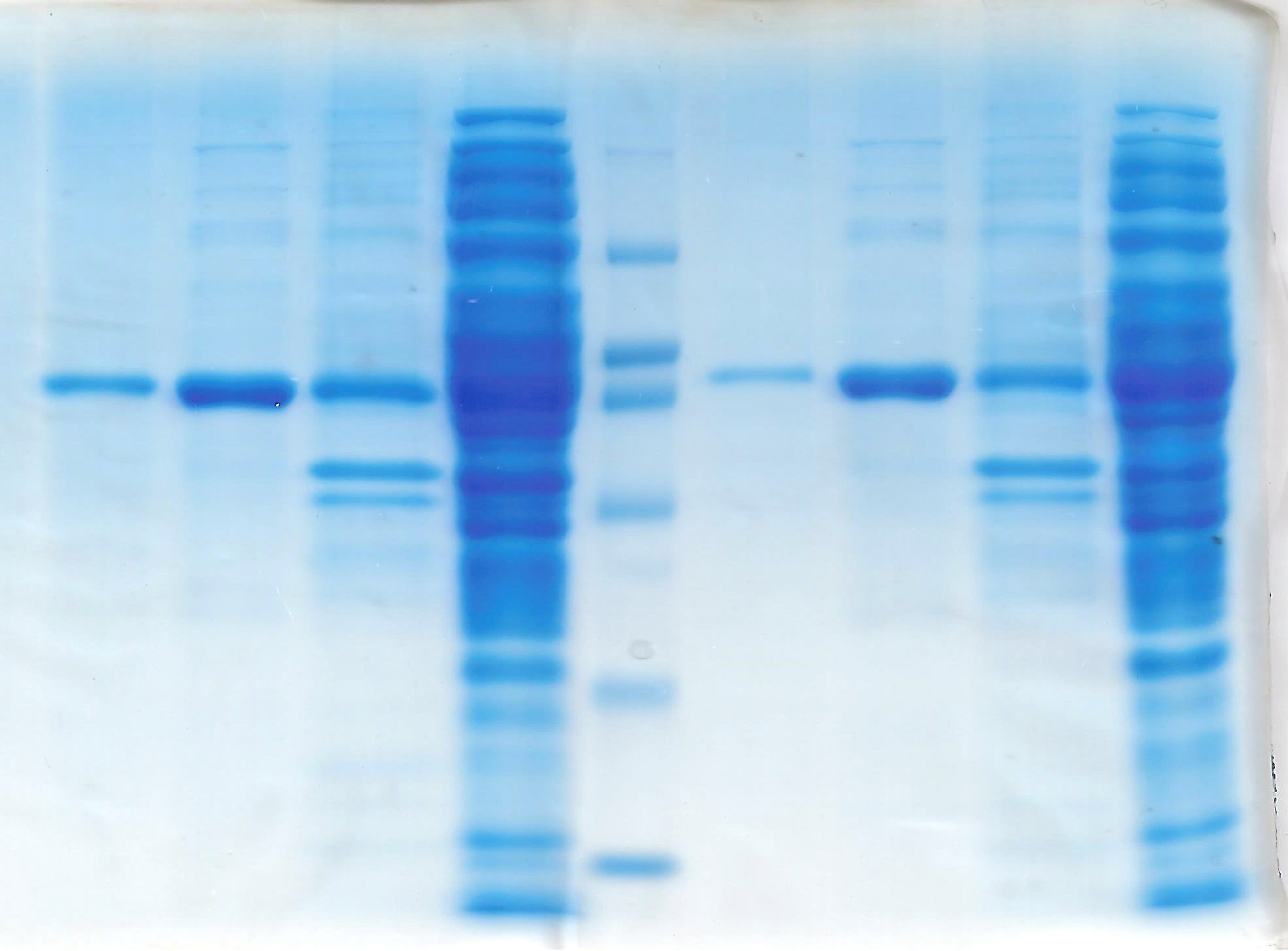


Original file for Supplementary Fig. 19B :


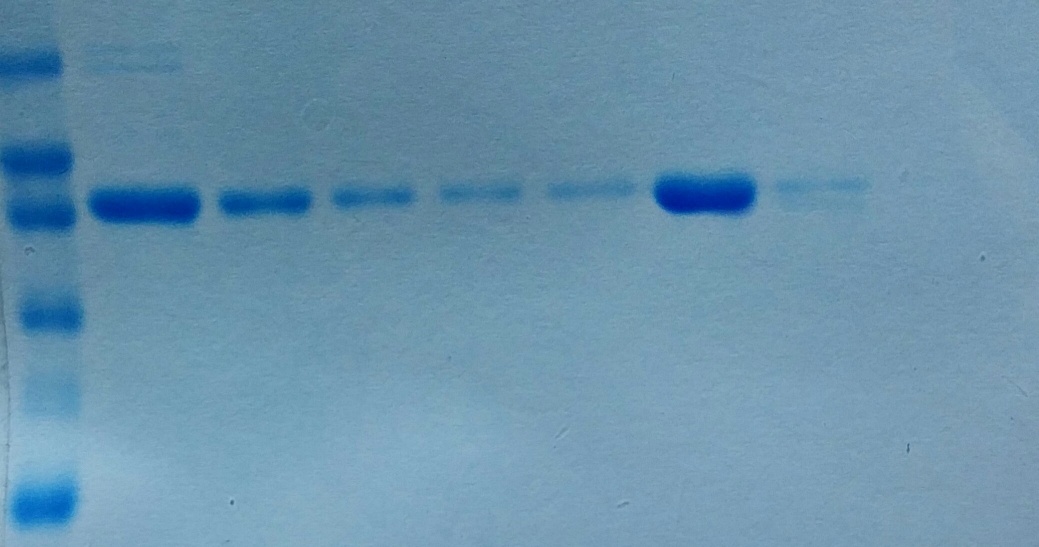

Supplement: Supplementary file 14 — Source Data [file 41467_2020_19681_MOESM14_ESM.zip › Source Data for Supplementary Figure 19.docx]
