# Supplementary material for: Genome-enabled discovery of anthraquinone biosynthesis in Senna tora
Source: Nat Commun. 2020 Nov 18;11:5875. doi: 10.1038/s41467-020-19681-1 (PMC7674472; doi:10.1038/s41467-020-19681-1)
Supplement: Supplementary file 2 — Peer Review [file 41467_2020_19681_MOESM2_ESM.pdf]

Reviewer #1 (Remarks to the Author):

The manuscript illustrates very well how genomics and data integration can accelerate the fine characterization of a biological process, the biosynthesis of anthraquinones. The manuscript reports the assembly and the annotation of the S. tora genome. The identification of gene families over-represented in the genome allowed the identification of a ~150kb region containing 15 genes coding for probable CHS-L. The integrated analysis of metabolomic and transcriptomic dynamics during the development of the seed allowed a fine characterization of the family members. The best candidate was selected thanks to an approach integrating phylogeny and established knowledge. Its enzymatic activity during the biosynthesis of anthraquinones has been validated thanks to convincing results obtained with an experimental design very well described.

Major comments:

A) Data availability
- raw sequencing data: please provide the NCBI SRA accession numbers for all sequence datasets generated in the projet: illumina (WGS, HiC, RNASeq GBS) and pacbio datasets. Adding missing datasets to Table S1 and adding a new column with accession numbers to S1 and S21 Tables will link the raw data to the minimum set of metadata ensuring re-usability.
- genome: please provide the Genbank accession number for the annotated reference genome
- genetic map: please provide a table corresponding to the finalized genetic map (context sequences of markers, genetic position on linkage groups)
- RNAseq data: The authors provide Tables for metabolic data (Tables S15 and S16) but the RNAseq counterpart is missing. Please provide the full expression matrix (raw count and TPM for all gene models). The expression matrix would be very useful to evaluate the data underlying the Fig2E heatmap. The members of the CHS-L cluster are certainly highly similar, some readers would appreciate to find the raw counts assigned to each member.

B) Building of pseudo chromosomes / reference genome
The paragraph describing the genome generation is the most ambiguous part of the manuscript (L58-L72).
Firstly, it describes the sequencing project (combination of illumina and pacbio approaches) more than the final result. Indeed, from main text only, readers can falsely understand that several assemblies, generated using various technologies, were integrated.
I would suggest rewriting in order to focus on the result: a pacbio-based assembly performed with falcon, scaffolded with HiC; two rounds of consensus polishing (1: arrow/pacbio; 2: see below my questions on the illumina polishing). Illumina data (reads and assemblies) as well as 454/Illumina BAC assemblies were used to assess the completeness of the reference genome assembly. BUSCO to assess the quality of the gene models.
The second ambiguity is related to the use of the genetic map. Usually, in plant genome projects, the genetic map is used for the final scaffolding of pseudomolecules and/or to orientate and number the pseudochromosomes. Here, pseudo chromosomes were built from HiC. It is acceptable but some breakpoints are visible in the HiC results (Fig S3 and S4) and the structure of the genome must be confirmed by the genetic map. Unfortunately, the Fig S7 does not support the claim "the 13 linkage grouped matched well to the 13 chromosomes". Indeed, the figure shows local matches but the positions on the chromosomes are not reported making impossible the assessment of the collinearity at a global level. In addition, the Figure is designed to report only matches on the expected chromosome.
To confirm that the genetic map is consistent with the structure of the chromosomes, please provide a figure where both coordinate systems are reported as well as all significant matches.
C) Table 1 format and content
i) remove ~ symbols and use precise numbers ii) add ncRNAs summary (lncRNA, rRNA, tRNA were annotated in the study) iii) provide the number of N in scaffolds including features of Chr0 (HiC section in SM) iv) use more precise terms for rows labelled (Coverage, Gene coverage, Exon Coverage, Intron

coverage) or remove these rows.
The metrics related to protein coding genes are in the column "Contigs" but in the SM file the "reference genome" was masked and annotated. It is not clear what is the reference genome provided to the community. Please clarify this important point and provide a curated table with the full set of features related to the finalized reference genome.

Minor comments:

a) The genome polishing is a key step in the generation of high-quality consensus sequences. Indeed, the accuracy of the downstream definition of gene models depends on the genome quality. Sharing detailed expertise on this topic is important for current genome projects. Please, improve the description of the process in order to answer the questions.
Arrow was used for the first round of polishing. Did you use RSII data or only Sequel data ?
A second round of polishing was performed with illumina data. Which illumina dataset was used (please provide the sequencing depth used for the polishing) ? What were the BWA parameters for the mapping ? Did you applied some filtering processing on the BWA output ? To my knowledge, GATK is not a software allowing consensus polishing (pilon is one of these software). Did you develop a script to polish the consensus by using the GATK output?

b) To better understand the sequence conservation inside the CHS-L family, please provide a detailed summary of the 16 genes (genomic coordinates, gene and protein lengths, number of exons, etc.) but also additional analyses (in the SM file). Depending on the extend of the sequence conservation, I would suggest i) a table with a matrix containing the pairwise identity/similarity percentages (nucleotide and amino acid) ii) multiple sequence alignments iii) an analysis of the protein domains.

c) In the SM file, titles and descriptions of several tables/figures do not precisely describe the content.

Fig S5: several genetic maps were built, which genetic map was used to build the Figure ? Please, provide the number of markers in the genetic map and the number of markers plotted in the figure.
Fig S7: what are the numbers related to blue bars ? (length in nucleotides ?)
Fig S8: y-axis label is wrong, legend is incorrect. It is not a comparison of gene models but a comparison of gene model features.
Table S1: the current title is a "shortcut". An alternative could be "Summary of whole genome sequence data generated in the project". What is the genome size used to compute the sequencing depth? The label of the column "Long reads (bp)" is not consistent with the data.
Table S2: this is not a comparison of assembly results but a comparison of assembly metrics (no sequence comparison between assemblies).
Table S3: provide the BUSCO version, the lineage and mode parameters (not found elsewhere)
Table S5: It is not a statistical analysis but hit counts against databases
Table S6: Annotation of non-coding RNA genes. No, the current table is a summary of rRNA and tRNA gene features. Either modify the title or add the lncRNA features.
Please, check again titles and legend of tables and figures in the SM file.
d) L86 main text: Reciprocal pair-wise comparisons were performed to assess the evolution of candidate gene families. Unfortunately, the cited reference Li & Durbin is the reference of the BWA mapper. In the SM file, OrthoMCL was cited for the protein clustering. As an OrthoMCL run is not exactly a "Reciprocal pair-wise comparisons". The authors must clarify the protein family building process.
e) pacbio reads related to chloroplast and mitochondria were removed prior the FALCON assembly. The community would certainly appreciate that authors perform and provide the genome assemblies of the organelles as part of the reference genome of S. tora.
f) Comparative studies were performed using several legume genomes/proteomes (+ Vitis) but no information on the source of the data was provided. For several of these species, the genomes of several genotypes are now available and for some genotypes several genome versions were published. To avoid ambiguity, please clarify the source of the data used by adding a column to the

Table S9 with a persistent reference for all genomes used in the study (citation and/or url and/or accession number and/or doi)
g) provide the FALCON version used

Suggestions

L7: the N50 metric is not a feature fully relevant without the knowledge of genome size and number of chromosomes. In case of space constraints, I would suggest to replace scaffold N50 metric by the genome size and the number of chromosomes. It will provide a good level of detail on the contiguity of the assembly in the light of genome features.
L10: a CHS-L gene
Fig1C: use the corresponding subset of Table S10 (with actual numbers) instead of plots
Fig1D: add the last 4 digits of the corresponding gene IDs to gene model pictures
Fig2B: explain why y-axis has a space at 10 but not the bar plots in the corresponding region
Fig2C: add the number of genes in both clusters (as in Fig. S13 or in the text of the legend)

Reviewer #2 (Remarks to the Author):

I carefully checked this manuscript titled "Genome-Enabled Discovery of Anthraquinone Biosynthesis in Senna tora" including supplemental files. In this manuscript, authors sequenced whole genome of medicinal plant, Sena tora, to elucidate anthraquinone biosynthetic genes, and experimentally confirmed the function of key genes involved in emodin synthesis. I have some major/minor comments to this manuscript, authors should address and revise current manuscript.

Major comments
In this current manuscript, shikimate/mevalonate pathways for anthraquinone biosynthesis in Senna tora is not very clearly discussed. To discuss of this point, metabolomic analysis of primary metabolites showing metabolic shift is not enough, I think. Gene analysis, such as gene conservation, sequence alignment of genes involved in shikimate/mevalonate pathways between PKS type species and shikimate/mevalonate pathway type species can be considered to be included for this point.

Figure 2A: I am wondering whether authors have photos presenting same number of seeds or not. Stage 4 shows only one seed, it would be better to replace other photos, if authors have some.

Figure 2B: Results can be presented in "biosynthetic framework-based" figure in this figure or as a new supplemental figure. Because one of the discussion point is metabolic difference between biosynthesis branches between chrysophanol and emodin. At least all compounds in Figure 2B should be presented on the biosynthetic figure as Figure S1, otherwise for example obtusin and aloe-emodin are not clear for the readers how are they bio-synthesized.

LC-MS profiling: As authors presented, discussion of metabolic shift is an important point for understanding of productivity of anthraquinones during seed development. But although authors could detect many other specialized compounds such as polyphenolics, flavonoids and precursor of seed pigment which are detectable by authors LC-MS methods, I could not find data and discussion with them. Please include data of specialized compounds to this manuscript and data analysis for the discussion.

Figure 2C: I do not know whether this figure should be appeared in main figure or not. These clusters contain many genes, but text does not explain well this figure. Additionally, Figure 2C contains 14 time points, but it is hard to see as an integrated view between metabolite data. Please revise figures and text.

Figure 2D: This figure also can be moved to supplemental figure, if authors do not well-explain this metabolite results in the manuscript. At least, authors should explain results of some certain compounds but not like discussing with compound family as stated in the current manuscript.

Clustering analysis of transcriptomic data: I am sure why authors decided to evaluate with 9 clusters. Since genes are 13488 in total, nine cluster too small number for the clustering, I think.

L127-L133: Text for the result of transcriptomics data is somehow not very clear at least for me. Please revise text, and I recommend to add new supplemental figures of hypothesized anthraquinone biosynthesis and related genes (transferase, UGT, oxidoreductase..etc annotated from authors' correlation analysis) with metabolite name and gene ID. I think one/some of CHS-L genes were clustered into the cluster 3 or 6. Please mention this point here.

L137: "metabolic switch" seems to be weakly discussed. For me, this is not very clear.

Seed pigmentation: Metabolic switch of reduction of anthraquinones at latest stage (S7) during seed development, can be caused by metabolic flow for production of seed pigments?

Figure S9: Description of explaining why authors performed this analysis is not clear. Please revise text or remove this figure.

L84 sentence "To assess the evolution of candidate gene families,..": In my point of view, authors checked "gene conservation" and "species-specific genes" by cross species comparison, but not for the genomic analysis of evolution. Revise this and related sentences.

Figure 2D: I am wondering why anthraquinones are not included into this clustering analysis. I also think that this data might be not good for as a main figure, because authors have other important data for example enzymatic assay..etc.

CHS-L genes: I recommend to number CHS-L genes to make easier to see for the readers.

Introduction L48, "there has been little report of molecular..", Transcriptomic data analysis, Discussion, Citation: De novo transcriptome sequence of Senna tora provides insights into anthraquinone biosynthesis. By Kang PLoS One was not discussed and included in the reference list. Authors should carefully check their results and include to this manuscript.
Furthermore, other publications
Full-Length Transcriptome Survey and Expression Analysis of Cassia obtusifolia to Discover Putative Genes Related to Aurantio-Obtusin Biosynthesis, Seed Formation and Development, and Stress Response.
Deng Y, Zheng H, Yan Z, Liao D, Li C, Zhou J, Liao H.Int J Mol Sci. 2018 Aug 21;19(9):2476. doi: 10.3390/ijms19092476.
De novo sequencing, assembly and characterisation of Aloe vera transcriptome and analysis of expression profiles of genes related to saponin and anthraquinone metabolism.
Choudhri P, Rani M, Sangwan RS, Kumar R, Kumar A, Chhokar V.BMC Genomics. 2018 Jun 1;19(1):427. doi: 10.1186/s12864-018-4819-2.
Exploring drought stress-regulated genes in senna (Cassia angustifolia Vahl.): a transcriptomic approach.
Mehta RH, Ponnuchamy M, Kumar J, Reddy NR. Funct Integr Genomics. 2017 Jan;17(1):1-25. doi: 10.1007/s10142-016-0523-y. Epub 2016 Oct 5.
can be considered to be included to authors data interpretation and discussion to justify novelty of authors findings or support of authors results.

Bacterial anthraquinone: If authors can add sequence analysis and/or discussion of genes involved in anthraquinone biosynthesis in bacteria for the discussion of metabolic evolution between plants and

bacteria shortly, it would be very worth for the manuscript.

Figure S18, S20, S22, L184-L191: Because TIC chromatographic data is not showing nice shape, just EIC is enough for the figures, I think. But EIC chromatogram seems to be treated smoothing function. Text explaining results presented in these figures as well as figure legend are too short, it is difficult to understand this part.

Minor comments
Introduction, L18-L27: As an ancient medicine, other Chinese Senna (cassia) species such as obtusifolia and humilis, are also common as seed medicine containing anthraquinones. I suggest to revise an introduction slightly with explanation of widely used Senna plants and moving to Senna tora.

Reviewer #3 (Remarks to the Author):

There are literally thousands of medicinal plants and a daunting number of publications suggesting how their chemical compositions might contribute to their therapeutic value. Equally challenging is how one might prioritize which of these plants to investigate and why. It could be on the basis of some parameter of use – how common is a particular plant species used for a particular therapeutic intervention or how common is a medicinal plant across cultural traditions. Or, as in the current case, it might be based on fundamental questions about the origins of the putative novel chemistries associated with the plant. And yet, the current manuscript is so much more than this and is poised to be a standard for future genomic, chemical and biochemical assessment of medicinal plants specifically and all other plants in general.

The choice of studying Senna tora is based on its anthraquinone composition and outstanding questions about the biosynthetic origins of these compounds. Yes, it is easy to predict a polyketide origin for this class of compounds based on their structure and prior investigations in microbial systems, but of course one wonders about the origins of these compounds in plants. Are they derived from similar or different biosynthetic mechanisms?

The authors' approach to this question is comprehensive. First, they generate a very high resolution sequence for the Senna tora genome and discover that S. tora harbors an unusually large number of unique genes as evidenced by a large family of chalcone synthase (CHS) and chalcone synthase-like (CHS-like) genes. They follow this up with deep transcriptomic and metabolomic analysis of developing seeds where the anthraquinones accumulate. A cluster analysis of coordinately expressed unique genes points to several of the CHS-like genes that appear to be expressed temporally with the accumulation of some of the more structurally complex anthraquinone compounds. Lastly, biochemical characterization of one of the CHS-like genes demonstrates that the encoded enzyme has the capacity to generate two of the key biosynthetic precursor anthraquinones, atrochrysone and endocrocin.

One of the major conclusions from this study is that they have identified a CHS-like gene responsible for anthraquinone biosynthesis. Technically, they have not demonstrated this, although it is highly likely. What they actually show is that 1 of 16 CHS-like genes encodes for a catalytic activity that could contribute to the accumulation of key anthraquinones precursors. And this begs what are the biochemical capacities of the other 15 CHS-like genes? It isn't reasonable to expect knockouts for each member of the CHS-like gene family in S. tora and a comparison of what metabolic changes might occur in developing seed of the knockouts relative to wildtype. However, some comment as to how this one CHS-like enzyme compares to the others would be helpful. In fact, the data provided hints that all 16 of the CHS-like genes could possibly contribute to the accumulation of seed-specific species of anthraquinones and some comment to this effect would be appreciated.

The biochemical characterization of the one CHS-like gene is fine, but there are still many unanswered questions. Like how many products are actually generated? The impression from Fig. 3 is there is only two major products, but that is only because they have used extracted ion monitoring. What does the total ion monitoring spectrum look like? An equally important issue is what assay conditions were examined and how might alternative conditions supported unique reaction product readout? What cofactor requirements were examined?

I don’t think more actual experimentation is needed, only additional comments seem needed.

Joe Chappell

We appreciate your and the reviewers' thoughtful comments and suggestions on our manuscript, which were helpful in improving it. We have modified the manuscript based on the comments and are responding to each comment fully below.

Reviewer #1 (Remarks to the Author):

Major comments:

A) Data availability

- raw sequencing data: please provide the NCBI SRA accession numbers for all sequence datasets generated in the projet: illumina (WGS, HiC, RNASeq, GBS) and pacbio datasets. Adding missing datasets to Table S1 and adding a new column with accession numbers to S1 and S21 Tables will link the raw data to the minimum set of metadata ensuring re-usability.

Response: We have now provided the NCBI SRA accession numbers for all sequence datasets generated in the project. We inserted it in the corresponding position from S1 and S21.

- genome: please provide the Genbank accession number for the annotated reference genome

Response: We have provided the NCBI Genbank accession number (JAAIUW000000000) in the new manuscript.

- genetic map: please provide a table corresponding to the finalized genetic map (context sequences of markers, genetic position on linkage groups)

Response: We are now providing the final map including the linkage group (LG), SNP marker name (named based on contig ID and SNP position) and cM position as a large table file (Table S24).

- RNAseq data: The authors provide Tables for metabolic data (Tables S15 and S16) but the RNAseq counterpart is missing. Please provide the full expression matrix (raw count and TPM for all gene models). The expression matrix would be very useful to evaluate the data underlying the Fig2E heatmap. The members of the CHS-L cluster are certainly highly similar, some readers would appreciate to find the raw counts assigned to each member.

Response: We have now added the full expression matrix (raw count and TPM for all gene models) in the extended file (Table S29).

B) Building of pseudo chromosomes / reference genome

The paragraph describing the genome generation is the most ambiguous part of the manuscript (L58-L72).

Firstly, it describes the sequencing project (combination of illumina and pacbio approaches) more than the final result. Indeed, from main text only, readers can falsely understand that several assemblies, generated using various technologies, were integrated.

I would suggest rewriting in order to focus on the result: a pacbio-based assembly performed with falcon, scaffolded with HiC; two rounds of consensus polishing (1: arrow/pacbio; 2: see below my questions on the illumina polishing). Illumina data (reads and assemblies) as well as 454/Illumina BAC assemblies were used to assess the completeness of the reference genome assembly. BUSCO to assess the quality of the gene models.

Response: We have rewritten this section based on the reviewer's suggestion in the revised manuscript (line 14 to 18 on page 3).

The second ambiguity is related to the use of the genetic map. Usually, in plant genome projects, the genetic map is used for the final scaffolding of pseudomolecules and/or to orientate and number the pseudochromosomes. Here, pseudo chromosomes were built from HiC. It is acceptable but some breakpoints are visible in the HiC results (Fig S3 and S4) and the structure of the genome must be confirmed by the genetic map. Unfortunately, the Fig S7 does not support the claim "the 13 linkage grouped matched well to the 13 chromosomes". Indeed, the figure shows local matches but the positions on the chromosomes are not reported making impossible the assessment of the collinearity at a global level. In addition, the Figure is designed to report only matches on the expected chromosome.

To confirm that the genetic map is consistent with the structure of the chromosomes, please provide a figure where both coordinate systems are reported as well as all significant matches.

Response: To build pseudo chromosomes, we applied two methods, genetic map and Hi-C, of which the Hi-C method outperformed the genetic map. As we described in the text and Table S18, we could anchor about 401 Mb of sequences with 4455 SNP markers, which was about 100 Mb shorter than Hi-C based pseudo chromosomes. On the other hand, we did not identify any uniquely anchored contigs in the linkage based scaffolding compared to the Hi-C method. This was probably because F_2 population used in this study was too small to have enough recombination events.

As the reviewer pointed out, Fig S7 showed genetic markers and anchored contigs in genetic distances. Our intention was to display the association of SNP markers and anchored contigs in the linkage map context. In order to support "the 13 linkage grouped matched well to the 13 chromosomes" statement and provide additional information for the comments, we constructed 13 pseudo chromosomes using genetically anchored contigs, and they were compared to Hi-C pseudo chromosomes. The results showed pseudo chromosomes constructed by both methods were aligned with high collinearity except the alignment disruptions due to the additional contigs (~100 Mb across the genome) present in Hi-C pseudo but absent in the genetic map. One major discrepancy was detected in Chr9 (see dotted circle below and Table S8). In Hi-C derived pseudo chromosome, contig3 (11.9 Mb in length) in the beginning of Chr9 was split into two pieces, 8.8 Mb and 3.1 Mb, and the smaller contig was joined as an inversion to the end of the 8.8 Mb contig. However, in the genetic map, no marker was present in the 3.1 Mb region of contig3, which indicates that additional experiment may need to confirm this split.

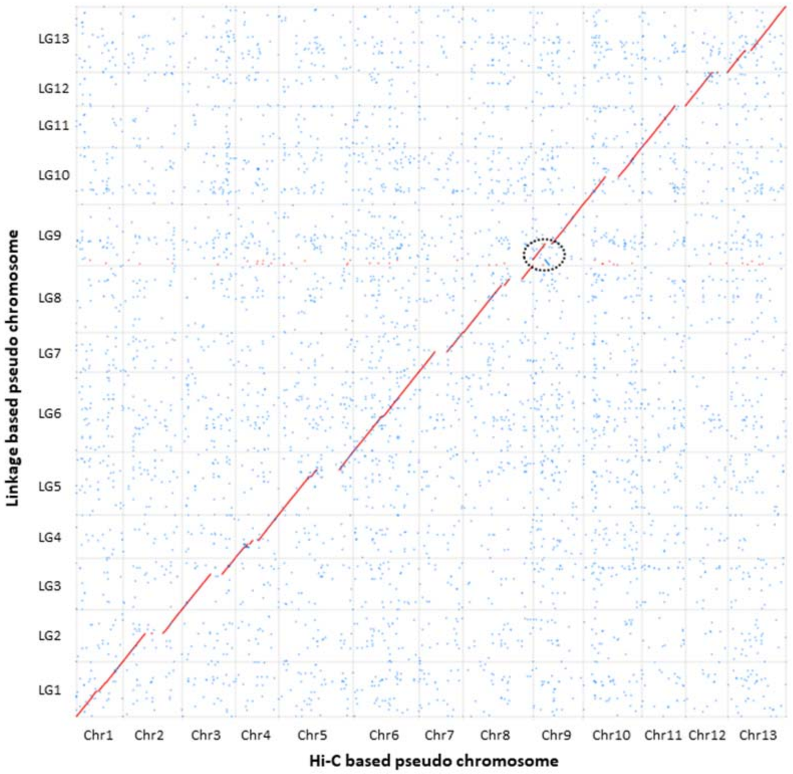

C) Table 1 format and content

i) remove ~ symbols and use precise numbers

Response: We removed ~ symbols and used the precise numbers in Table 1.

ii) add ncRNAs summary (lncRNA, rRNA, tRNA were annotated in the study)

Response: We added ncRNAs summary in the Table 1.

iii) provide the number of N in scaffolds including features of Chr0 (HiC section in SM)

Response: We added N (%) in scaffolds including Chr00 in Table S26.

iv) use more precise terms for rows labelled (Coverage, Gene coverage, Exon Coverage, Intron coverage) or remove these rows.

Response: We removed these rows in the revised version.

The metrics related to protein coding genes are in the column "Contigs" but in the SM file the "reference genome" was masked and annotated. It is not clear what is the reference genome provided to the community. Please clarify this important point and provide a curated table with the full set of features related to the finalized reference genome.

Response: We used "reference genome" for functional annotation of *S. tora*. We moved protein coding genes in the column "Superscaffolds" and added non-coding genes including lncRNA, rRNA and tRNA in Table 1.

Minor comments:

a) The genome polishing is a key step in the generation of high-quality consensus sequences. Indeed, the accuracy of the downstream definition of gene models depends on the genome quality. Sharing detailed expertise on this topic is important for current genome projects. Please, improve the description of the process in order to answer the questions.

Arrow was used for the first round of polishing. Did you use RSII data or only Sequel data ?

Response: We used Sequel data for Arrow polishing (added this info in line 18 on page 2 in the SM file).

A second round of polishing was performed with illumina data. Which illumina dataset was used (please provide the sequencing depth used for the polishing) ?

Response: We used 101.2X of PE_200bp libraries for the second polishing (added info in line 21 on page 2 in the SM file).

What were the BWA parameters for the mapping?

Response: We used the default parameters of BWA (added this info in line 26 on page 3 in the SM file).

Did you applied some filtering processing on the BWA output ? To my knowledge, GATK is not a software allowing consensus polishing (pilon is one of these software). Did you develop a script to polish the consensus by using the GATK output?

Response: Yes, we did. At the time of performing *S. tora* assembly, there were many errors when performing Pilon analysis. As an alternative, a variant call was made using GATK, and then filtering was performed using GATK's FastaAlternativeReferenceMaker (added in line 27 on page 3 in the SM file).

b) To better understand the sequence conservation inside the CHS-L family, please provide a detailed summary of the 16 genes (genomic coordinates, gene and protein lengths, number of exons, etc.) but also additional analyses (in the SM file). Depending on the extend of the sequence conservation, I would suggest i) a table with a matrix containing the pairwise identity/similarity percentages (nucleotide and amino acid) ii) multiple sequence alignments iii) an analysis of the protein domains.

Response: We have provided a detailed summary (CDS and protein lengths, number of exons and introns, molecular weight, *etc*.) of the 16 genes in the new version. Also, as Dr. Chappell mentioned, we chose *S. tora* based on its anthraquinone composition and fundamental questions about the biosynthetic origins of these compounds. Through the *S. tora* genome project, we were able to answer this question and demonstrate it definitively for the first time in plants. We plan to carry out an overall study on the functional differences, 3D structure, and more of the CHS-L genes of *S. tora* in the future.

c) In the SM file, titles and descriptions of several tables/figures do not precisely describe the content.

Response: We have revised the descriptions of figure legends and tables in the supporting files.

Fig S5: several genetic maps were built, which genetic map was used to build the Figure? Please, provide the number of markers in the genetic map and the number of markers plotted in the figure.

Response: This figure was drawn based on our final genetic map (provided as a supplementary Table S24), which was summarized in Table S18 including the number of markers per linkage group, genetic length, number of contigs *etc*.

Fig S7: what are the numbers related to blue bars? (length in nucleotides?)

Response: Yes, the numbers indicate length in nucleotides as shown on the blue bars. This information has been added to the figure legend.

Fig S8: y-axis label is wrong, legend is incorrect. It is not a comparison of gene models but a comparison of gene model features.

Response: We have revised legend and y-axis label.

Table S1: the current title is a "shortcut". An alternative could be "Summary of whole genome sequence data generated in the project".

Response: We have modified it to “Summary of whole genome sequence data generated in the project”.

What is the genome size used to compute the sequencing depth?

Response: 547 Mb (an estimated genome size of *S. tora*).

The label of the column "Long reads (bp)" is not consistent with the data.

Response: We removed it in the revised manuscript.

Table S2: this is not a comparison of assembly results but a comparison of assembly metrics (no sequence comparison between assemblies).

Response: We have modified it to “Summary statistics of the *S. tora* genome assemblies”.

Table S3: provide the BUSCO version, the lineage and mode parameters (not found elsewhere)

Response: We have provided the BUSCO version and the lineage in the Table S3.

Table S5: It is not a statistical analysis but hit counts against databases

Response: We have modified it to “Functional annotations of protein-coding genes in *S. tora*”.

Table S6: Annotation of non-coding RNA genes. No, the current table is a summary of rRNA and tRNA gene features. Either modify the title or add the lncRNA features.

Response: We have modified it to “Annotation of rRNA and tRNA genes in *S. tora*”.

Please, check again titles and legend of tables and figures in the SM file.

Response: We carefully review the titles and legend of tables and figures in the manuscript to improve our manuscript quality.

d) L86 main text: Reciprocal pair-wise comparisons were performed to assess the evolution of candidate gene families. Unfortunately, the cited reference Li & Durbin is the reference of the BWA mapper. In the SM file, OrthoMCL was cited for the protein

clustering. As an OrthoMCL run is not exactly a "Reciprocal pair-wise comparisons". The authors must clarify the protein family building process.

Response: We have fixed reference in the main text and clarified the protein family building process in the SM file.

e) pacbio reads related to chloroplast and mitochondria were removed prior the FALCON assembly. The community would certainly appreciate that authors perform and provide the genome assemblies of the organelles as part of the reference genome of *S. tora*.

Response: As with the *S. tora* genome annotation project, we provided the organelles genome information with NCBI.

f) Comparative studies were performed using several legume genomes/proteomes (+ Vitis) but no information on the source of the data was provided. For several of these species, the genomes of several genotypes are now available and for some genotypes several genome versions were published. To avoid ambiguity, please clarify the source of the data used by adding a column to the Table S9 with a persistent reference for all genomes used in the study (citation and/or url and/or accession number and/or doi)

Response: We have provided the source of the data in Table S9.

g) provide the FALCON version used

Response: We used FALCON v0.4 and described it in the revised manuscript (line 16 on page 3).

Suggestions

L7: the N50 metric is not a feature fully relevant without the knowledge of genome size and number of chromosomes. In case of space constraints, I would suggest to replace scaffold N50 metric by the genome size and the number of chromosomes. It will provide a good level of detail on the contiguity of the assembly in the light of genome features.

Response: We added the assembled genome size and the number of chromosomes in the revised manuscript (line 4 to 5 on page 2).

L10: a CHS-L gene

Response: Done.

Fig1C: use the corresponding subset of Table S10 (with actual numbers) instead of plots

Response: Done.

Fig1D: add the last 4 digits of the corresponding gene IDs to gene model pictures

Response: Done.

Fig2B: explain why y-axis has a space at 10 but not the bar plots in the corresponding region

Response: The bar plots are plotted relative to the y-axis, only the spaces are not displayed as a method of visualization.

Fig2C: add the number of genes in both clusters (as in Fig. S13 or in the text of the legend)

Response: We have added the number of genes in both clusters in the graph.

Reviewer #2 (Remarks to the Author):

Major comments

In this current manuscript, shikimate/mevalonate pathways for anthraquinone biosynthesis in Senna tora is not very clearly discussed. To discuss of this point, metabolomic analysis of primary metabolites showing metabolic shift is not enough, I think. Gene analysis, such as gene conservation, sequence alignment of genes involved in shikimate/mevalonate pathways between PKS type species and shikimate/mevalonate pathway type species can be considered to be included for this point.

Response: Although anthraquinone biosynthesis was speculated to occur via shikimate/mevalonate pathway several decades ago using carbon labelled precursor substrates, no evidence is available at the genetic level. Thus, at this point it is difficult to differentiate the metabolic pathway responsible for anthraquinone biosynthesis based on protein sequence analysis of the enzymes involved in the precursor pathways.

Figure 2A: I am wondering whether authors have photos presenting same number of seeds or not. Stage 4 shows only one seed, it would be better to replace other photos, if authors have some.

Response: Our intention was that the seeds were the largest in Stage 4, and only one was placed to take pictures. Unfortunately, there are no photos taken with two placed on Stage 4. We added text in the legend that these are representative images.

Figure 2B: Results can be presented in "biosynthetic framework-based" figure in this figure or as a new supplemental figure. Because one of the discussion point is metabolic difference between biosynthesis branches between chrysophanol and emodin. At least all compounds in Figure 2B should be presented on the biosynthetic figure as Figure S1, otherwise for example obtusin and aloe-emodin are not clear for the readers how are they bio-synthesized.

Response: The previous Figure S1 is now replaced with a new figure showing possible biosynthetic routes to all the metabolites mentioned in Figure 2B.

LC-MS profiling: As authors presented, discussion of metabolic shift is an important point for understanding of productivity of anthraquinones during seed development. But although authors could detect many other specialized compounds such as polyphenolics, flavonoids and precursor of seed pigment which are detectable by authors LC-MS methods, I could not find data and discussion with them. Please include data of specialized compounds to this manuscript and data analysis for the discussion.

Response: We agree with the reviewer's point on other metabolites analysis from the different stages of seeds. We could easily profile other specialized metabolites such as polyphenols/flavonoids and seed pigments using the methods used for anthrqauinones analysis. However, the manuscript is solely focused on anthraquinone biosynthesis rather than other specialized metabolites. Thus, we feel that adding additional metabolomics data on other specialized metabolites might make the manuscript less coherent. Notwithstanding this, we have expanded the text and analysis of other specialized metabolic genes in terms of their expression patterns in developing seeds in the revised manuscript (line 42 on page 4 to line 8 on page 5).

Figure 2C: I do not know whether this figure should be appeared in main figure or not. These clusters contain many genes, but text does not explain well this figure. Additionally, Figure 2C contains 14 time points, but it is hard to see as an integrated view between metabolite data. Please revise figures and text.

Response: Figs. 2C-D now show expression patterns for genes induced during seed maturation in a manner similar to anthraquinone accumulation. To highlight metabolic genes in these two clusters, we analyzed enriched metabolic domains among genes in these clusters.

Figure 2D: This figure also can be moved to supplemental figure, if authors do not well-explain this metabolite results in the manuscript. At least, authors should explain results of some certain compounds but not like discussing with compound family as stated in the current manuscript.

Response: As suggested, we moved this figure to the supplementary materials and also revised the text to better describe the patterns of measured compounds (line 5 to 9 on page 5).

Clustering analysis of transcriptomic data: I am sure why authors decided to evaluate with 9 clusters. Since genes are 13488 in total, nine cluster too small number for the clustering, I think.

Response: The choice of 9 clusters was empirically driven from the heatmap results of the DEG analysis and all 13,488 are represented in them.

L127-L133: Text for the result of transcriptomics data is somehow not very clear at least for me. Please revise text, and I recommend to add new supplemental figures of hypothesized anthraquinone biosynthesis and related genes (transferase, UGT, oxidoreductase..etc annotated from authors' correlation analysis) with metabolite name and gene ID. I think one/some of CHS-L genes were clustered into the cluster 3 or 6. Please mention this point here.

Response: We have expanded the description of the transcriptomics data analysis (line 41 on page 4 to line 7 on page 5) and performed pathway enrichment analysis for clusters 3 and 6. As previously mentioned, cluster 6 is enriched in potential tailoring enzymes such as transferases, UDP-glycosyltransferases, and oxidoreductases. The CHS-L genes were not included in clusters 3 and 6. In order to find additional enzymes involved in the pathway, we need to perform additional analyses of quantifying transcript levels as well as biochemistry, which we feel is beyond the scope of this report.

L137: ”metabolic switch” seems to be weakly discussed. For me, this is not very clear.

Response: We have updated the language in the manuscript to reflect a general decrease in metabolic gene expression between stages 4 and 5 and have added an additional supplemental figure to support this point (Figure S15). We removed the discussion of metabolic switch.

Seed pigmentation: Metabolic switch of reduction of anthraquinones at latest stage (S7) during seed development, can be caused by metabolic flow for production of seed pigments?

Response: Most of the anthraquinones detected at the early phase of seed developments were modified by tailoring enzymes such as hydroxylases, methyltransferases, and glycosyltransferases to produce highly modified metabolites (See possible pathways in Fig. S1). Highly modified metabolites were only predominantly present at the S7. We don't have any evidence/results to show the reason for reduction of anthraquinone at the late stages of seed development.

Figure S9: Description of explaining why authors performed this analysis is not clear. Please revise text or remove this figure.

Response: We removed this figure.

L84 sentence "To assess the evolution of candidate gene families,..": In my point of view, authors checked "gene conservation" and "species-specific genes" by cross species comparison, but not for the genomic analysis of evolution. Revise this and related sentences.

Response: We have revised this and related sentences in the revision (line 41 on page 3).

Figure 2D: I am wondering why anthraquinones are not included into this clustering analysis. I also think that this data might be not good for as a main figure, because authors have other important data for example enzymatic assay..etc.

Response: Figure 2D has been replaced with figures showing enriched metabolic domains from clusters 3 and 6, which show similar expression patterns to anthraquinone accumulation during seed maturation. The figure representing the profile of primary metabolites has been moved to a supplemental figure (Fig. S17).

CHS-L genes: I recommend to number CHS-L genes to make easier to see for the readers.

Response: We have numbered CHS-L genes in the revised manuscript.

Introduction L48, "there has been little report of molecular..", Transcriptomic data analysis, Discussion, Citation: De novo transcriptome sequence of Senna tora provides insights into anthraquinone biosynthesis. By Kang PLoS One was not discussed and included in the reference list. Authors should carefully check their results and include to this manuscript.

Furthermore, other publications

Full-Length Transcriptome Survey and Expression Analysis of Cassia obtusifolia to Discover Putative Genes Related to Aurantio-Obtusin Biosynthesis, Seed Formation and Development, and Stress Response.

Deng Y, Zheng H, Yan Z, Liao D, Li C, Zhou J, Liao H.Int J Mol Sci. 2018 Aug 21;19(9):2476. doi: 10.3390/ijms19092476.

De novo sequencing, assembly and characterisation of Aloe vera transcriptome and analysis of expression profiles of genes related to saponin and anthraquinone metabolism.

Choudhri P, Rani M, Sangwan RS, Kumar R, Kumar A, Chhokar V.BMC Genomics. 2018 Jun 1;19(1):427. doi: 10.1186/s12864-018-4819-2.

Exploring drought stress-regulated genes in senna (Cassia angustifolia Vahl.): a transcriptomic approach.

Mehta RH, Ponnuchamy M, Kumar J, Reddy NR. Funct Integr Genomics. 2017 Jan;17(1):1-25. doi: 10.1007/s10142-016-0523-y. Epub 2016 Oct 5.

can be considered to be included to authors data interpretation and discussion to justify novelty of authors findings or support of authors results.

Response: We now cite these articles in the introduction (line 4 to 5 on page 3).

Bacterial anthraquinone: If authors can add sequence analysis and/or discussion of genes involved in anthraquinone biosynthesis in bacteria for the discussion of metabolic evolution between plants and bacteria shortly, it would be very worth for the manuscript.

Response: We have added anthraquinone biosynthesis in bacteria briefly in the Discussion section of the revised manuscript (line 27 to 33 on page 6).

Figure S18, S20, S22, L184-L191: Because TIC chromatographic data is not showing nice shape, just EIC is enough for the figures, I think. But EIC chromatogram seems to be treated smoothing function. Text explaining results presented in these figures as well as figure legend are too short, it is difficult to understand this part.

Response: EIC chromatograms are not treated with a smoothing function. These are simply extracted EIC from TIC. The figure legends are now extended with more explanation in the revised manuscript.

Minor comments

Introduction, L18-L27: As an ancient medicine, other Chinese Senna (cassia) species such as obtusifolia and humilis, are also common as seed medicine containing anthraquinones. I suggest to revise an introduction slightly with explanation of widely used Senna plants and moving to Senna tora.

Response: The information is added in the introduction with citation (line 21 to 22 on page 2).

Reviewer #3 (Remarks to the Author):

One of the major conclusions from this study is that they have identified a CHS-like gene responsible for anthraquinone biosynthesis. Technically, they have not demonstrated this, although it is highly likely. What they actually show is that 1 of 16 CHS-like genes encodes for a catalytic activity that could contribute to the accumulation of key anthraquinones precursors. And this begs what are the biochemical capacities of

the other 15 CHS-like genes? It isn't reasonable to expect knockouts for each member of the CHS-like gene family in S. tora and a comparison of what metabolic changes might occur in developing seed of the knockouts relative to wildtype. However, some comment as to how this one CHS-like enzyme compares to the others would be helpful. In fact, the data provided hints that all 16 of the CHS-like genes could possibly contribute to the accumulation of seed-specific species of anthraquinones and some comment to this effect would be appreciated.

Response: Inferring from the gene expression values below, the three genes (CHS-L16, CHS-L1, and CHS-L15) are unlikely to contribute to anthraquinone biosynthesis in seeds. At any stage of the seed, there are a total of seven genes (CHS-L13, CHS-L3, CHS-L2, CHS-L11, CHS-L10, CHS-L9 and CHS-L6) with a TPM over 1000, so it can be expected to make a significant contribution to anthraquinone biosynthesis. Also, since two genes (CHS-L2 and CHS-L6), such as a CHS-L9, show more than 1000 expression values in Stage 4, it may be meaningful to verify the function of these genes. The following 16 CHS-L gene expression values are provided in Table S22.

Gene_ID	Gene Expression Value (TPM)						
	S1	S2	S3	S4	S5	S6	S7
Sto07g228380 (CHS-L16)	0	0	0	0	0	0	0
Sto02g027180 (CHS-L1)	0	0	0.06	0	0	0.11	0.05
Sto07g228370 (CHS-L15)	0.24	0	0	0	0	0	0
Sto07g228310 (CHS-L13)	2096.42	569.03	214.12	425.35	1.535	0.52	1.98
Sto07g228240 (CHS-L8)	475.80	429.96	37.77	1.72	0	0.11	1.01
Sto07g228160 (CHS-L3)	1618.38	655.23	25.51	7.15	0.06	0.06	0
Sto07g228180 (CHS-L4)	7.93	4.735	0.205	0.16	0	0	0
Sto07g228150 (CHS-L2)	5324.20	3288.64	1248.77	1575.40	21.33	0.19	1.08
Sto07g228270 (CHS-L11)	1432.91	613.92	182.52	89.99	0.34	0.87	5.25
Sto07g228260 (CHS-L10)	3246.98	1451.94	325.57	295.97	1.01	0.34	3.14
Sto07g228320 (CHS-L14)	119.29	125.24	154.36	104.18	0.59	1.67	11.84
Sto07g228300 (CHS-L12)	59.63	46.03	53.13	22.61	0.84	1.42	4.57
Sto07g228230 (CHS-L7)	263	186.74	187.37	106.19	1.28	1.76	11.46
Sto07g228210 (CHS-L5)	10.91	350.99	463.93	281.29	0.56	0.06	0
Sto07g228250 (CHS-L9)	1055.83	1053.35	1274.55	2696.51	23.22	2.04	5.40
Sto07g228220 (CHS-L6)	3.54	1289.72	3110.70	5017.52	32.58	0.87	20.37

The biochemical characterization of the one CHS-like gene is fine, but there are still many unanswered questions. Like how many products are actually generated? The impression from Fig. 3 is there is only two major products, but that is only because they have used extracted ion monitoring.

Response: We have checked EIC for all possible intermediates or final metabolites produced after eight manonyl-CoA condensation reactions in the pathway following chrysophanol production and the pathway leading to emodin production (see the pathway diagram in Fig. S1). The EIC for atrochrysone carboxylic acid and endocrocin anthrone (data presented in this manuscript) showed prominent peak in the EIC. None of the other metabolites showed peaks in the EIC. Thus, no other octaketide was detected in the reaction mixture. However, type III PKS/Octaketide synthases can condense different numbers of malonyl-CoAs to produce different metabolites such as triketide, tetraketide, pentaketide, hexaketide, heptaketide, though we have not conducted these experiments. We have clarified this info in the revised manuscript (line 9 to 13 on page 6).

What does the total ion monitoring spectrum look like?

Response: In the TIC of the reaction sample, it is very difficult to see distinct separate peaks from the control TIC (see below). Thus, we have not added TIC data in the manuscript. We can see difference only in the EIC. We have added these data as supplemental figures and describe them in the main text (line 32 on page 5 to line 13 on page 6).

CHS-L reaction with malonyl-CoA and control reaction with denatured protein:

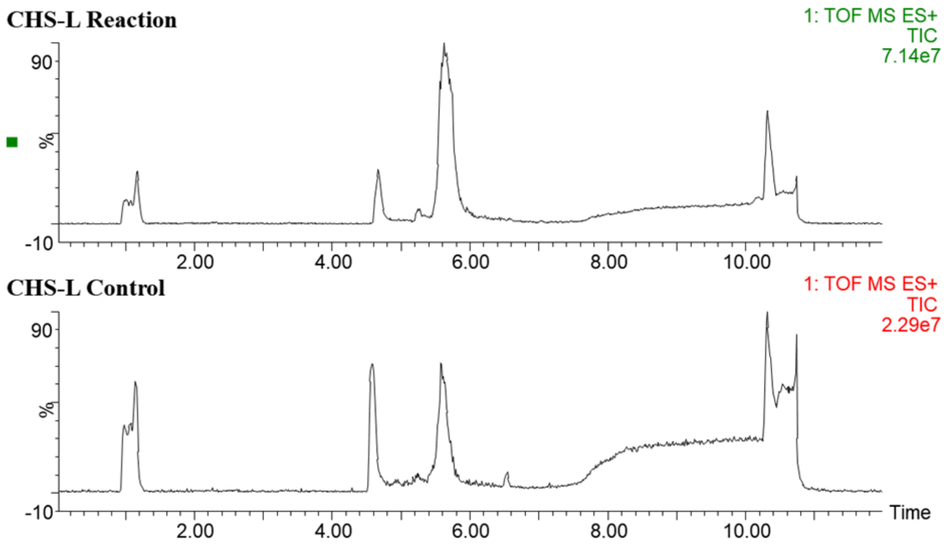

CHS-L reaction with ^{13}C-malonyl-CoA and control reaction with denatured protein:

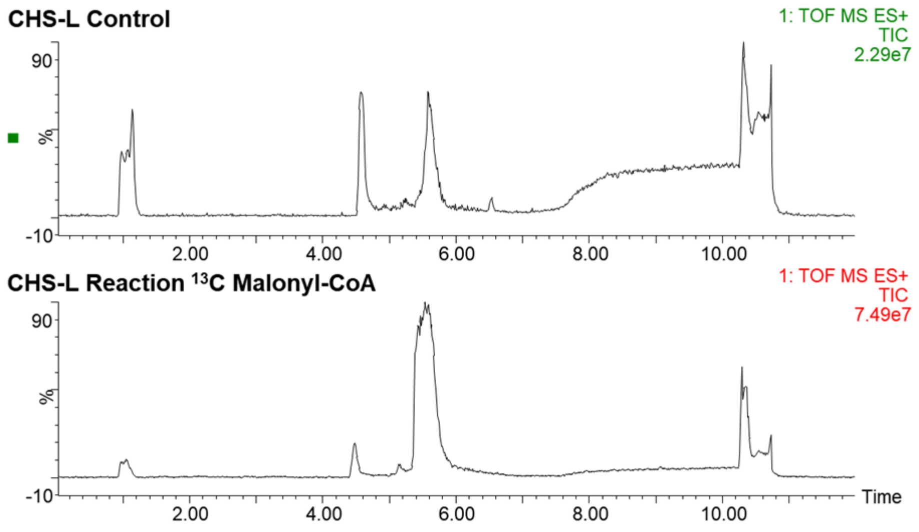

An equally important issue is what assay conditions were examined?

Response: Enzyme reactions were carried out in following conditions:

1 ml volume containing 5 mM of malonyl-CoA, 10 mM of $MgCl_2$, and 10 ug/ml of pure protein in 100 mM phosphate buffer saline (pH 7.5). An identical reaction mixture containing the same amount of heat-denatured protein served as a negative control. A separate reaction was carried out with the same reaction constituents with additional 1 mM of NADPH as a co-factor. Similarly, assays were carried out with identical reaction components except for malonyl-CoA, which was replaced with the same amount of $^{13}C_3$-malonyl-CoA. All reaction mixtures were incubated at 30 °C for 6 h, and stopped by heating the reaction mixture at 85 °C for 3 min. This method is described in SI.

How might alternative conditions supported unique reaction product readout?

Response: We have not performed alternative reaction conditions varying the buffer, pH, or temperature to see the product profile. We employed commonly used reaction condition for production of type III PKS metabolites. We have clarified this in the revised manuscript (line 9 to 13 on page 6).

What cofactor requirements were examined?

Response: We have carried out additional reaction using NADPH to see if the product profile varies. However, no variation in product was observed. This is mentioned in penultimate paragraph of the main text (line 9 to 13 on page 6). Since the results were not different, we have not presented that data in the manuscript.

I don’t think more actual experimentation is needed, only additional comments seem needed.

Joe Chappell

Reviewer #1 (Remarks to the Author):

The authors have substantially responded to my concerns on the genomic part of the manuscript. In addition, most, if not all, of my comments have been satisfactorily addressed.

Jerome Gouzy

Reviewer #2 (Remarks to the Author):

I found that authors largely revised their manuscript properly with addressing to almost all comments which I pointed previously. Just only one point left that authors can re-consider,
> LC-MS profiling: As authors presented, discussion of metabolic shift is an important point for understanding of productivity of anthraquinones during seed development. But although authors could detect many other specialized compounds such as polyphenolics, flavonoids and precursor of seed pigment which are detectable by authors LC-MS methods, I could not find data and discussion with them. Please include data of specialized compounds to this manuscript and data analysis for the discussion.
Response: We agree with the reviewer's point on other metabolites analysis from the different stages of seeds. We could easily profile other specialized metabolites such as polyphenols/flavonoids and seed pigments using the methods used for anthrqauinones analysis. However, the manuscript is solely focused on anthraquinone biosynthesis rather than other specialized metabolites. Thus, we feel that adding additional metabolomics data on other specialized metabolites might make the manuscript less coherent. Notwithstanding this, we have expanded the text and analysis of other specialized metabolic genes in terms of their expression patterns in developing seeds in the revised manuscript (line 42 on page 4 to line 8 on page 5).
>> My suggestion is that if authors can show other secondary metabolite data, authors can explain and emphasize that correlation of expression pattern of candidate genes are specifically correlated to metabolic pattern of your target anthrqauinones but not other specialized metabolites. For example, heatmap data of metabolite pattern like heatmap which authors presented for gene expression data, can be applied as supplemental table and figure.

Reviewer #3 (Remarks to the Author):

My original review of this manuscript stipulated how significant I considered this work. It is a comprehensive genetic exploration into the biosynthesis of a very deserving class of natural products in plants. Anthraquinones are known for their biological and therapeutic activities and their biosynthesis in microbes is well appreciated. The primary insight provided in this work is that plants also appear to utilize similar biosynthetic mechanisms for polyketide biosynthesis as in microbes. This is significant because prior to this work the prevailing wisdom was plants might use a complete different mechanism.

While the details of assembling the genome are important, the more compelling justification for doing such is that it provides insights as to how a novel class of chalcone synthase-like genes might have evolved and given rise to this novel biosynthetic capactity in Senna tora. So the genomic work revealed 16 copies of the CHS-like genes and the authors have gone on to functionally characterize one of these. But this is where I might disagree a bit with the authors. They do present evidence for CHS-L9 ability to generate one of the key anthraquinone intermediates. But we still don't know how significant this data is. Does only one of the 16 CHS-L genes encode for this functionality? I mentioned this point in my earlier review and suppose I didn't communicate my point very well. If one takes a hard look as the biochemistry performed, the generation of the atrochrysone and endocrocin reaction products in vitro are pretty anemic. Hence, I wondered if any of the other 15 CHS-L genes might

encode for a more robust activity. This question still remains in the current manuscript and what I encouraged before and now is that the authors don't box themselves in – it isn't necessary. CHS-L9 does catalyze the biosynthesis of atrochrysone and endocrocin, but any of the other CHS-L enzymes might also. Let me further this thinking by suggesting the active atrochrysone/endocrocin synthase could be a heterodimeric enzyme.

There is one additional point that I don't expect the authors to address, but offer it for perspective. It has to do with the temporal relationship of things. If one examines the accumulation patterns of the different anthraquinones over the 7 stages of seed development, one sees that some accumulate earlier, some are present throughout and some tend to peak at the later stages of development. Hence, the notion that the transcripts for the biosynthesis should reflect an induction up to stage 4 is not particularly compelling. If this were so, then the first derivative of the accumulation pattern should give the changes expected for biosynthetic activity, assuming degradation or turnover of the metabolite was negligible. It then follows that the second derivative of the accumulation pattern should give the change in the transcript level encoding for a key enzyme, if turnover of the accumulating compound was negligible and the half-life of the enzyme and mRNA is constant.

Bottom line, the authors don't need to claim that CHS-L9 is THE key gene encoding for THE key enzyme. The work certain demonstrates this exciting possibility and it looks like CHS-L9 does contribute to anthraquinone accumulation in seeds of S. tora.

Joe Chappell

REVIEWERS' COMMENTS

Reviewer #1 (Remarks to the Author):

The authors have substantially responded to my concerns on the genomic part of the manuscript. In addition, most, if not all, of my comments have been satisfactorily addressed.

Jerome Gouzy

Response: Thank you for confirming and your time and effort in reviewing our manuscript.

Reviewer #2 (Remarks to the Author):

I found that authors largely revised their manuscript properly with addressing to almost all comments which I pointed previously. Just only one point left that authors can re-consider,
> LC-MS profiling: As authors presented, discussion of metabolic shift is an important point for understanding of productivity of anthraquinones during seed development. But although authors could detect many other specialized compounds such as polyphenolics, flavonoids and precursor of seed pigment which are detectable by authors LC-MS methods, I could not find data and discussion with them. Please include data of specialized compounds to this manuscript and data analysis for the discussion.
Response: We agree with the reviewer's point on other metabolites analysis from the different stages of seeds. We could easily profile other specialized metabolites such as polyphenols/flavonoids and seed pigments using the methods used for anthrqauinones analysis. However, the manuscript is solely focused on anthraquinone biosynthesis rather than other specialized metabolites. Thus, we feel that adding additional metabolomics data on other specialized metabolites might make the manuscript less coherent. Notwithstanding this, we have expanded the text and analysis of other specialized metabolic genes in terms of their expression patterns in developing seeds in the revised manuscript (line 42 on page 4 to line 8 on page 5).
>> My suggestion is that if authors can show other secondary metabolite data, authors can explain and emphasize that correlation of expression pattern of candidate genes are specifically correlated to metabolic pattern of your target anthrqauinones but not other specialized metabolites. For example, heatmap data of metabolite pattern like heatmap which authors presented for gene expression data, can be applied as supplemental table and figure.

Response: We appreciate the reviewer's point about the possibility of using profiles of other specialized metabolites to further filter for possible candidate genes for anthraquinone biosynthesis. However, what we reported in this manuscript is a targeted LC-MS for anthraquinones and we don't have the data for other specialized metabolites. To address this comment fully, we would have to redo the experiments and perform non-targeted LC-MS, which we don't have the resources to do at this time. In addition, even if we did these additional

experiments, we are not certain how the potential lack of correlation between gene expression and other specialized metabolites would help us narrow down the candidate genes. We believe that not having this data does not take away from the main findings we are reporting in this manuscript.

Reviewer #3 (Remarks to the Author):

My original review of this manuscript stipulated how significant I considered this work. It is a comprehensive genetic exploration into the biosynthesis of a very deserving class of natural products in plants. Anthraquinones are known for their biological and therapeutic activities and their biosynthesis in microbes is well appreciated. The primary insight provided in this work is that plants also appear to utilize similar biosynthetic mechanisms for polyketide biosynthesis as in microbes. This is significant because prior to this work the prevailing wisdom was plants might use a complete different mechanism.

While the details of assembling the genome are important, the more compelling justification for doing such is that it provides insights as to how a novel class of chalcone synthase-like genes might have evolved and given rise to this novel biosynthetic capactity in Senna tora. So the genomic work revealed 16 copies of the CHS-like genes and the authors have gone on to functionally characterize one of these. But this is where I might disagree a bit with the authors. They do present evidence for CHS-L9 ability to generate one of the key anthraquinone intermediates. But we still don't know how significant this data is. Does only one of the 16 CHS-L genes encode for this functionality? I mentioned this point in my earlier review and suppose I didn't communicate my point very well. If one takes a hard look as the biochemistry performed, the generation of the atrochrysone and endocrocin reaction products in vitro are pretty anemic. Hence, I wondered if any of the other 15 CHS-L
genes might encode for a more robust activity. This question still remains in the current manuscript and what I encouraged before and now is that the authors don't box themselves in – it isn't necessary. CHS-L9 does catalyze the biosynthesis of atrochrysone and endocrocin, but any of the other CHS-L enzymes might also. Let me further this thinking by suggesting the active atrochrysone/endocrocin synthase could be a heterodimeric enzyme.

There is one additional point that I don't expect the authors to address, but offer it for perspective. It has to do with the temporal relationship of things. If one examines the accumulation patterns of the different anthraquinones over the 7 stages of seed development, one sees that some accumulate earlier, some are present throughout and some tend to peak at the later stages of development. Hence, the notion that the transcripts for the biosynthesis should reflect an induction up to stage 4 is not particularly compelling. If this were so, then the first derivative of the accumulation pattern should give the changes expected for biosynthetic activity, assuming degradation or turnover of the metabolite was negligible. It then follows that the second derivative of the accumulation pattern should give the change in the transcript level encoding for a key enzyme, if turnover of the accumulating compound was negligible and the half-life of the enzyme and mRNA is constant.

Bottom line, the authors don't need to claim that CHS-L9 is THE key gene encoding for THE key enzyme. The work certain demonstrates this exciting possibility and it looks like CHS-L9 does contribute to anthraquinone accumulation in seeds of S. tora.

Joe Chappell

Response: We appreciate and agree with the points raised by Dr. Chappell. We have addressed the comments by revising the text in the Abstract ("a CHS-L gene contributing to the biosynthesis of anthraquinones", page 2, lines 8-9), Introduction ("experimentally confirm a key gene responsible for biosynthesis of an anthraquinone scaffold", page 3, line 5), and Results ("It is possible that other CHS-L genes might also participate in anthraquinone biosynthesis." Page 6, lines 18-19).
